# A structurally unique effector shared between vascular wilt fungi drives cotton and olive defoliation

**DOI:** 10.1038/s41467-026-74504-z

**Published:** 2026-06-21

**Authors:** Andrea Doddi, Gabriel Lorencini Fiorin, Jinling Li, Ilaria Zannini, Yukiyo Sato, Giulia Lancia, Giacomo Giuliari, Carmen Gómez-Lama Cabanás, Antonio Valverde-Corredor, Tingli Liu, Hui Tian, Grardy C. M. van den Berg, Jesús Mercado-Blanco, Baolong Zhang, Michael F. Seidl, Martijn Rep, Wessel Groot, Maria Carmela Bonaccorsi Di Patti, Francesca Troilo, Adele Di Matteo, Giorgio Giardina, Massimo Reverberi, Longfu Zhu, Luigi Faino, Bart P. H. J. Thomma

**Affiliations:** 1 Department of Environmental Biology, Sapienza University of Rome, Rome, Italy; 2 University of Cologne, Institute for Plant Sciences, Cluster of Excellence on Plant Sciences (CEPLAS), Cologne, Germany; 3 Laboratory of Phytopathology, Wageningen University & Research, Wageningen, The Netherlands; 4 Department of Crop Protection, Institute for Sustainable Agriculture (CSIC), Avenida Menéndez Pidal s/n, Campus ‘Alameda del Obispo’, Córdoba, Spain; 5 Provincial Key Laboratory of Agrobiology, Jiangsu Academy of Agricultural Sciences, Nanjing, China; 6 Theoretical Biology & Bioinformatics, Department of Biology, University of Utrecht, Utrecht, The Netherlands; 7 Molecular Plant Pathology, Swammerdam Institute for Life Sciences, Faculty of Science, University of Amsterdam, Amsterdam, The Netherlands; 8 Department of Biochemical Sciences, Sapienza University of Rome, Rome, Italy; 9 National Research Council of Italy, Institute of Molecular Biology and Pathology, Rome, Italy; 10 National Key Laboratory of Crop Genetic Improvement, Huazhong Agricultural University, Wuhan, Hubei China; 11 College of Agriculture, Shihezi University, Shihezi, Xinjiang China; 12 Present Address: Nanjing Engineering Research Center for Peanut Genetic Engineering Breeding and Industrialization, School of Food Science, Nanjing Xiaozhuang University, China, China; 13 Present Address: Department of Soil and Plant Microbiology, Zaidín Experimental Station, Granada, Spain

**Keywords:** Effectors in plant pathology, Pathogens, Fungal pathogenesis

## Abstract

Defoliating (D) strains of the vascular wilt fungus *Verticillium dahliae* cause severe yield losses in cotton and olive, but the genetic basis of this pathotype remained unknown. Using comparative genomics, functional genetics, structural analysis, and phylogenomics, we identify a D-pathotype–specific genomic region encoding two duplicated secreted effector genes. Simultaneous deletion of both copies abolishes pathogenicity and defoliation in cotton and olive, and affects virulence in *Nicotiana benthamiana* and *Arabidopsis thaliana*. Expression of the effector in non-defoliating strains induces cotton defoliation, and purified protein causes wilting and leaf drop. Structural analyses reveal a previously uncharacterized protein fold conserved across *Verticillium* and *Fusarium* species, with evidence of functional diversification and host specificity. Phylogenomic and genomic context analyses indicate repeated horizontal transfer mediated by giant transposable elements known as *Starships*. Together, these findings identify the D effector as a central determinant of defoliation and virulence and show how *Starship*-mediated gene transfer drives emergence of an agriculturally important fungal trait.

## Abstract (web summary)

Here the authors report a pair of identical effector genes in different fungal pathogens that cause vascular wilt diseases to cause defoliation in crops. Removing these genes stops disease, and mobile DNA elements appear to spread this virulence trait between fungal pathogens.

## Introduction

*Verticillium dahliae* is a soil-borne fungal pathogen that causes vascular wilt disease by colonizing the water-conducting xylem vessels of hundreds of dicotyledonous host plants, including cotton (*Gossypium hirsutum*), olive (*Olea europaea*), and tomato (*Solanum lycopersicum*)^1,2^. Infection typically starts once microsclerotia that reside in the soil germinate, triggered by root exudates, and the fungus penetrates the root. Subsequently, the fungus enters xylem vessels where further proliferation takes place, affecting water transport, and causing characteristic symptoms that include wilting, stunting, chlorosis, and early senescence^2,3^. Due to its broad host range, long-term prevalence of its resilient survival structures in the soil, limited curative treatments and scarcity of disease resistance in crop germplasm, Verticillium wilt control is notoriously difficult^2,4^

Although *V. dahliae* causes wilt disease in a broad range of host plants, virulence capacities and the severity of symptoms that are induced on host plants can vary considerably between individual strains. For instance, particular strains of *V. dahliae* can cause severe symptoms that include defoliation on cotton, olive, okra (*Hibiscus esculentus*) and pistachio (*Pistacia vera*), while other strains cause milder wilting symptoms and do not cause defoliation^5–8^. Consequently, on particular host plants, *V. dahliae* strains that show differential virulence capacities and disease symptoms are assigned to specific “pathotypes”^5,6^. Thus, *V. dahliae* strains that are highly virulent and cause rapid and severe defoliation on cotton, olive, okra, and pistachio are assigned the defoliating (D) pathotype, whereas strains that are moderately virulent and only induce mild wilting symptoms without defoliation are assigned to the non-defoliating (ND) pathotype^5^. The currently increasing prevalence of the highly virulent D pathotype strains poses a significant threat to cotton and olive plantations worldwide^9,10^. It is believed that D pathotype strains originated once in North America and subsequently spread to other continents through the dispersal of contaminated plant commodities^11,12^. Despite many efforts to develop molecular markers to robustly and accurately differentiate D and ND pathotype strains^13–15^, the molecular basis for the difference in aggressiveness between D and ND pathotype strains remains controversial so far.

To establish disease on their host plants, adapted pathogens secrete so-called effector molecules, many of which will modulate host physiology, including host immune responses, to promote successful infection^16,17^. However, for several pathogens, including *V. dahliae*, it was recently shown that particular effectors target host-associated microbiota to promote disease development^18–22^. Thus, it is well established that comprehensive identification of effector repertoires and determination of their modes of action is key to uncover virulence mechanisms and infection strategies of plant pathogens, which is ultimately required to design effective disease management strategies^23^. Here, we aimed to elucidate the molecular basis for the differential aggressiveness between *V. dahliae* strains that belong to the D and ND pathotype, respectively.

## Results

### Comparative genomics identifies D pathotype-specific effector genes

**Fig. 1:**
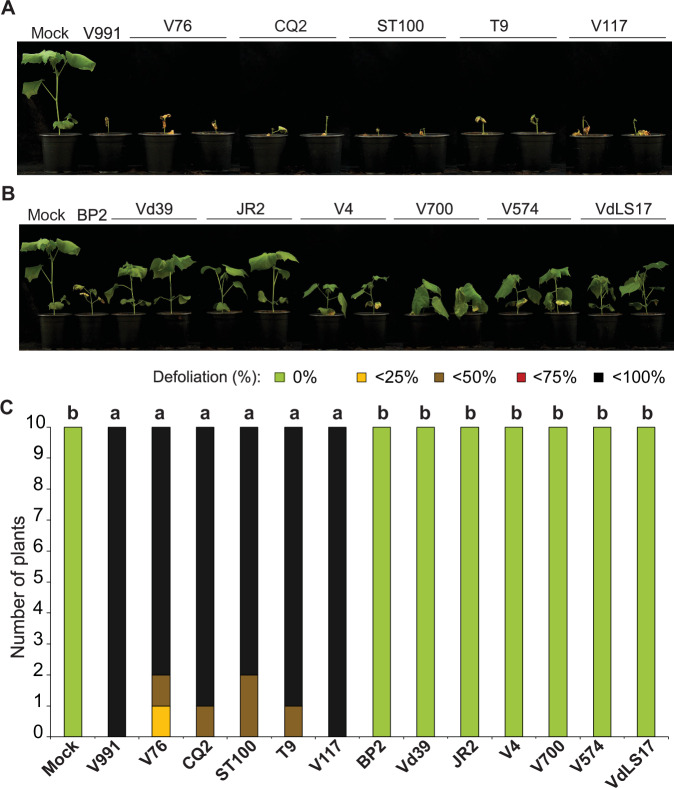
Phenotype of cotton plants inoculated with various sequenced *Verticillium dahliae* strains. **A** Typical phenotype of cotton plants (cv. Xinluzao63) upon mock-inoculation or inoculation with *V. dahliae* strains V991, V76, CQ2, ST100, T9 and V117 at 28 days post inoculation (dpi). Previously characterized D pathotype strain V991 used as inoculation control. **B** Typical phenotype of cotton plants upon mock-inoculation or inoculation with *V. dahliae* strains BP2, Vd39, JR2, V4, V574, V700, VdLS17 at 28 dpi. Previously characterized ND pathotype strain BP2 used as inoculation control. **C** Defoliation was classified as 0 (0% leaf drop off), 1 ( < 25% leaf drop off), 2 ( < 50% leaf drop off), 3 ( < 75% leaf drop off), and 4 ( < 100% leaf drop off) at 28 dpi. Lower case letters in C panel denotate significant grouping evaluated by one-way ANOVA followed by Tukey’s HSD post-hoc test (two-sided, adjusted for multiple comparisons within the family of pairwise contrasts; α = 0.05; *p* value = 2.13e–83). Inoculation experiments were performed with ten plants for each fungal strain and repeated twice independently with similar results.

A diverse set of *V. dahliae* strains had previously been sequenced, including well-characterized D pathotype strains (V991, T9, V117, and TM6) and ND pathotype strains (BP2, V4, cd3, and HN)^5,10,24–26^. To expand the set of strains for comparative genomics aimed at identifying determinants of defoliation, we generated a phylogenetic tree of in-house sequenced strains (Supplementary Table 1; Supplementary Fig. 1). This analysis identified six additional strains clustering with known D pathotype strains, while others grouped with ND strains. Four strains (V574, V700, V679, Vd39) clustered with D strains but were phylogenetically distinct, prompting disease assays to determine their pathotype. We also included presumed D strains (V76, V117, CQ2, ST100) and presumed ND strains (JR2, VdLs17). Disease assays confirmed V76, V117, CQ2, and ST100 as D pathotype strains, whereas JR2, VdLs17, V574, V700, and Vd39 were classified as ND (Fig. 1).

**Fig. 2:**
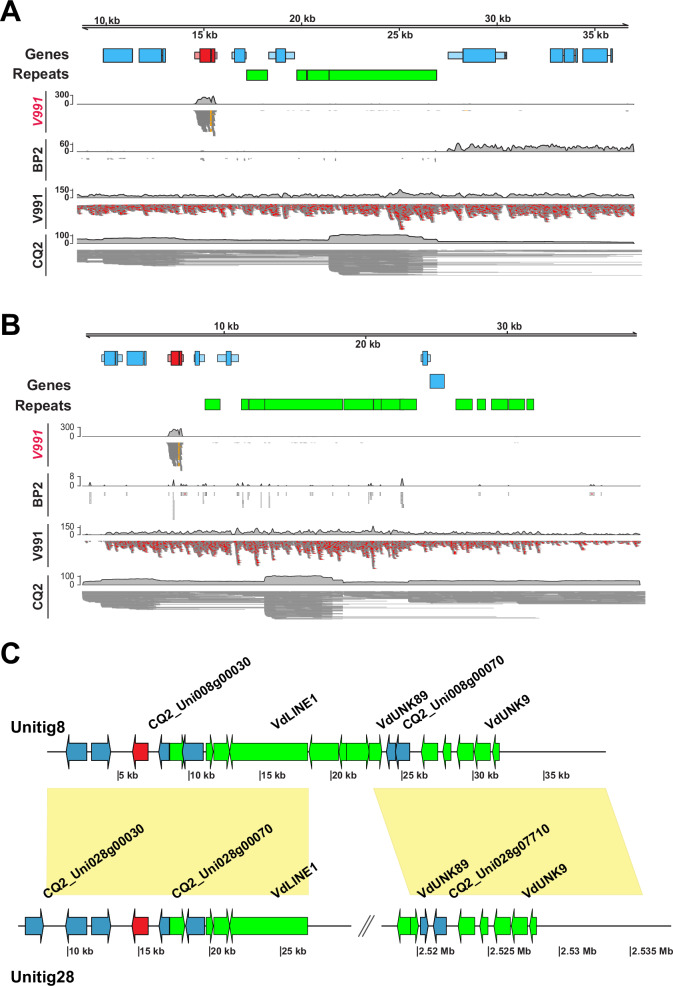
Two identical copies of effector gene generated by segmental duplication. **A**, **B** Schematic representation of genomic region (Unitig_28 and Unitig_8) where two candidate effector genes located. Gene models are shown in blue, the candidate effector genes are displayed in red, while repetitive elements are displayed in green. RNA reads from cotton plants infected by a D pathotype strain (V991 in red) that only mapped to candidate gene are showed in dark gray. Reads from DNA-seq of the ND pathotype strain BP2 and D pathotype strain V991 are mapped onto the CQ2 genome. The reads are shown as coverage plot and the depth of DNA reads coverage indicated in red. **C** Syntenic assignment of Unitig_28 and Unitig_8, the extensive synteny (yellow blocks indicate 100% sequence identity) points towards a segmental duplication. Gene models shown in blue, the candidate effector gene displayed in red, while repetitive elements displayed in green.

The genome of the D pathotype strain CQ2 was sequenced using PacBio (GCA_004798895.1)^27^ and used as the reference for comparative genomics with eight D pathotype strains (V991, T9, V117, TM6, V76, 463, CQ2, ST100) and nine ND pathotype strains (BP2, V4, HN, cd3, JR2, VdLs17, V574, V700, Vd39)^27,28^. This analysis identified ~200 kb of D-specific sequence harboring ~30 predicted protein-coding genes. To further refine the candidate region, genomes of 72 additional *V. dahliae* strains were analyzed (Supplementary Fig. 2). One strain, BGI_32, clustered outside both D and ND groups and was subsequently classified as ND based on disease assays (Supplementary Fig. 3), allowing the D-specific interval to be reduced to ~24 kb containing seven genes. InterProScan analysis^29^ showed that only two genes (CQ2_Uni028g00060 and CQ2_Uni008g00030) encode predicted secreted proteins and are thus candidate effectors. Transcriptome analysis of cotton plants inoculated with D pathotype strain V991 revealed high expression of both genes (Supplementary Fig. 4). Notably, the two genes are 100% identical despite residing on separate contigs (Unitig_28 and Unitig_8), and alignment of these contigs showed identical flanking regions, indicating segmental duplication (Fig. 2; Supplementary Fig. 4). These *in planta* expressed genes were tentatively named *D* genes due to their potential role in cotton defoliation.

### The D effector is responsible for cotton defoliation

**Fig. 3:**
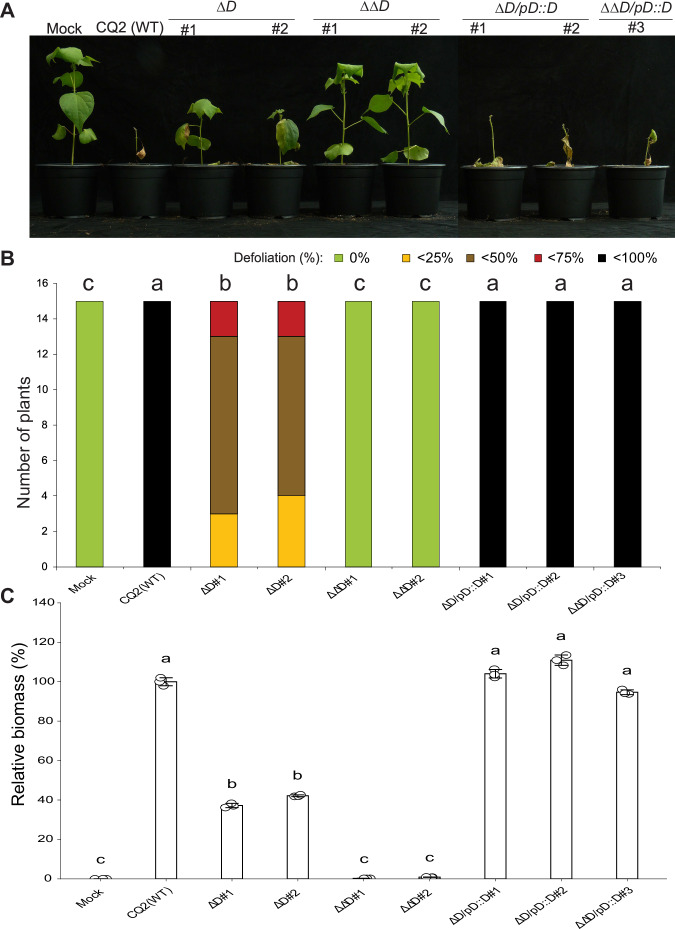
The D effector is responsible for cotton defoliation. **A** Typical phenotype of cotton (cv. Xinluzao63) upon mock-inoculation or inoculation with wild type strain CQ2 (WT), two Δ*D* mutants (Δ*D* #1 and Δ*D* #2), two ΔΔ*D* mutants (ΔΔ*D* #1 and ΔΔ*D* #2), two Δ*D* complementary strains (Δ*D*/p*D*::*D* #1 and Δ*D*/p*D*::*D* #2) and one ΔΔ*D* complementary strain (ΔΔ*D*/p*D*::*D* #3) at 28 days post inoculation (dpi). **B** Defoliation were classified as 0 (0% leaf drop off), 1 ( < 25% leaf drop off), 2 ( < 50% leaf drop off), 3 ( < 75% leaf drop off) and 4 ( < 100% leaf drop off). Lower case letters denote significant grouping evaluated by one-way ANOVA followed by Tukey’s HSD post hoc test (two-sided, adjusted for multiple comparisons within the family of pairwise contrasts; α = 0.05; *p* value = 5.84e–97). **C** Fungal biomass as determined with real-time PCR at 28 dpi. Bars represent *V. dahliae* ITS levels relative to cotton ubiquitin (*Gh*UB) levels (for equilibration) with standard deviation in a sample of five pooled plants. The fungal biomass in cotton plants upon inoculation with the wild type CQ2 is set to 100%. Lower case letters denote significant grouping evaluated by one-way ANOVA followed by Tukey’s HSD post-hoc test (two-sided, adjusted for multiple comparisons within the family of pairwise contrasts; α = 0.05; *p* value = <2e–16). Experiments were repeated twice independently with similar results.

To further investigate the role of the D effector in cotton pathogenicity, targeted deletion of the two *D* gene copies was performed in the D pathotype strain CQ2 by sequential homologous recombination (Supplementary Fig. 5A). Single-copy and double-copy deletion mutants (Δ*D* and ΔΔ*D*) were generated and verified by PCR (Supplementary Fig. 5B). Cotton seedlings were inoculated with wild-type CQ2, single deletion mutants (Δ*D*#1, Δ*D*#2), or double deletion mutants (ΔΔ*D*#1, ΔΔ*D*#2), and disease progression was monitored. Wild-type CQ2 induced severe defoliation at 28 days post inoculation (dpi) (Fig. 3A, B), whereas Δ*D* mutants showed significantly reduced aggressiveness and defoliation. In contrast, ΔΔ*D* mutants failed to cause disease, with no defoliation symptoms and no detectable fungal biomass by quantitative PCR, demonstrating that *D* is required for pathogenicity (Fig. 3A–C). Complementation of single and double deletion mutants with a genomic fragment containing the D coding sequence (*pD::D*) restored aggressiveness and defoliation (Fig. 3; Supplementary Fig. 6). Introduction of *pD::D* into the ND pathotype strain JR2 increased aggressiveness on cotton, causing severe stunting and defoliation (Supplementary Figs. 7, 8), confirming the role of D as a pathogenicity factor of D pathotype strains.

To determine whether the D effector protein alone can induce Verticillium wilt symptoms, cotton seedlings were treated with heterologously produced D protein at a low micromolar concentration (9 μM) and monitored over time, with water and the chitin-binding effector Vd2LysM (9 μM) as negative controls^30^. D protein treatment induced wilting, mild chlorosis, and initial cotyledon detachment after 10 days, progressing to severe chlorosis and detachment of more than half of the leaves (55 ± 6.45%) after 16 days (Supplementary Fig. 9). No symptoms were observed in control treatments, indicating that the D effector protein itself, rather than extensive fungal colonization, is sufficient to cause defoliation symptoms.

### The D effector mediates olive defoliation and pathogenicity on *Nicotiana benthamiana* and *Arabidopsis thaliana*

Since the D effector was shown to mediate cotton defoliation, we examined whether olive-defoliating *V. dahliae* strains carry the *D* gene. The *D* gene was detected in previously characterized D pathotype strains V150I, V641I, V356I, and V403II^31,32^, but not in the ND pathotype olive strain 812I^31^ (Supplementary Fig. 10). To test whether D mediates olive defoliation, both *D* gene copies were deleted in strain V150I (Supplementary Fig. 5C), and olive plants were inoculated with three double-deletion mutants (ΔΔ*D*#5, ΔΔ*D*#7, ΔΔ*D*#8) or wild-type V150I. While wild-type V150I caused severe disease, including defoliation and plant death, ΔΔ*D* mutants showed greatly reduced pathogenicity (Table 1, Supplementary Fig. 11), demonstrating that the D effector is required for olive defoliation.

**Table 1:** Bioassay of *D* gene deletion strains on olive (cv. Picual) plants

Treatments	Disease parameters				
	AUDPC^a^	Final DI (%)^b^	Final DII^c^	M (%)^d^	S^e^
V-150I (WT)	70.5a	44.4	0.33	11.1	1.31
*ΔΔD* #5	1.6b	22.2	0.02	0	0.08
*ΔΔD* #7	0b	0	0	0	0b
*ΔΔD* #8	0b	0	0	0	0b
Control	0b	0	0	0	0

Significant differences among isolates were detected (F = 3.11; *p* = 0.0164). When significant effects were identified, mean comparisons were performed using the Least Significant Difference (LSD) test for all pairwise comparisons at a significance level of α = 0.05. All statistical tests were two-sided, and no additional adjustments for multiple comparisons were applied beyond the inherent LSD procedure. Different letter labels indicate statistically significant differences.

^a^AUDPC, area under the disease progress curve over time up to 132 days post inoculation (dpi).

^b^Final DI, final disease incidence (%).

^c^Final DII, disease intensity index was calculated from data on incidence and severity of symptoms recorded at 132 (dpi).

^d^M, dead plants (%) at 132 (dpi).

^e^S, mean of disease severity symptoms at 132 (dpi). AUDPC data at 132 days were analyzed using analysis of variance (ANOVA) under a completely randomized design.

**Fig. 4:**
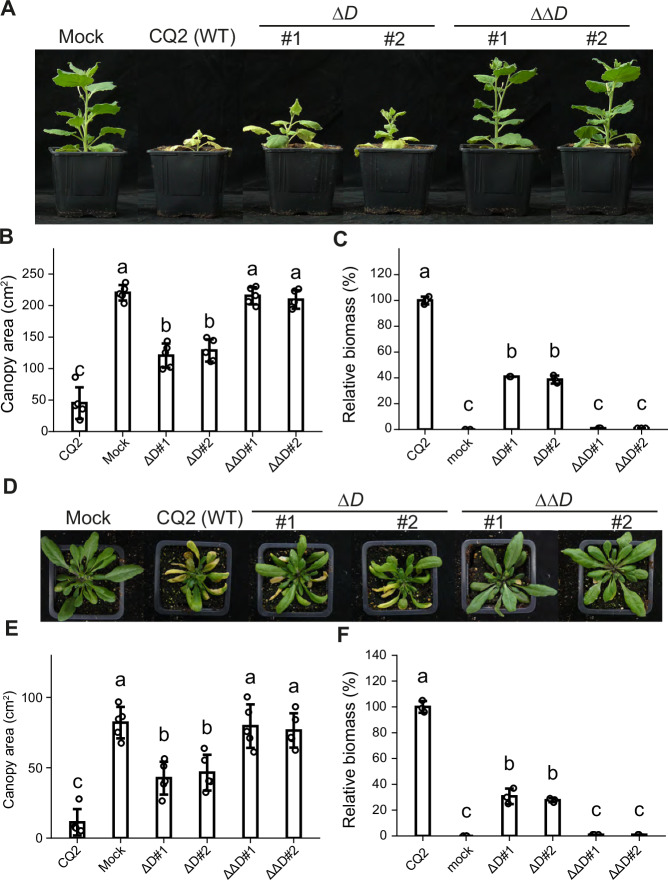
D effector is a pathogenicity factor on *Nicotiana benthamiana* and *Arabidopsis thaliana.* **A** Typical phenotype of *N. benthamiana* plants that were mock-inoculated or inoculated with wild type strain CQ2 (WT), two Δ*D* mutants (#1 and #2), two ΔΔ*D* mutants (#1 and #2) at 21 days post inoculation (dpi). **B** Quantification of the canopy area of *N. benthamiana* at 21 dpi. Bars represent the average canopy area of five plants with standard deviation. Lower case letters denotate significant grouping evaluated by one-way ANOVA followed by Tukey’s HSD post hoc test (two-sided, adjusted for multiple comparisons within the family of pairwise contrasts; α = 0.05; *p* value = 3.64e–14). **C** Fungal biomass as determined with real-time PCR at 21 dpi. Bars represent *V. dahliae* ITS levels relative to *Nb*RuBisCo (for equilibration) with standard deviation in a sample of five pooled plants while the fungal biomass in *N. benthamiana* plants upon inoculation with the wild type CQ2 is set to 100%. Lower case letters denote significant grouping evaluated by one-way ANOVA followed by Tukey’s HSD post-hoc test (two-sided, adjusted for multiple comparisons within the family of pairwise contrasts; α = 0.05; *p* value = 2e–16). **D** Typical phenotype of *A. thaliana* (Col-0) plants that were mock-inoculated or inoculated with indicated fungal strains in panel (**A**) at 21 dpi. **E** Quantification of the rosette area of five *A. thaliana* plants at 21 dpi. Bars represent the average rosette area of five plants with standard deviation. Lower case letters denote significant grouping evaluated by one-way ANOVA followed by Tukey’s HSD post hoc test (two-sided, adjusted for multiple comparisons within the family of pairwise contrasts; α = 0.05; *p* value = 1.1e–8). **F** Fungal biomass as determined with real-time PCR at 21 dpi. Bars represent *V. dahliae* ITS levels relative to *At*RuBisCo (for equilibration) with standard deviation in a sample of five pooled plants while the fungal biomass in *A. thaliana* plants upon inoculation with the wild type CQ2 is set to 100%. All *N. benthamiana* and *A. thaliana* inoculation experiments were performed with five plants for each fungal strain and repeated twice independently with similar results. Lower case letters denote significant grouping evaluated by one-way ANOVA followed by Tukey’s HSD post hoc test (two-sided, adjusted for multiple comparisons within the family of pairwise contrasts; α = 0.05; *p* value = 2e–16).

To determine whether the role of the D effector extends beyond cotton and olive, Δ*D* and ΔΔ*D* mutants were inoculated on *Nicotiana benthamiana* and *Arabidopsis thaliana*. On *N. benthamiana*, Δ*D* mutants showed reduced aggressiveness, with increased canopy area and lower fungal biomass, while ΔΔ*D* mutants were non-pathogenic, showing no disease symptoms or detectable fungal biomass (Fig. 4A–C). Similarly, on *A. thaliana*, Δ*D* mutants exhibited reduced aggressiveness, as indicated by larger rosette leaf area and lower fungal biomass, whereas ΔΔ*D* mutants were non-pathogenic (Fig. 4D–F). These results indicate that the D effector is required not only for defoliation on highly susceptible hosts but also for general pathogenicity on *N. benthamiana* and *A. thaliana*.

### D effector homologs occur across *V. dahliae* strains

**Fig. 5:**
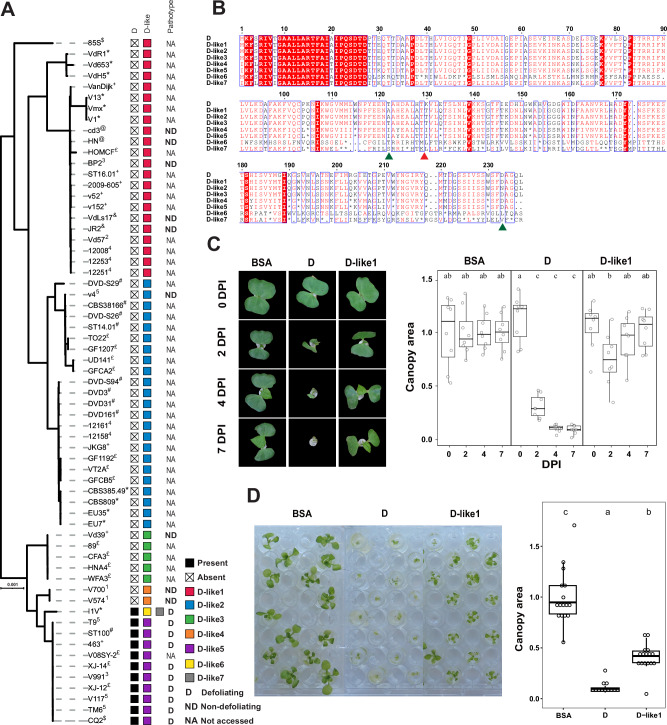
A multiallelic *D-like* gene occurs in the *V. dahliae* population. **A** Distribution of the *D* gene and *D-like* alleles across *V. dahliae* strains. Phylogenetic relationships among sequenced *V. dahliae* strains were determined based on whole genome alignments and branch lengths in the tree indicate sequence divergence. £= Chavarro-Carrero et al., 2021; * = Chavarro-Carrero et al., unpublished; @= Xu et al., 2012; $= Depotter et al., 2019; += Gibriel et al., 2019; &= Faino et al., 2015; #= de Jonge et al., 2012; 1= Milgroom et al., 2016; 2= Li, 2019; 3= Zhang et al., 2012; 4= Fan et al., 2018; 5= Keykhasaber, 2017. **B** Alignment of the predicted proteins encoded by the *D* gene and by each of the *D-like* alleles. The red arrowhead indicates the position at which the *D-like* 4 allele is disrupted by a retrotransposon insertion. The green arrowheads indicate the positions at which differences among the highly identical proteins encoded by the alleles *D-like* 1, *D-like* 2, and *D-like* 3 are observed. Asterisks indicate stop codons. The numbers on top of the alignment indicate amino acid positions in the sequences. **C** Typical appearance of cotton seedlings (cv. Xinluzao63) upon application of D and D-like1 effector proteins from *Verticillium dahliae* (5 µM), at 0, 2, 4, and 7 days post treatment. Bovine serum albumin (BSA; 5 µM) was used as control. Quantification of the canopy area of treatments shown is normalised by the average of BSA treated plants at 0, 2, 4, and 7 days after start of the treatment. Each boxplot represents eight plants with the center line indicating the median while the box outlines the 25th to 75th percentile. Lower case letters denote significant grouping evaluated by one-way ANOVA followed by Tukey’s HSD post-hoc test (two-sided, adjusted for multiple comparisons within the family of pairwise contrasts; α = 0.05; *p* value = 3.37e–24). Experiments were repeated twice with similar results. **D** Typical appearance of *Arabidopsis thaliana* seedlings (Col-0) upon application of *D* and *D-like1* effector proteins from *Verticillium dahliae* (5 µM), at two weeks after seed germination. Bovine serum albumin (BSA; 5 µM) was used as a control. Strong chlorosis and growth arrest are visible. Quantification of the canopy area of treatments shown is normalised by the average of BSA-treated plants at two weeks after the start of the treatment. Each boxplot represents sixteen plants with the center line indicating the median while the box outlines the 25th to 75th percentile. Lower case letters denote significant grouping evaluated by one-way ANOVA followed by Tukey’s HSD post-hoc test (two-sided, adjusted for multiple comparisons within the family of pairwise contrasts; α = 0.05; *p* value 9.7e–18). Experiments were repeated twice with similar results.

To assess the distribution and variability of the D effector in *V. dahliae*, 63 genome sequences were queried for the presence of the *D* gene (Supplementary Table 1). Interestingly, while all D pathotype strains carry D homologs that are 100% identical, diverged homologs occur throughout the *V. dahliae* strains encoding D homologs that display 91 to 82% amino acid identity, corresponding to seven *D*-*like* alleles (*D-like1* to *D-like7*) (Fig. 5A). *D-like1* was present in 22 strains, including reference genomes JR2 and VdLs17^33^; *D-like2* in 23 strains; *D-like3* in five strains; *D-like4* in two strains; *D-like5* in eleven strains; and *D-like6* and *D-like7* each in a single strain, I1V. *D-like1*, *D-like2*, and *D-like3* encode ~237 amino acid proteins ~80% identical to D and differ from each other by only one residue (Fig. 5B). In contrast, *D-like4*, *D-like5*, and *D-like7* encode truncated 82-amino-acid proteins due to premature stop codons, with *D-like4* additionally disrupted by a *Ty3*/*Gypsy*-like retrotransposon in V574 and V700 (Supplementary Fig. 12). *D-like6* co-occurs with *D* on the same contig in strain I1V.

To test functional activity, D-like1 protein was heterologously produced in *E. coli* and cotton seedlings were exposed to 5 μM D-like1, with bovine serum albumin (BSA) as negative control and D protein as a positive control. While D protein induced wilting, chlorosis, and >50% leaf detachment by 16 days (Fig. 5C), D-like1 caused no symptoms other than a slight and transient epinastic movement of the cotyledons at 2 dpi, indicating it cannot trigger cotton defoliation. *A. thaliana* seedlings grown on 5 μM D protein exhibited chlorosis and arrested growth at the two-leaf stage, whereas D-like1 also caused significant growth inhibition relative to BSA-treated controls (Fig. 5D). These results indicate that *D-like1* exerts differential effects depending on the plant species.

### *Starship* giant transposon-mediated horizontal transfer of the *D* gene

To determine whether D protein homologs occur beyond *V. dahliae*, ~22,000 fungal genomes from NCBI were queried, identifying 19 additional homologs (Supplementary Data 1). Within *Verticillium*, *D* homologs were detected in only 3 of the 10 *Verticillium* species, as they were additionally found in *V. alfalfae* and *V. longisporum*, but not in other species. *V. alfalfae* strains Ms102 and PD683 harbor a novel *D-like8*. *V. longisporum* strains showed complex patterns: vl1 has five copies (*D-like8*, *D-like10*, *D-like11*, and two *D-like9*), PD589 and VLB2 have three copies with two *D-like8* and differing third copies (*D-like12* in PD589, *D-like9* in VLB2), Vl43 contains *D-like8* and *D-like9*, and Vl32 carries *D-like1*.

Notably, outside *Verticillium*, *D* homologs were detected only in *Fusarium* species; two in *F. secorum* and 17 in ten *formae speciales* of *F. oxysporum*, with 80–90% identity to *V. dahliae* D (Supplementary Data 1). Most strains carried a single homolog, but some had multiple copies. *F. oxysporum* f. sp. *vasinfectum* (*Fov*) and f. sp. *melongenae* (*Fomel*) had two identical copies, one *Fov* strain had three identical copies plus a fourth variant, and f. sp. *melonis* (*Fom*) had the highest diversity with four *D-like* versions (*D-like13*, *D-like14*, *D-like15*, *D-like27*). *D-like13*, present in 30 of 48 *Fom* strains, encodes a 27-aa peptide disrupted by an inserted gene. *Fom* strains R12/13 and NRRL26174 carry both *D-like15* and *D-like27* (Supplementary Data 1).

The restricted occurrence of *D* homologs to *Verticillium* and *Fusarium*, which belong to the distant fungal orders Glomerellales and Hypocreales, suggests horizontal gene transfer (HGT). To test this, alien index^34^ (AI) scores were calculated for 12,882 Pezizomycotina genomes using non-*Verticillium* Glomerellales as ingroup and all other genera as outgroup. The *D* gene exhibited an AI score of 460.5, strongly supporting HGT across orders.

To investigate the divergence of *D* homologs, a phylogeny based on nucleotide sequences was inferred. *D* homologs from *Verticillium* formed a distinct clade from those in *Fusarium*, suggesting a single HGT event between the two genera (Supplementary Fig. 13A). To estimate its timing, synonymous substitution rates (*K*_*s*_) of the *D* gene were compared with the vertically inherited *GAPDH* gene. The *K*_*s*_ between the *V. dahliae D* gene and its closest *Fusarium* ortholog was lower than *GAPDH K*_*s*_ between *V. dahliae* and *Fusarium*, but higher than *GAPDH K*_*s*_ between *V. dahliae* and *V. alfalfae* (Supplementary Fig. 13B), indicating that the cross-order HGT occurred prior to *Verticillium* species diversification. Additionally, lower *K*_*s*_ values between *D* orthologs of *V. dahliae* and *V. alfalfae* relative to *GAPDH* suggest a more recent cross-species HGT within *Verticillium*. Together, these results indicate multiple horizontal transfers of *D*, including between *Verticillium* and *Fusarium* and among *Verticillium* species.

**Fig. 6:**
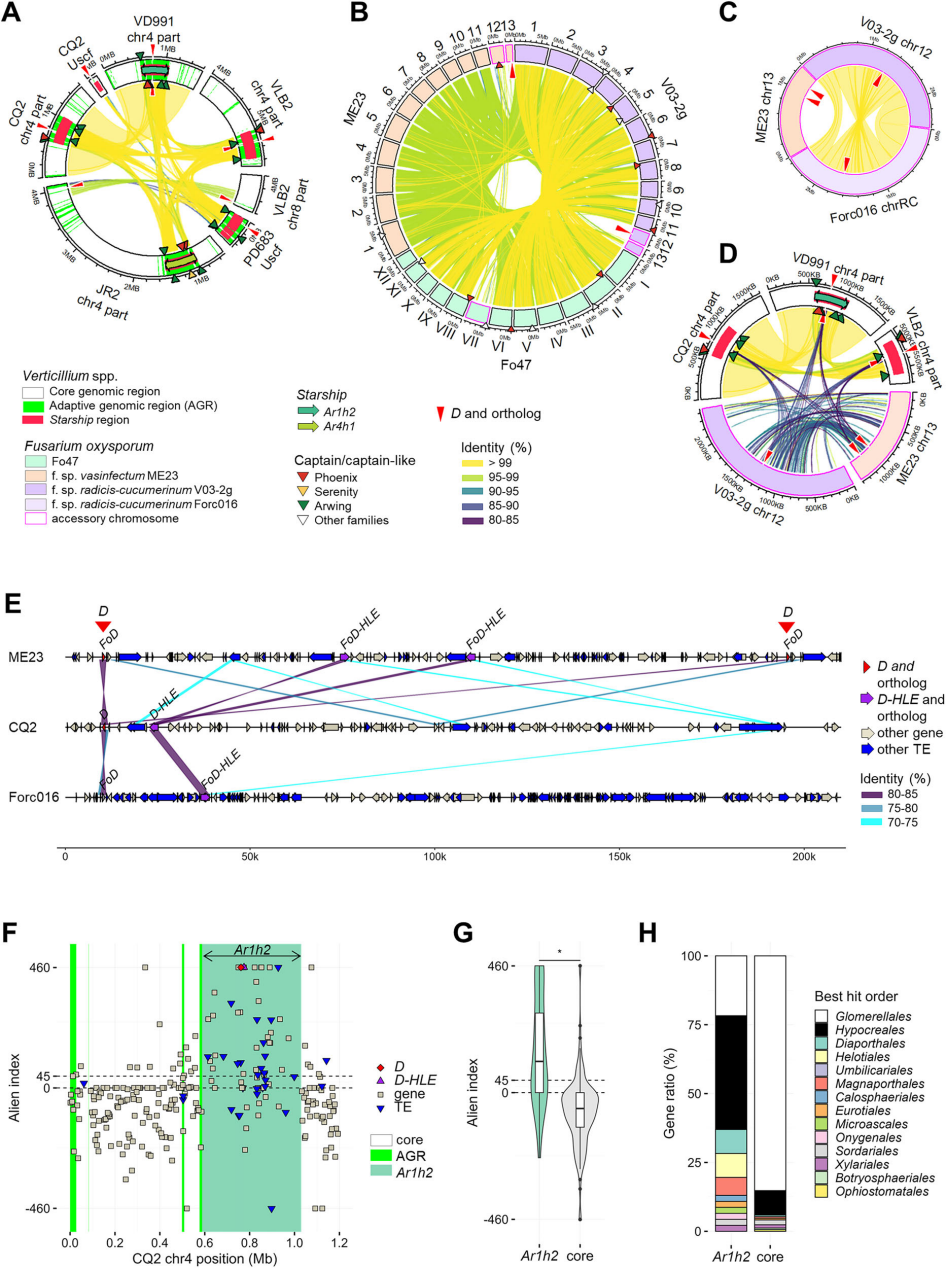
*Starship*-mediated *D* gene transfer and its association with a *Helitron*-like transposon. **A**–**D** Circular plots showing synteny among genomic regions of *Verticillium* and *Fusarium* strains. Red elongated arrowheads indicate positions of *D* homologs sharing >89% nucleotide sequence identity over 99% coverage. Tracks in *Verticillium* genomic regions are colored to indicate conserved core genomic regions or plastic adaptive genomic regions (AGRs) and are overlaid with bold lines and arrows representing *Starship* syntenic regions and *Starships*, with *Starships* colored by haplotype. AGRs exhibit chromatin profiles similar to those of *Starship* regions, and some AGRs may represent remnants of ancestral *Starships* (Cook et al., 2020; Sato et al., 2025). Regular triangles indicate captain and captain-like genes, colored by family. Ribbons connect homologous regions sharing >80% nucleotide sequence identity over 10 kb in (**A**–**C**) or over 1 kb in (**D**). **A** Synteny among *D*-carrying genomic regions in *Verticillium dahliae* strains CQ2, VD991, and JR2, *V. longisporum* strain VLB2, and *V. alfalfae* strain PD683. **B** Synteny among complete genome assemblies of *Fusarium oxysporum* strains Fo47, ME23, and V03-2g. **C** Synteny among *D*-carrying accessory chromosomes in *F. oxysporum* strains ME23, V03-2g, and Forc016. **D** Synteny among *D*-carrying genomic regions in *Verticillium* spp. and accessory chromosomes in *F. oxysporum*. **E** Location of *D* and *D-HLE* (*D-proximal Helitron-like Element*) in *V. dahliae* strain CQ2 and their orthologs (*FoD* and *FoD-HLE*) in *F. oxysporum* strains ME23 and Forc016. Ribbons connect homologous regions sharing >70% nucleotide sequence identity over 0.5 kb. **F** Genomic positions and inferred potential for cross-order horizontal gene transfer (HGT) of genes and transposable elements (TEs) surrounding the *D*-carrying *Ar1h2 Starship* in *V. dahliae* CQ2. Alien index (AI) values > 45 suggest HGT, whereas values < 0 suggest vertical inheritance. **G** Violin and box plots summarizing AI values of genes within the *Ar1h2 Starship* and flanking core genomic regions shown in (**F**). Black points indicate outliers. Center, upper, and lower lines in box plots denote the median, third quartile, and first quartile, respectively. Asterisks indicate a significant difference (two-sided Brunner-Munzel test, *p* < 2.2e–16, *n* = 46 in *Ar1h2* and *n* = 169 in core regions). **H** Stacked bar plots showing the percentage of *Verticillium* genes whose closest orthologs are found in genomes from the indicated orders of Pezizomycotina, excluding *Verticillium*, within the order Glomerellales. The query genes are the same as those analyzed in (**G**).

*Starships* are large DNA transposons carrying tens to hundreds of cargo genes and are widespread in Pezizomycotina fungi^35–37^. They have been implicated in HGT between fungal genera, including virulence genes between *Verticillium* and *Fusarium*^38,39^. To assess whether *D* homologs were transferred via *Starships*, previously identified *Starships* in *Verticillium* and *Fusarium* were analyzed^38,40^. In the *Verticillium* genus, the *D* gene is located on *Ar1h2* haplotype *Starships*, such as in *V. dahliae* strains CQ2, VD991, and JR2, but also *V. longisporum* strain VLB2, and *V. alfalfae* strain PD683 (Fig. 6A), whereas *D-like* orthologs are not. *Ar1h2 Starships* (~0.5 Mb) contain five tyrosine recombinase “captain” genes mediating *Starship* transposition, suggesting nested insertions. Notably, 74% of *Ar1h2* regions share >98% nucleotide identity with 64% of *Ar4h1 Starships*, which carry the gene encoding the horizontally transferred Av2 effector^38^, indicating divergence from a common ancestor. Furthermore, *Ar1h2 Starship* regions are >99% identical between *V. dahliae* and *V. alfalfae*, despite a genome-wide average identity of 93%, suggesting recent horizontal transfer between species. These findings implicate *Ar1h2 Starships* in the horizontal transfer of the *D* gene among *Verticillium* species.

In *Fusarium*, no *D* orthologs were found within previously identified *Starships*, nor were intact *Starships* or captain-like genes detected on chromosomes or scaffolds carrying *D*. However, in complete telomere-to-telomere assemblies of *F. oxysporum* f. sp. *vasinfectum* (strain ME23) and f. sp. *radicis-cucumerinum* (strain V03-2g)^41–43^, *D* orthologs reside on distinct accessory chromosomes (Fig. 6B), one of which closely resembles an accessory chromosome previously shown to transfer horizontally between *F. oxysporum* strains, including *F. oxysporum* f. sp. *radicis-cucumerinum* strain Forc016, and confer pathogenicity^44^ (Fig. 6C). Direct evidence of horizontal *Starship* transfer is difficult to obtain for ancient events, as *Starship* regions are highly plastic and may become unrecognizable due to rearrangements and active transposable elements (TEs)^38,45^. We therefore assessed whether *Ar1h2 Starships* share similarity with *Fusarium* regions surrounding *D*. While large-scale synteny is absent, partial sequence similarity exists at several loci, notably at the *D* locus and a nearby *Helitron*-like TE (*D-HLE*)~12 kb from *D*, with intervening regions populated by non-syntenic TEs (Fig. 6D, E). Helitrons transpose via rolling-circle replication and can capture adjacent sequences^46–48^. *D-HLE* is found exclusively in *Verticillium* and multiple Hypocreales genera, including *Fusarium*, and exhibits an AI score of 460.5, comparable to *D* itself, suggesting horizontal transfer (Supplementary Fig. 14). Thus, *D-HLE* could have mobilized *D* between an *Ar1h2 Starship* and an ancestral accessory chromosome.

To assess whether additional *Ar1h2* cargo genes were co-transferred, cross-order AI scores were calculated for all *Ar1h2* cargo elements and compared with flanking core genes. The median AI score of cargo genes (113) far exceeded that of flanking genes ( − 58), indicating horizontal transfer of most *Ar1h2* cargo genes (Fig. 6F, G). Among Pezizomycotina, 41% of the most conserved *Ar1h2* cargo orthologs are found in Hypocreales, including *Fusarium*, spanning nine additional genera (Fig. 6H, S14). Currently, these cargo genes are dispersed across chromosomes/scaffolds with frequent presence/absence variation (Supplementary Data 2), consistent with post-transfer *Starship* degradation. Collectively, these findings support a model in which the entire *Ar1h2 Starship*, rather than *D* and *D-HLE* alone, was horizontally transferred between *Verticillium* and Hypocreales, followed by *D-HLE*–mediated transposition of *D* and extensive post-transfer rearrangements that disrupted synteny at *D* loci between the genera.

### *Fusarium* D homologs induce wilting on cotton and cucurbit plants

**Fig. 7:**
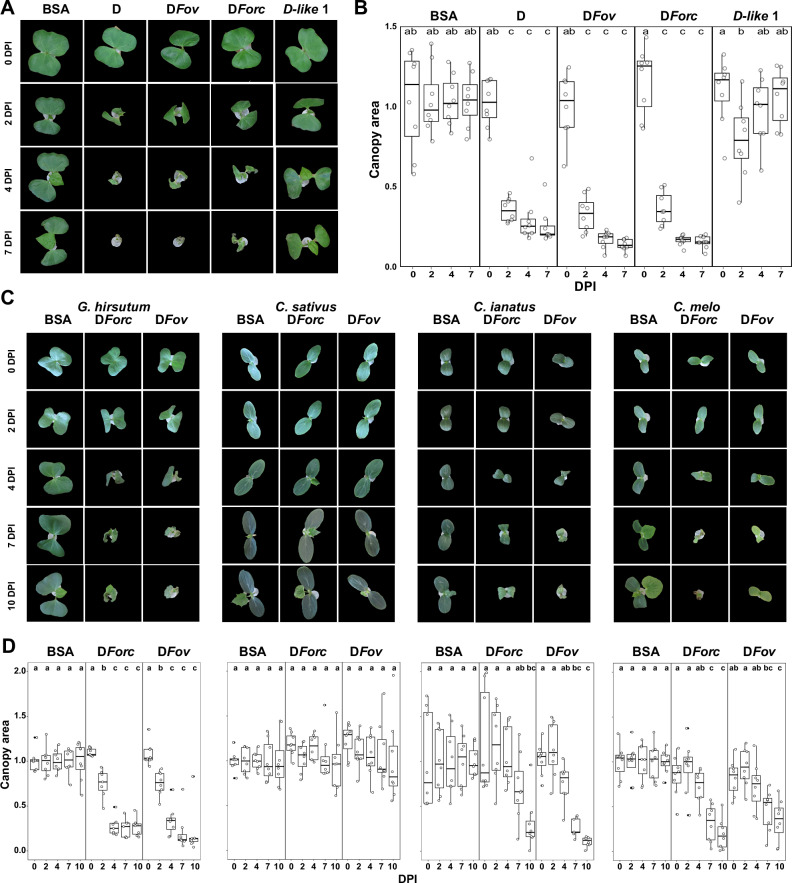
D homologs of *Fusarium oxysporum* differently affect cucurbit species. **A** Typical appearance of cotton seedlings (cv. Xinluzao63) upon application of D effector protein homologs from *Verticillium dahliae* (*D*, *D-like1*; 5 µM), *Fusarium oxysporum* f. sp. *vasinfectum* (D*Fov*; 5 µM), *F. oxysporum* f. sp. *radicis-cucumerinum* (D*Forc*; 5 µM) at 0, 2, 4 and 7 days post treatment. Bovine serum albumin (BSA; 5 µM) was used as control. **B** Quantification of the canopy area of treatments shown in panel (**A**) normalised by the average of BSA treated plants at 0, 2, 4, and 7 days after start of the treatment. Each boxplot represents eight plants with the center line indicating the median while the box outlines the 25th to 75th percentile. Lower case letters denote significant grouping evaluated by one-way ANOVA followed by Tukey’s HSD post-hoc test (two-sided, adjusted for multiple comparisons within the family of pairwise contrasts; α = 0.05; *p* value = 5.71e–70). Experiments were repeated twice with similar results. **C** Typical appearance of *G. hirsutum* (cotton, cv. Xinluzao63), *C. sativus* (cucumber, cv. Paraiso), *C. ianatus* (watermelon, cv. Black diamond) and *C. melo* (melon, cv. Brotmabon), upon application of (**D**) effector protein homologs from *Fusarium oxysporum* f. sp. *radicis-cucumerinum* (D*Forc*; 5 μM), *F. oxysporum* f. sp. *vasinfectum* (D*Fov*; 5 μM) at 0, 2, 4,7, and 10 days post treatment. Bovine serum albumin (BSA; 5 μM) was used as control. **D** Quantification of the canopy area of treatments shown in panel (**C**) normalised by the average of BSA treated plants at 0, 2, 4, 7, and 10 days after start of the treatment. Each boxplot represents eight plants with the center line indicating the median while the box outlines the 25th to 75th percentile. Lower case letters denotate significant grouping evaluated by one-way ANOVA followed by Tukey’s HSD post-hoc test (two-sided, adjusted for multiple comparisons within the family of pairwise contrasts; α = 0.05; from left to right: *p* value = 9.96e–37, *p* value = 0.6, *p* value = 1.88e–12, *p* value = 1.43e–18). Different letter labels indicate statistically significant differences (one-way ANOVA and Tukey test). Experiments were repeated twice with similar results.

To test whether *Fusarium* D effector homologs can induce cotton defoliation, proteins from the cotton-pathogenic *F. oxysporum* f. sp. *vasinfectum* (*DFov*, strain FovNRRL31665) and the cucurbit-pathogenic *F. oxysporum* f. sp. *radicis-cucumerinum* (*DForc*, strain Forc016, which cannot infect cotton) were produced and applied to cotton seedlings in MS medium at 5 μM, with *V. dahliae* D protein and BSA as positive and negative controls, respectively. Both *DFov* and *DForc* induced wilting within two days and cotyledon detachment after seven days, similar to the *V. dahliae* D protein, while BSA had no effect (Fig. 7; Supplementary Fig. 15). This demonstrates that *DFov* and *DForc* encode functional effectors capable of triggering cotton defoliation, even when produced by a fungus unable to infect cotton.

The effect of DFov and DForc was also tested on cucurbit hosts (cucumber, watermelon, and melon). Both proteins caused wilting in watermelon and melon within two days, followed by leaf shrinkage and curling after seven days, whereas cucumber plants remained unaffected (Fig. 7C, D). These results confirm that *Fusarium* D homologs are functional effectors capable of inducing host-specific disease symptoms despite sequence differences.

### The D effector defines a novel fungal effector family

To investigate the *in planta* function of D proteins, structural predictions for all functional D homologs were attempted using AlphaFold3^49^, but only low-confidence models were obtained (mean pLDDT <50). Consequently, experimental structure determination was pursued via vapor-diffusion crystallization. Circular dichroism (CD) spectroscopy indicated that the D protein is predominantly composed of β-sheets (Supplementary Fig. 16A), while size-exclusion chromatography (SEC) showed it exists as a monomer (Supplementary Fig. 16B). Crystallization trials of D homologs from *V. dahliae*, *F. oxysporum* f. sp. *vasinfectum* (D*Fov*), and *F. oxysporum* f. sp. *radicis-cucumerinum* yielded diffraction-quality crystals only for DFov.

**Fig. 8:**
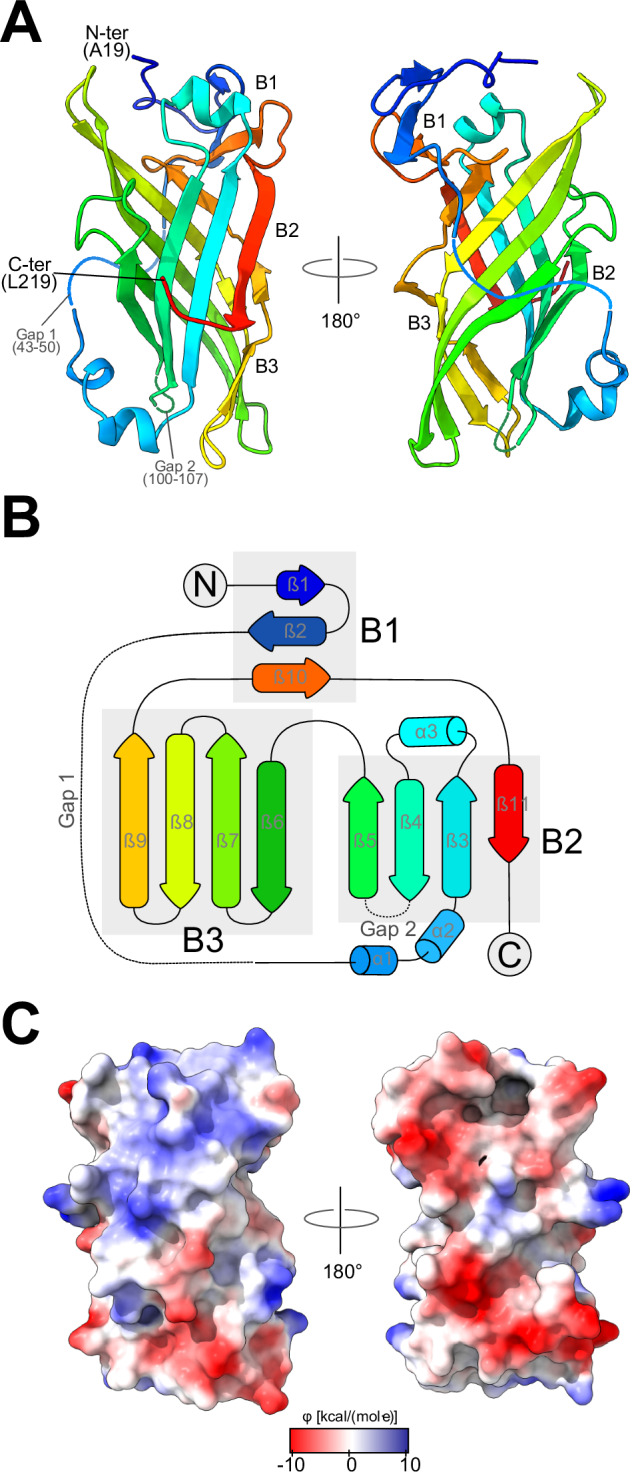
Crystal structure of the *F. oxysporum* f. sp. *vasinfectum* D protein homolog. **A** Cartoon representation of D*Fov* crystal structure colored according to the standard rainbow mode, with blue and red indicating the N-terminal and C-terminal regions, respectively. The first 19 N-terminal residues and 2 internal regions (indicated as dotted lines and named Gap 1 and 2) are not visible due to a high conformational mobility. **B** Fold topology of the different secondary structure elements of the D*Fov* fold. The three β-sheets are indicated as B1-3. B2 and B3 interact to form the β-barrel core of the protein. **C** Electrostatic surface potential of D*Fov* is calculated from atomic partial charges and coordinates according to Coulomb’s law: φ = Σ [q_i_ / (εd_i_)] using a default dielectric constant of 4.0 and a distance of 1.4 Å. Positive and negative regions are colored in blue and red, respectively. The surface of D*Fov* is anisotropically charged with a prevalence of positive or negative charge on opposite sides.

The D*Fov* structure was solved by X-ray crystallography at 1.5 Å resolution using single-wavelength anomalous diffraction (SAD) procedure with Hg atoms. The protein crystallized in space group P2₁ with one molecule per asymmetric unit and 33% solvent (Supplementary Table 2). Residues 1–18, 43–50, and 100–107 were unstructured and omitted. D*Fov* forms an elongated monomeric β-barrel composed of eleven β-strands organized into three antiparallel β-sheets (B1: β1β2β10, B2: β3β4β5β11, B3: β6β7β8β9) and three α-helices. B2 and B3 pack to form the β-barrel, B1 and α3 cap one end, while α1 and α2 connect β2 to β3 on the opposite side (Fig. 8A, B). The β-barrel is solvent-inaccessible, with a predominantly negative surface and a cluster of positive residues on one side (Fig. 8C).

Comparison with the low-confidence AlphaFold3 model using TM-align revealed poor structural similarity (template modeling (TM) score <0.5), confirming the model’s low accuracy (Supplementary Fig. 17). Structural homology searches via the Dali server^50^ identified a distant similarity to the *Parastagonospora nodorum* effector Tox3 (PDB: 6WES, Z-score 5.3)^51^. A detailed comparison of D*Fov* and Tox3 using TM-align yielded a TM-score of 0.50 and a root-mean-square deviation (RMSD) of 3.66, placing them at the borderline for fold-level similarity (Supplementary Fig. 18). A comprehensive structural similarity search with Foldseek against the AlphaFold database did not identify matches above this threshold, supporting the structural distinctiveness of DFov relative to currently predictable protein structures. Comparison against a dataset of ~5,000 AlphaFold2-predicted fungal effector structures^52^ identified several putative structural similarities (Z-scores up to 12.4) in *Claviceps purpurea*, *Epichloe festucae*, and *F. oxysporum* f. sp. *conglutinans* (Supplementary Data 3). However, these similarities remain within a borderline range of structural similarity. Consistently, the structural similarity network places D*Fov* in a cluster with these predicted effectors, suggesting that it adopts a fold with limited structural resemblance across some fungal effectors while remaining distinct from previously characterized structures (Supplementary Fig. 18). Together, these results support that DFov represents a previously uncharacterized effector fold.

## Discussion

The pathogenicity of plant-pathogenic fungi is mediated by secreted effector proteins that manipulate host physiology to promote infection^16,17,53–55^, although more recent evidence indicates that effectors can also target host-associated microbiota to promote infection^22^. Effector functions are often redundant, such that loss of individual effectors is typically dispensable for disease. Nevertheless, several studies demonstrate that a single effector can determine pathogenicity on specific hosts. For instance, the transcriptional activator-like effector PthA from *Xanthomonas citri* confers citrus pathogenicity when transferred into non-pathogenic strains^56–58^, and the necrotrophic effector SnTox1 enables *Parastagonospora nodorum* to infect wheat lines carrying the susceptibility gene *Snn1*^26,59^.

Although *Verticillium dahliae* strains collectively display a broad host range, pathogenicity and symptom severity vary widely^60^. Defoliating (D) and non-defoliating (ND) pathotypes have long been described in cotton- and olive-infecting strains^5,6^, yet the genetic basis of defoliation remained unclear. Although defoliation has been proposed to involve a horizontally transferred gene cluster from *Fusarium oxysporum* f. sp. *vasinfectum*, including secondary metabolite–associated genes, no single causal gene had been identified^61^. Here, we show that a single effector, termed the D effector, governs pathogenicity and defoliation in cotton and olive. Deletion of both *D* gene copies abolished defoliation in a dosage-dependent manner, whereas introduction into an ND strain conferred this trait. Moreover, importantly, application of the heterologously produced effector protein alone induced defoliation, demonstrating that the D effector directly drives symptom development.

*Verticillium dahliae* is regarded as an asexual species because no sexual cycle has been observed and mating type distribution is highly skewed, resulting in a predominantly clonal population structure^3,62–66^. This clonality was originally described using vegetative compatibility groups (VCGs)^67,68^, with isolates in clonal lineage VCG1A corresponding to the defoliating (D) pathotype, while non-defoliating (ND) strains occur across all other lineages^8,32,69,70^. The D pathotype is thought to have originated in North America before dispersing globally^71^. Consistent with this, SNP-based genotyping of a worldwide collection of *V. dahliae* isolates from diverse hosts showed that D pathotype strains from Europe, North America, and China all cluster within VCG1A and share nearly identical SNP haplotypes, supporting a single origin followed by intercontinental spread^11,65^.

Plant pathogens continuously reshape their effector repertoires to evade host immunity and sustain aggressiveness^16,55^. The emergence of new effector genes can enhance virulence or expand host range^72,73^ and is driven by mechanisms such as genome hybridization^74^, gene duplication^75^, and horizontal gene transfer^72,76^. *Verticillium dahliae* exhibits a two-speed genome in which segmental duplications generate adaptive regions enriched in *in planta*–expressed effector genes, implicating duplication as a major force in virulence evolution^77,78^. In this study, we show that the *D* effector gene arose by a recent segmental duplication, yielding two identical copies with highly similar flanking sequences. Quantitative phenotyping demonstrated that each copy contributes to fungal aggressiveness, indicating that duplication of the D effector is important for maintaining high virulence in D pathotype strains and pointing towards a dosage effect to establish the defoliation phenotype. More broadly, duplicated effector genes in filamentous pathogens often follow a duplication–divergence trajectory, in which redundancy allows one copy to evolve new functions, as observed for Crinkler effectors in *Phytophthora sojae*^79^ and effector diversification in *Ustilago maydis*^75^. Such processes can ultimately drive extensive effector expansions, exemplified by *Blumeria graminis*, where many effectors share an RNase-like fold derived from a single ancestral gene^80,81^. Our study revealed diverged D effector homologs throughout the *V. dahliae* strains, corresponding to seven D-like alleles (Fig. 5A). Interestingly, several of the variants appear pseudogenized due to premature stop codons or a retrotransposon insertion, suggesting that these variants have accumulated mutations such as premature stop codons or transposon insertions, which may reflect genome instability or relaxed selection, and could potentially influence recognition by host immune systems. Intriguingly, the D-like1 allele that encodes a full-length D effector variant does not cause symptom on cotton and cannot induce cotton defoliation, although growth inhibition on Arabidopsis could be observed. These findings suggest that D effector homologs have undergone functional diversification. However, the selective pressures driving their divergence—particularly into variants that differ in their ability to induce defoliation—remain unclear, as does the advantage of defoliation for *V. dahliae* on specific hosts. It is possible that defoliation enhances fungal dispersal or promotes tissue necrosis, thereby facilitating colonization. Future work should determine whether this diversification is linked to differences in host range, lifestyle, or other ecological factors among strains carrying distinct variants.

A key finding of this study is the identification of functional *D* effector homologs in multiple *formae speciales* of *Fusarium oxysporum*. Although *V. dahliae* is a broad host-range pathogen and *F. oxysporum* strains are host specialists, both share a similar infection strategy as soil-borne vascular pathogens and possess overlapping effector repertoires, including Ave1 and Av2^21,76,82^. Recent genomic analyses have implicated giant *Starship* transposons in the horizontal transfer of these virulence effectors^38^, and the D effector identified here represents an additional effector shared between the two genera. Our evolutionary analyses highlight *Starships* as major vectors of virulence gene transfer and reveal a previously unrecognized evolutionary link between *Starships* and pathogenicity-associated accessory chromosomes in *Fusarium*. The presence of nearly identical *D*-carrying *Starships* across *Verticillium* species supports recent *Starship*-mediated transfer within the genus, whereas sequence divergence patterns and *K*_*s*_ estimates indicate a more ancient transfer to or from *Fusarium*, where intact *Starships* have degenerated, but their cargo persists. In *Fusarium*, *D* orthologs reside on accessory chromosomes that are enriched in virulence genes and capable of horizontal chromosome transfer^83,84^. Similar patterns are observed for other *Starship* cargo, such as *spore killing* (*spok*) genes^85^, with *Starship* degradation attributed to genomic rearrangements, loss of captain genes, and repeat-induced point (RIP) mutation activity^36,37,45^. *Fusarium* accessory chromosomes are enriched in rearrangements, segmental duplications, and in transposon activity that may have contributed to the accelerated degeneration of *Starships*^86,87^. Our data further suggest that Helitron-like TEs facilitated movement of *Starship* cargo to and from accessory chromosomes, paralleling TE-mediated mobilization of effectors such as *ToxA* and *ToxB* in other fungi^88–90^. Together, these findings support a model in which coordinated interactions among *Starships*, TEs, and accessory chromosomes drive the spread and diversification of fungal virulence genes.

Interestingly, transcriptomic data show that the *D* gene is highly expressed early in infection (6 dpi; Fig. S4), well before visible defoliation symptoms appear. This timing suggests that the D protein may act in a preparatory role, priming host tissues for later disease progression. Alternatively, symptom development may require the protein to accumulate to a threshold level, or reflect delayed physiological responses initiated during early infection. Clarifying these possibilities will depend on identifying the molecular target(s) of the D protein, potentially aided by structural insights. However, despite advances in prediction tools such as AlphaFold3, no reliable structural model of the D effector is currently available. Determination of the 3D crystal structure of its *Fusarium* homolog (D*Fov*) revealed a previously uncharacterized protein fold with a weak fold-level similarity to SnTOX3 in the Protein Data Bank, placing the D effector in a distinct structural class and raising questions about its mechanism of action. Its activity across diverse hosts—cotton, olive, *Nicotiana benthamiana*, and *Arabidopsis thaliana*—suggests it targets a conserved molecular target involved in core immune or developmental processes and thus induces symptoms through general physiological effects. Interestingly, the D effector, also identified as the “TRADE” effector in *V. longisporum*, triggers bundle sheath cell identity switches into tracheary elements in *A. thaliana*, enhancing water storage and drought resilience, likely via interaction with VARICOSE (VCS), a conserved mRNA turnover component^91^. Whether this mechanism underlies cotton and olive defoliation remains to be confirmed.

Despite the severe impact of D pathotype strains and the occurrence of *D-like* genes in all thus far characterised *V. dahliae* strains, no resistance (*R*) genes have been identified in cotton or olive^92,93^. Because effectors can reveal host recognition, they are widely used as probes to identify *R* genes through hypersensitive responses in germplasm screening^94–97^. Accordingly, the D effector represents a promising tool for discovering *R* gene–mediated resistance in cotton, olive, and other crops.

## Methods

### Genome sequencing and deep transcriptome sequencing

The genome of *V. dahliae* strain CQ2 (Supplementary Table 1) was sequenced using PacBio SMRT long-read technology on the PacBio RSII platform, with library construction as previously described^27,28,33^. *V. dahliae* strains Vd39, V4, BP2, V574, V700, V117, V76, ST.100, T9, and V991 (Supplementary Table 1) were sequenced on the Illumina HiSeq 2000 following library preparation with 500-bp inserts and 100-bp paired-end reads at the Beijing Genome Institute (BGI, China).

Phylogenetic relationships among *V. dahliae* strains were inferred using REALPHY v1.12^98^, with genomic reads mapped to the gapless reference genome of strain JR2^33^ using Bowtie2^99^. A maximum-likelihood tree was constructed with RAxML v8.2.8 under the GTRGAMMA model and 500 bootstrap replicates^100^, and rooted with *V. alfalfae* Ms102.

For transcriptome analysis, 12-day-old cotton seedlings were root-inoculated with conidiospores of the D pathotype strain V991 as previously described^101^. Stems were harvested at 6, 9, 12, and 15 days post inoculation for total RNA extraction using the Spectrum Plant Total RNA Kit (Sigma-Aldrich, USA). cDNA synthesis, library preparation with 200-bp inserts, and Illumina sequencing (90-bp paired-end reads) were performed at BGI (Shenzhen, China).

### *V. dahliae* comparative genomics

The *V. dahliae* strain CQ2 genome was assembled with HGAP v3.0 using default parameters^102^ and annotated with MAKER2 v2^103^, followed by manual curation of gene models in regions of interest, including verification of MAKER2 predictions and refinement of exon–intron boundaries using transcriptome data. To identify genes associated with cotton defoliation, Illumina reads from defoliating (D) and non-defoliating (ND) pathotype strains (Supplementary Table 1), together with sequence data from 72 *V. dahliae* strains from NCBI (project PRJNA171348), were mapped to the CQ2 genome using BWA with default settings^104^. Presence/absence analysis was performed with BEDtools^105^ and R^106^ by partitioning the genome into 100-bp bins and assessing read presence in 50-bp sliding windows, identifying regions covered by D strains but lacking ND strain coverage. Coverage was normalized as (reads per bin × 10,000,000) / total mapped reads, evaluated per sample, and filtered using a cutoff of 5. Genes located in lineage-specific (LS) regions unique to D pathotype strains were extracted using BEDtools intersect v2.25^105^, and bioinformatic predictions in these LS regions were validated by mapping RNA-seq reads from cotton plants infected with D pathotype strain V991 using TopHat v1.4.0^107^.

### Gene expression analysis

To assess *D* gene expression in *D* deletion and complementation strains, *V. dahliae* cultures were grown in liquid Czapek–Dox medium at 22 °C for one week with shaking at 150 rpm, after which mycelia and conidia were harvested for RNA extraction. First-strand cDNA was synthesized using the MMLV reverse transcriptase system (Promega, USA). RT-PCR was performed with primers CQ2D-F and CQ2D-R (Supplementary Data 4) in 25 μl reactions containing 17.9 μl sterile water, 5 μl 5× PCR buffer, 0.5 μl dNTPs, 0.5 μl of each primer, 0.1 μl GoTaq DNA polymerase (Promega, USA), and 1.0 μl first-strand cDNA (100 ng μl⁻¹). The *V. dahliae GAPDH* gene served as an endogenous loading control, and PCR products were analyzed by agarose gel electrophoresis.

### BLAST searches and protein sequence alignment

The genomes of 63 sequenced *V. dahliae* strains (Supplementary Table 1) were screened for *D*-*like* alleles using BLASTn with default parameters^108^. Matching sequences were extracted with the BEDtools getfasta utility^105^ and aligned using webPRANK^109^ to assess allelic variation. Predicted amino acid sequences encoded by the *D* gene and its *D*-*like* alleles were also aligned with webPRANK^109^ and visualized using ESPript v3.0^110^.

### Heterologous D effector protein production and cotton bioassays

The D effector protein was produced in *Pichia pastoris* as previously described^30,111^. The *D* gene was PCR-amplified with primers adding N-terminal His and FLAG tags (Supplementary Data 4) and cloned into the *P. pastoris* expression vector pPIC9 (Invitrogen, Carlsbad, CA, USA), which was transformed into strain GS115. Putative transformants were screened for D protein expression in BMM medium, and one clone was selected for large-scale cultivation in a BioFlo 120 fermenter (Eppendorf AG, Hamburg, Germany). The His-tagged D protein was purified using a Ni²⁺-NTA Superflow column (Qiagen, Venlo, the Netherlands), dialyzed against 100 mM NaCl, quantified by absorbance at 280 nm, and analyzed by SDS–PAGE and Western blotting with a mouse anti-His monoclonal antibody.

To evaluate the effect of the D effector, three-week-old cotton seedlings were uprooted, rinsed, and their roots immersed in 10 mL of D protein solution (8.96 μM). Water and the *V. dahliae* chitin-binding effector Vd2LysM (8.96 μM) were used as negative controls. Seedlings were incubated in a growth chamber at 21/19 °C (16/8 h day/night), 70% relative humidity, for 16 days and monitored for symptom development.

### D homolog identification

Genomes of approximately 22,000 fungi were downloaded from NCBI, and the *D* gene sequence from *V. dahliae* strain CQ2 was used to query these genomes with Exonerate v2.3.0^112^ using the protein2genome model and a 50% identity threshold. Exonerate output gff files were subsequently processed with gffread v0.11.7^113^ together with the corresponding genome sequences to extract predicted protein sequences (Supplementary Data 1).

### Phylogenetic analysis

Nucleotide sequences of *D* homologs were extracted from genome assemblies listed in Supplementary Data 1 using SeqKit v2.10.0^114^, with assemblies downloaded from NCBI via NCBI Datasets v18.6.0^115^. Sequences were aligned using the L-INS-i algorithm in MAFFT v7.526 with the *adjustdirection* option^116^, and ambiguously aligned sites were removed with TrimAl v1.5.rev0 using the *-automated1* option, retaining 740 aligned residues^117^. Phylogenetic inference was performed with IQ-TREE v3.0.1^118^, which automatically selected the best-fit nucleotide substitution model (TPM2u+F + G4) using ModelFinder^119^, and branch support was estimated with 1000 ultrafast bootstrap replicates^120^. The consensus tree was visualized using the R package *ggtree* v3.14.0^121^, and the closest *Fusarium* ortholog to D was identified using the *cophenetic.phylo* function in the R package *ape* v5.8.1^122^. Synonymous substitution rates (Ks) were calculated with KaKs_Calculator v2.0 using the MA model^123^ on coding sequences of D and GAPDH homologs extracted from coordinates listed in Supplementary Table 3.

### Detection and visualization of genomic syntenies

Accession numbers of genome assemblies used for synteny detection are listed in Supplementary Table 4. Genes and TEs of *V. dahliae* strain CQ2 were annotated as mentioned above. Genes of *F. oxysporum* strains ME23 and Forc16 were annotated in previous studies^41,43,124^, while TEs in these strains were annotated in this study using EDTA v2.2.1 with the options “--overwrite 1 --sensitive 1 --anno 1”^125^. *Verticillium* genomic regions (core regions, AGRs, and *Starship* regions) and synteny between *Verticillium* genomes were identified previously^38^. Synteny between *Fusarium* genomes and between *Verticillium* and *Fusarium* genomes was detected by pairwise genome sequence alignment using “nucmer” with the “--maxmatch” option, followed by alignment filtering using delta-filter with the ‘-m’ option and coordinate extraction using ‘show-coords’ with the “-THrcl” option in MUMmer v4.0.1^126^. The genomic synteny was visualized with R package ‘circlize’ v0.4.16^127^. Local sequence similarity was detected by pairwise genome sequence alignment using BLASTn with the same options mentioned above and visualized together with gene and TE loci using R package ‘gggenomes’ v1.0.1^128^.

### Identification of *Starships* and captain genes

*Starships* and captain-like genes in *Verticillium* shown in the figures were identified previously^38^. *Starships* reported from diverse Pezizomycotina fungi^38,40^ were screened for *D* homologs using BLASTn with the parameters described above, which identified no *Fusarium Starships* carrying *D*. Because additional genome assemblies have since become available, all currently available *Fusarium* assemblies in NCBI that contain *D* homologs (Supplementary Data 1) were further examined for *Starships* and captain-like genes. *Starship* detection was performed using the ‘starfish*’* v1.1.0 pipeline with previously described settings, which identifies insertion–deletion polymorphisms associated with 5′-end captain genes^40^. Captain-like genes were detected using a Pezizomycotina captain and captain-like protein database via translated nucleotide similarity searches implemented in MetaEuk v6.a5d39d9^129^. No captain candidates were identified on *D*-containing chromosomes or scaffolds (Supplementary Data 5), indicating the absence of detectable *Starships*.

### Disease assays

Cotton (*Gossypium hirsutum* cv. Xinluzao63), *Nicotiana benthamiana*, and *Arabidopsis thaliana* (Col-0) were grown under controlled greenhouse conditions (Unifarm, Wageningen, the Netherlands). Olive (*Olea europaea* cv. Picual) was grown in a greenhouse under natural lighting and day/night temperature of 27/21 °C (Córdoba, Spain). Two-week-old cotton and *A. thaliana*, three-week-old *N. benthamiana* and eight-month-old olive plants were used for inoculation. *Verticillium dahliae* strains (Supplementary Table 1) were grown on potato dextrose agar (PDA) at 22 °C for 7-10 days. Conidiospores were collected from PDA and washed with tap water for inoculation. Disease assays on cotton, *N. benthamiana* and *A. thaliana* were performed using the root-dip method as previously described^130,131^. Olive assays followed published protocols^132,133^. Symptoms were scored up to 21 (*A. thaliana* and *N. benthamiana*), 28 (cotton), or 132 (olive) days post inoculation (dpi). *A. thaliana* and *N. benthamiana* were photographed, and canopy area (*N. benthamiana*) and total rosette area (*A. thaliana*) were quantified with ImageJ. Cotton height was measured with a rectilinear scale, and defoliation was classified as 0 (0%), 1 ( < 25%), 2 ( < 50%), 3 ( < 75%), or 4 ( < 100%) leaf drop^26,134^. Fungal biomass in *N. benthamiana*, *A. thaliana*, and cotton was quantified by real-time PCR^130,131^. Olive disease severity was assessed twice weekly based on the percentage of leaves with chlorosis or defoliation: 0 (no symptoms), 1 (1–33%), 2 (34–66%), 3 (67–100%) and 4 (dead plant)^31,133^. Disease incidence (DI), mortality (M), and disease intensity index (DII) were calculated per treatment. DII was defined as DII = (ΣSi × Ni)/(4 × Nt), where Si = symptom severity, Ni = number of plants with severity Si, and Nt = total plants. Final DI was the percentage of affected plants at the end of the bioassay. The area under the disease progress curve (AUDPC) of DII over time^135^ and final severity were calculated. Data were analyzed by ANOVA, and means were compared using Fisher’s protected LSD (*P* = 0.05) in Statistix v10.0 (Analytical Software, 1985–2013).

### Generation of *D* gene deletion strains

To generate a *D* single gene deletion construct, sequence stretches of approximately 1.2 kb upstream and 1.3 kb downstream of the D coding sequence were amplified from genomic DNA of D pathotype strain CQ2, using primer pairs SKO-D-LBF/LBR and SKO-D-RBF/RBR (Supplementary Data 4). The amplicons were cloned into vector pRF-HU2 as described previously^136^, and the resulting deletion construct was transformed into CQ2 via *Agrobacterium tumefaciens*-mediated transformation^137^. Putative deletion transformants were selected on PDA containing 200 μg/mL cefotaxime and 50 μg/mL hygromycin B (Duchefa, Haarlem, The Netherlands) and homologous gene replacement was verified with PCR analysis using outside primer-F and outside primer-R (Supplementary Data 4). To generate *D* double gene deletion mutants, sequence stretches of approximately 1.1 kb upstream and 1.2 kb downstream of the *D* coding sequence were amplified using primer pairs DKO-D-LBF/LBR and DKO-D-RBF/RBR (Supplementary Data 4). The amplified products were cloned into vector pRF-NU2. Next, the gene replacement construct was transformed into a *D* gene single deletion mutant. Putative double deletion transformants were selected on PDA containing 50 μg/mL hygromycin B and 15 μg/mL nourseothricin (Sigma-Aldrich Chemie GmbH, Buchs, Switzerland) and were subjected to PCR to confirm genuine double *D* gene deletions using outside primer-F and outside primer-R (Supplementary Data 4). Reverse transcription-PCR (RT-PCR) was used to confirm no *D* gene transcripts in *D* double deletion mutants using primers D-RT-F and D-RT-R, and *V. dahliae* GAPDH (glyceraldehyde-3-phosphate dehydrogenase) gene as an endogenous control (Supplementary Data 4). Meanwhile, the same *D* single deletion construct and double deletion constructs were used to generate *D* single and *D* double deletion mutants in *V. dahliae* strain V150I, which causes defoliation on olive^32^. *D* gene complementation was performed by using a genomic construct consisting of the *D* gene coding sequence with 1.1 kb upstream and 1.2 kb downstream sequences (pD::D) using primer pairs D-com-F and D-com-R (Supplementary Data 4). The amplified products were cloned into GatewayTM compatible vector PCG using a standard BP reaction. The gene complementation construct was further transformed into a *D* single deletion mutant, a *D* double deletion mutant, and an ND pathotype strain JR2, respectively. Putative *D* gene complementation transformants were selected on PDA supplemented with 200 μg/mL of cefotaxime and 25 μg/mL geneticin (Sigma-Aldrich Chemie BV, Zwijndrecht, The Netherlands). Reverse transcription-PCR (RT-PCR) was used to examine *D* gene transcription in these transformants.

### Heterologous production of D homologs in *Escherichia coli*

The nucleotide sequences encoding mature D protein from *Verticillium dahliae* strain CQ2, D-like 1 from *V. dahliae* strain JR2 and D homologs from *F. oxysporum* f. sp. *vasinfectum* strain *Fov*NRRL31665 (D*Fov*) and from *F. oxysporum* f. sp. *radicis-cucumerinum* strain *Forc*016 (D*Forc*) without predicted signal peptides were synthesised by Eurofins Genomics (Louisville, KY, USA) and amplified with PCR (Supplementary Data 4). The resulting amplicons were cloned into the expression vector pET-28a (Novagen, Madison, USA), sequenced, and transformed into chemo-competent *E. coli* strain BL21 cells. Single colonies were picked from selection medium and grown overnight in LB (10 g/L tryptone, 5 g/L yeast extract, 5 g/L NaCl) supplemented with 50 µg/mL kanamycin at 37 °C and 200 rpm. The optical density (OD_600_) of the overnight cultures was determined spectrophotometrically (BioPhotometer, Eppendorf, Hamburg, Germany). Next, 1 L cultures in LB medium were initiated at an OD_600_ of 0.1 by adding an appropriate volume of overnight culture and subsequently incubated at 37 °C and 200 rpm until an OD_600_ of ~0.6 was reached. Then, protein expression was induced by addition of 100 µL of a 1 M aqueous solution of IPTG (Duchefa, Haarlem, the Netherlands) to reach a final concentration of 100 µM, and cultures were incubated overnight at 37 °C and 200 rpm. Subsequently, cultures were pelleted for 30 min at 5000 × *g* at room temperature. Next, pelleted cells were resuspended in lysis buffer (50 mM Tris, 200 mM NaCl, 10% [v:v] glycerol, 6 mg/mL lysozyme, 2 mg/mL sodium deoxycholate, 20 μg/mL DNase, 1 mM PMSF; pH 8.0) and lysed for one hour on ice. The lysate was then centrifuged for 30 minutes at 21,000 x *g* at 4 °C. The supernatant was transferred to a new tube and subsequently loaded onto an affinity purification column (His60 Ni Superflow, TaKaRa, Gotenburg, Sweden) that was first equilibrated with equilibration buffer (20 mM tris, 200 mM NaCl, 30 mM Imidazole; pH 8.0). Next, the column was washed with 20 volumes of equilibration buffer, followed by protein elution using a gradient of equilibration and elution buffer (20 mM Tris, 200 mM NaCl, 500 mM Imidazole; pH 8.0). Protein fractions were analysed on SDS-PAGE gels (Bio-Rad, California, USA) for purity and abundance. Suitable fractions were pooled and dialyzed against low salt solution (25 mM tris, 25 mM NaCl; pH 8.0) overnight at 4 °C. Final protein preparations were run on SDS-PAGE to assess purity (Supplementary Fig. 18) and concentrations were determined using the Bradford method^138^.

### Bioassays on *Arabidopsis thaliana* and cotton seedlings

To assess the effect of treatment with a D effector protein and a *D-like1* protein from *Verticillium dahliae*, bioassays were conducted on *Arabidopsis thaliana* and cotton seedlings. For the *Arabidopsis thaliana* (Columbia-0) bioassay, ethanol-sterilized seeds were placed in 96-well plates with semi-solid media (½ MS, 0.2% agar, 0.5% sucrose) supplemented with the protein homologs to a final concentration of 0.2 mg/mL Bovine Serum Albumin (BSA) at the same concentration and the dialysis buffer alone (2.8 mM K₂HPO₄, 2.1 mM KH₂PO₄, 200 mM NaCl, pH 7.1) served as negative controls. The seedlings were stratified at 4 °C in the dark for 24 hours and then incubated in a growth chamber at 22 °C with a 16/8 h day/night cycle for 14 days. For the cotton bioassay, one-week-old seedlings were uprooted, and the roots were placed into 10 mL protein solutions (5 µM). BSA (5 µM) was used as negative control, and the *V. dahliae* D effector protein (5 µM) was used as a positive control. The cotton seedlings were incubated in a growth chamber for 10 days at 22 °C/19 °C (day/night) with 70% relative humidity. In both experiments, plants were regularly monitored for phenotypic differences. At the end of the treatments, plants were photographed, and ImageJ was used to determine the canopy area (leaf area in cotton was measured at 2, 4, 7, and 10 days post-treatment). The data were analysed using an analysis of variance (ANOVA) combined with Tukey’s HSD (Honestly Significant Difference) test to identify significant differences.

### Calculation of alien indexes

Prior to calculating the alien index of genes in *Verticillium dahliae* strain CQ2, the gene models were improved from a previous annotation^38^, which was based solely on coding sequence prediction using “pipeline C” of BRAKER version 3.0.8^139^ and failed to annotate the *D* gene. In this study, genes in the chromosomal-level assembly of CQ2^28^, in which repetitive elements were previously soft-masked^38^, were re-predicted using ‘pipeline D’ of the same BRAKER version with options “--gff3” and “--fungus”, incorporating expression evidence from RNA-Seq data deposited in the NCBI Short Read Archive (accession numbers: SRR13442519, SRR13442520, and SRR13442521) that were generated in the previous study^140^. In addition, gene annotations from *V. dahliae* strain JR2 (accession number VDAG_JR2v.4.0 in EnsemblFungi)^33^ were transferred to CQ2 using GeMoMa version 1.9^141,142^, combined with a translated nucleotide-to-nucleotide search performed with MMseqs2 version 18.8cc5c^143^. All annotation datasets were then combined in a non-redundant manner using GffCompare version 0.12.10^113^, with the following priority order: BRAKER “pipeline D” annotations, JR2-based ortholog annotations, and BRAKER “pipeline C” annotations. The combined annotation was finalized using the same GeMoMa version to assign gene names according to their genomic positions. The TEs in *V. dahliae* CQ2 were annotated previously^38^, in which Helitron-like elements were identified with HelitronScanner^144^ in the TE annotation pipeline EDTA version 2.2.1^125^.

The alien index (AI) was calculated using the previously established formula AI = log((best E-value for ingroup) + e-200) - log((best E-value for outgroup) + e-200)^34^. E-values for ingroup and outgroup hits were obtained through nucleotide-to-nucleotide similarity search with Basic Local Alignment Search Tool (BLAST) version 2.16.0 + , using the ‘blastn’ command with options “-word_size 11”, “-evalue 0.05” and “-outfmt 6” with default output plus query coverage (qcovs). Genes and TEs located from the start to 1,200,000 bp on chromosome 4 (Chr4) of the CQ2 genome (10.5281/zenodo.15450312) assembly were used, and only hits covering more than 50% of the query sequences (qcovs > 50) and with an E-value lower than 0.05 were retained for alien index calculation. The E-value for a no hit was defined as 1 when either the ingroup or the outgroup had hits. AI values were not determined when both the ingroup and the outgroup had no hits. The coordinates of the queries are listed in Supplementary Data 6 with AI values. The subject genome database was constructed using all currently available Pezizomycotina genome assemblies, downloaded along with taxonomy data from the NCBI database under the accession numbers listed in Supplementary Data 7, with classification of ingroup and outgroup. Assemblies without order-level assignments were excluded. Downloads were performed using NCBI Datasets with the option ‘taxon 147538’. For the statistical analysis of the alien index (AI), genes located between 588,500 bp and 1,029,595 bp on Chr4 were designated as *Ar1h2 Starship* cargo, while the remaining genes in the core regions, excluding AGRs assigned in the previous study^38^, from the start of the chromosome to 1,200,000 bp were considered core genes. The normality and homoscedasticity of data were tested by the Shapiro–Wilk test and F test, respectively, with the R base package “stats” version 4.4.2^106^, which indicated that the AI values were non-normally distributed and exhibited unequal variance (*p* < 0.01). The statistical difference in AI values between the two groups was assessed using the Brunner-Munzel test with the R package “lawstat”^145^.

### Circular dichroism spectroscopy

Circular dichroism spectroscopy was performed in the far-UV region using a Jasco J710 instrument (Jasco Inc., Easton, MD, USA) equipped with a Peltier apparatus at 20 °C. Three spectra were collected using 0.2 mg/mL μM proteins in 20 mM Tris-HCl pH 7.5 and 150 mM NaCl buffer using a 1 mm quartz cell with a 50 nm/min scanning speed and averaged.

### Size exclusion chromatography

Size exclusion chromatography was performed on a Superdex75 10/300 column (GE Healthcare, Chicago, Illinois, USA). D*Fov* (1 mg/mL) was loaded on the column equilibrated with 20 mM Tris-HCl pH 7.5, 150 mM NaCl and connected to an HLPC AZURA system (KNAUER, Berlin, Germany). The injection volume was 500 μL, and the flow rate was 1 mL/min. The calibration curve was obtained by running the following protein standards: albumin (66.5 kDa), ovalbumin (43 kDa), chymotrypsinogen (25 kDa) and ribonuclease A (13.7 kDa). The protein eluted with a Ve = 11.7 ml corresponding to an apparent molecular weight of 35 kDa.

### Crystallization conditions and structure determination

Prior to crystallization, D*Fov* was further purified by size exclusion chromatography on a Superdex75 10/300 column (GE Healthcare, Chicago, Illinois, USA) equilibrated with 20 mM Tris-HCl pH 7.5, 150 mM NaCl. Crystallization conditions were initially screened automatically using the Oryx 4 crystallization platform (Douglas Instruments, East Garston, Berkshire, UK) by mixing equal amounts (0.4 µL) of reservoir solution and protein at two different concentrations (8.0 and 3.9 mg/mL in the SEC elution buffer). Small crystals were observed in the D2 condition of the Crystal Screen (Hampton Research, Aliso Viejo, CA, USA) after one week of incubation and further optimized by hanging drop vapor diffusion method. The best diffracting crystals were obtained by mixing 1 µL of protein solution (7.6 mg/mL) with 1 µl of reservoir (0.1 M Hepes pH 7.5, 1.4 M Sodium Citrate tribasic dihydrate) equilibrated versus 500 µL of the reservoir at 21 °C. Crystals were transferred into a mother liquor solution containing 1 mM HgCl_2_ for 12 h, then transferred into another drop containing the mother liquid and 20% glycerol for 20 s before flash-freezing in liquid N_2_. X-ray diffraction data were collected at the ESRF Synchrotron in Grenoble (FR) on the beamline ID23-1. A complete data set was collected at a wavelength of 0.992 Å. Data were processed with XDS^146^, while analysis were performed with the autoPROC toolbox^147^ and scaled with Aimless^148^ at a final resolution of 1.47 Å in the CCP4 suite^149^. Space group was determined based on systematic absences with Pointless^150^, and was P 1 2_1_ 1 with the following cell parameters (a, b, c Å–α, β, γ °): 27.8, 50.0, 72.3–90.0, 98.9, 90.0. The Matthews coefficient was 1.85 Å^3^Da^−1^, corresponding to one molecule per asymmetric unit and a solvent content of 33.5%. Experimental phasing was carried out with single-wavelength anomalous diffraction based on Hg anomalous scattering by the automatic software Crank-2^151^ as implemented in CCP4-cloud suite^149^. Iterative cycles of model building and refinement were performed with Coot^152^ and Refmac5^153^. The final model consists of 77 protein residues (1493 non-H atoms), one Hepes (4-(2-hydroxyethyl)−1-piperazine ethanesulfonic acid) molecule, three Hg atoms and 184 water molecules. The full statistics for data collection and refinement are reported in Supplementary Table 2. Coordinates and structure factors have been deposited in the Protein data bank with accession code 9FEI.

### Structural relationships and network rendering

The database of effector protein structures^52^ was downloaded from https://zenodo.org/records/7506581 and the D*Fov* protein structure was added after which structural similarities were calculated as previously described^52^.

### Reporting summary

Further information on research design is available in the Nature Portfolio Reporting Summary linked to this article.

## Supplementary information

Supplementary Information

Description of Additional Supplementary Information

Supplementary Data 1

Supplementary Data 2

Supplementary Data 3

Supplementary Data 4

Supplementary Data 5

Supplementary Data 6

Supplementary Data 7

Reporting Summary

Transparent Peer Review file

## Source data

Source Data

## Data Availability

The D*Fov* structure is available in the Protein Data Bank (PDB): 9FEI and the CQ2 genome assembly is available on GenBank under accession number GCA_004798895.1. *D-like* sequences are deposited at Zenodo [10.5281/zenodo.18375323]. The chromosomal-level assemblies of *Verticillium* strains CQ2, VLB2, and PD683 are available on Zenodo [10.5281/zenodo.15450312]. The other genome assemblies used in this study are available on NCBI under the accession numbers listed in the Supplementary Tables. Source data are provided with this paper.
