# Supplementary material for: A structurally unique effector shared between vascular wilt fungi drives cotton and olive defoliation
Source: Nat Commun. 2026 Jun 21;17:7818. doi: 10.1038/s41467-026-74504-z (PMC13439237; doi:10.1038/s41467-026-74504-z)
Supplement: Supplementary file 2 [file 41467_2026_74504_MOESM11_ESM.pdf]

nature portfolio

Peer Review File

A structurally unique effector shared between Verticillium dahliae and Fusarium oxysporum is involved in cotton and olive defoliation and virulence on other hosts

Corresponding Author: Professor Bart Thomma

This file contains all reviewer reports in order by version, followed by all author rebuttals in order by version.

Version 0:

Reviewer comments:

Reviewer #1

(Remarks to the Author)
The manuscript dissects the genetic basis of defoliating strains of Verticillium dahliae using a multidisciplinary approach. The authors identified a D pathotype specific region containing two duplicated effector genes (D effector). Knockout, double knockout and complementation experiments highlighted the importance of the D effector for defoliation in cotton and olive, and pathogenicity on Nicotiana benthamiana and Arabidopsis. Furthermore, they utilised recombinant protein to empirically demonstrate that the D effector was responsible for the defoliating phenotype. The distribution of the D effector was assessed in Verticillium dahliae where they identified D-like effectors. D-like1 was unable to trigger cotton defoliation but was able to inhibit growth of Arabidopsis. D-effector homologues were found in Fusarium species possibly transferred through a starship-mediated horizontal gene transfer mechanism. The D effector homologues from fusarium was also able to cause defoliation on cotton, watermelon and melon while cucumber remained unaffected. The authors also solved the crystal structure of the D effector homologue from Fov, which has a unique fold (loose structural homology to Tox3) that has previously not been characterised.

Overall, the study provides a complete assessment of the D effector with different assays highlighting the importance of it for defoliation with sound methodology. The linkage with Starship-mediated horizontal gene transfer demonstrates the importance of the effector not only to Verticillium but other fungal pathogens. The manuscript is well written and I enjoyed reading it. Although outside the scope of this study, the molecular mechanism of this effector and how it causes defoliation would be of interest which may be assessed in a future study.

Minor comments:

1. The proteins that were used for in planta assays were only purified by IMAC. Depending on the protein, it can be quite "dirty" without a size exclusion step. Presenting SDS-PAGE gels of the final purified proteins of all the D effector/homologues used in these assays would provide confidence about the purity of these proteins.

2. For figure 5C/D, the recombinant proteins used are purified from E. coli (D effector and D-like1 from E. coli while BSA from commercial?). I think while BSA is an ok control, a better control would have been an unrelated protein purified form E. coli in the same way to control for any contaminants (similar to using Vd2LysM as a negative control for Figure S9).

Reviewer #2

(Remarks to the Author)
Doddi, Fiorin, Li, Zannini and Sato et al. present an interesting functional and evolutionary story surrounding a newly identified D protein from Verticillium. I found the manuscript well written, well organized, and technically sound. The current narrative focuses on the identification of the D protein, its functional validation through deletion and complementation analyses, the evolutionary origin of the effector, and the determination of its structure.

While the manuscript appears aimed at a broad audience and the discussion covers the major findings of the study (which I enjoyed), it would be interesting to see a more discussion of the potential biological mechanism of the D protein and its role in association of the biology of the pathogen. Based on the data presented, the protein appears capable of inducing symptoms across multiple plant species, suggesting a relatively non-specific phytotoxin activity rather than interaction with a specific host target. The authors suggest that "[the D protein] targets a conserved plant component involved in core immune or developmental processes" (Discussion) and also refer to "host-specific activity" (Abstract and line 391). While this is certainly possible, an alternative explanation is that the protein induces symptoms through more general physiological effects, for example via vascular disruption such as xylem blockage or collapse. In this context, it would also be interesting to know whether the D protein can induce defoliation in additional plant species that are not natural hosts of Verticillium, although identifying appropriate non-host species may be challenging given the broad host range of the pathogen.

It would therefore be valuable to more explicitly contextualize the activity of the D protein within the infection biology of the pathogen. For instance, transcriptomic analysis indicates that the D gene is already highly expressed by 6 dpi (Fig. S4), whereas visible defoliation develops later. This temporal pattern may suggest that the protein primes host tissues during early stages of infection before severe symptoms appear. One possibility is that the D protein contributes to preparing host tissues for later stages of disease development, potentially facilitating the transition to more destructive phases of infection. In addition, the effect of the protein appears copy number-dependent (Fig. 3), which could be highlighted more explicitly in the discussion (Line 488-489)

Finally, the diversity of D homologs raises an interesting evolutionary question. If D has such a strong role in defoliation, what selective pressures allowed related homologs to diverge into variants that no longer induce this phenotype (Fig. 5A)? While this may be beyond the scope of definitive analysis here, the authors could discuss whether such divergence might relate to differences in host range, lifestyle, or other ecological constraints among soil-borne Verticillium species or leave these to the readers as an open question. It may also be informative to map sequence variation in ND proteins onto the predicted structures and consider/speculate whether particular substitutions correlate with the observed loss of defoliation activity.

The authors have synthesized a thoughtful discussion, and there may be word count limits imposed by the journal; therefore, I abstain from suggesting extensive restructuring. However, integrating some of these points into the existing discussion could further enrich the biological narrative of the study.

Line 51:
Moreover, or exogenous application of the purified protein, induced wilting and defoliation as well. -> Moreover, exogenous application of the purified protein induced wilting and defoliation.

Line 53:
Authors may wish to use a different word to replace "conserved". Please see my comments on the structure section.

Line 113:
The genome accession (GCA_004798895.1) appears to correspond to the assembly reported by Depotter et al. (2019; PMC6771948). However, the text states that CQ2 was sequenced and assembled in this study (Methods; Table S1). Please clarify whether this refers to a previously published assembly or a newly generated one.

Figure 1C:
The grayscale colors used to represent the different defoliation categories are somewhat difficult to distinguish (especially 75% vs 100%). Consider using more distinct colors or patterns to improve readability. This comment applies to the categorical bar plots in other figures as well.
Please include in the legend how statistical significance was calculated. This comment applies to the categorical bar plots in other figures as well.

Figure 2:
Though the key message the authors aim to convey is clear, the figure is quite dense in its current form, and the font sizes are relatively small, which makes it somewhat difficult to read. The information presented in panels A and B might be more suitable for a supplemental figure, while a more concise version could be retained in the main text. I do not insist on these changes, but I believe the same information could be presented in a clearer and more visually appealing format.

Lines 139-140:
Gene models "are" shown in blue, and the candidate effector genes "are" displayed in red, while repetitive elements "are" displayed in green.

Line 143:
'showed' -> shown

Line 163:
The D effector protein was applied at a concentration of 9 µM. Given that the protein is likely diluted during vascular uptake and transport, the chosen concentration may be reasonable. However, it would be helpful if the authors could briefly indicate whether lower concentrations were tested or how this concentration was determined.

Line 198:
This is very subtle, but “required” for olive defoliation may be more appropriate, as the complementation assays were not shown.

Line 212:
Please briefly mention how the statistical test was conducted.

Line 217:
“… strains in panel A at 21 (dpi).” -> 21 dpi

Line 225:
In this section, isolates/strains may be a more appropriate term than population.

Line 225:
Please refer to Table S1, if appropriate.

Line 229:
similarity -> identity

Line 239:
Is there any reason why the concentration was chosen as 5 µM not 9 µM as previously?

Line 241:
Please specify BSA as a control

Figure 5C:
The D-like1 treatment appears to show a transient reduction in canopy area at 2 DPI followed by partial recovery at later time points. It may be helpful to comment briefly on this pattern and whether it reflects a biologically meaningful transient growth effect or experimental variation.

Line 257:
population -> strains or isolates

Line 281:
“Within Verticillium, D homologs were found in V. alfalfae and V. longisporum, but not in other species.” Here, it may help to make the distribution pattern more explicit. The authors could clarify how many Verticillium species were surveyed and note that the D gene occurs only in a small subset of them. For example, indicating that D homologs were detected in only 3 of X Verticillium species analyzed would better illustrate the sparse and patchy distribution of the gene and further support the proposed HGT events within the genus in the following section.

Fig. S13B:
Please specify what each colored bars indicate

Line 278:
Given the analysis in the section, I wonder if the header is accurate. Perhaps, authors may choose a bit mor generic ‘Starship-associated horizontal transfer of the D gene’ or ‘Evidence for Starship-associated transfer of the D gene between Verticillium species and Fusarium’, as the starship-mediated HGT between Verticillium and Fusarium is a working model rather than explicitly supported.

Line 281:
V. alfalfae, and V. longisporum -> V. alfalfae and V. longisporum

Line 311:
oxysporumstrains -> oxysporum strains

Lines 336-348:
This paragraph, together with Fig. 6A, plays a central role in supporting the authors’ claims, but it is somewhat dense to parse quickly. Part of the difficulty arises because strains from different Verticillium species (e.g., V. alfalfae PD683 and V. longisporum VLB2) are included in the comparison with V. dahliae. Although these strains are introduced earlier in the section, readers may not immediately recognize them when interpreting Fig. 6A. Because this information is not explicitly indicated within the image, it may cause some confusion when reading the results alongside the figure.
The authors may consider adding more explicit guiding information in the main text to help readers follow the comparison more easily without going back and forth to the figure legend. For example, the sentence describing the genomic context of the D gene could briefly reference representative strains shown in Fig. 6A (e.g., “In Verticillium, the D gene is located on Ar1h2 haplotype Starships in V. dahliae strains such as VD991…”), which would help readers more directly connect the text with the figure.

Line 349-364:
My previous comment extends to this section. Indicating specific strains directly in the text would help readers follow the

comparisons more easily without having to repeatedly refer back to the figure legend.

Figure 7D.
Although I understand that space may be limited for this panel, the labels appear quite small and are difficult to read. Increasing the font size slightly would improve readability.

Line 408:
The section header states that the D effector displays a unique fold, yet the text later indicates that it shows a “distant similarity” to the effector Tox3. If “unique” here is intended to mean that the fold is not found in other experimentally determined structures, it may be helpful to clarify this point within the main text or in the header.

Line 428:
The phrase “a distant similarity” is somewhat ambiguous. Please clarify this more explicitly. Comparison of D and Tox3 using TM-align yields a TM-score of ~0.56, which generally indicates fold-level similarity. However, the structures certainly appear visually distinct (Figure S17), and the authors could briefly describe this difference.

Line 429:
Please also report the TM-score for the D–Tox3 comparison, given that TM-score is already used in this section for evaluating the AlphaFold model comparison.

Line 429-431:
It is unclear why the structural search was limited to ~5,000 AlphaFold2-predicted fungal effector structures. Structural search tools such as Foldseek already allow comparison against much larger structure databases. I recommend submitting the D protein structure to Foldseek to expand the search space and refining the text, although the results may ultimately be consistent with the current findings.

Line 433:
The phrase “a fold conserved across diverse fungal lineages” may be somewhat inaccurate. It is not yet clear whether this fold represents a conserved evolutionary family (which I think is unlikely), or whether similar folds arose independently due to functional convergence.
To me, the M protein simply shows fold-level similarities to other unrelated proteins that have happen to adopt similar folds due to limited structural space. In that sense, I agree with authors that the M protein fold is unique. In such cases, a more cautious phrasing such as “a fold observed across diverse fungal lineages” may be more appropriate. It may also be helpful to report the closest structural similarity (e.g., TM-score) to provide quantitative context. Although the D protein appears connected within the structural similarity network (Figure S17), it may emerge as a more distinct outlier under more stringent clustering thresholds. I am unable to offer suggestions, as the methods are not detailed in this section. However, discussing this possibility could help emphasize the novelty of the D protein fold.

Figure S16C:
The figure legend explains TM-score and RMSD, but the actual scores are not provided for the structural alignment.

Lines 487-489:
Authors could be more explicit here that the phenotype depends on the dosage of the gene. Please refer to my earlier discussion comment for additional context.

Lines 513 & 516:
transposable elements -> TEs

Lines 522-524:
It may also be possible that the effector does not target a specific component but cause vascular dysfunction. Please refer to my earlier discussion comment for additional context.

Line 539:
Please specify which PacBio platform/technology was used.

Line 588:
a space is needed after ‘described’

Line 669:
(67–100%), “and” 4 (dead plant)

Line 874 Data Availability:
I really appreciate that the authors transparently share the datasets prior to publication. I recommend explicitly restating the key datasets and their accession numbers to facilitate access for readers, in particular, PDB: 9FEI and GCA_004798895.1, if appropriate.

Version 1:

Reviewer comments:

Reviewer #1

(Remarks to the Author)
The manuscript dissects the genetic basis of defoliating strains of Verticillium dahliae. Using a multidisciplinary approach, the authors identified the D effector which is important for defoliation in cotton and olive and pathogenicity on Nicotiana benthamiana and Arabidopsis while D-like effectors were unable to trigger cotton defoliation. The authors build a convincing case of the D-effector being horizontally transferred via starships to other pathogenic fungi like Fusarium, which also exhibits defoliation phenotypes in cotton, watermelon and melon while cucumber remained unaffected.

After the first review, the authors have addressed all my comments and have included interesting discussions in the benefit of defoliation for Verticillium and other fungal pathogens in response to reviewer 2. Additions within the resubmitted manuscript has increased the clarity of the paper.

Reviewer #2

(Remarks to the Author)
I appreciate the authors' efforts in revising the manuscript and updating the text and figures accordingly. I found the responses to the reviewers to be thoughtful and constructive, and I do not have any further major concerns.
The remaining comments below are intended to assist with final polishing, particularly in the newly added sections, where some parts feel less refined than the original text. A careful final pass to improve clarity and consistency would further strengthen the manuscript.

I also congratulate the authors on completing this work, which clearly represents a substantial and multi-year effort from numerous authors.

Minor comments
Line 434-438: "which generally indicates fold-level similarity"
-> Given a TM-score of 0.50, I believe authors could use a more confident tone to claim the novelty of the D protein. The score is right at the broader line of typical similarity detection.

"The conserved structural core generally contributes to protein stability and proper folding… " and onward
-> A TM-score of 0.50 represents a lenient, borderline threshold for fold-level similarity and should not be interpreted as definitive. While commonly used in structural genomics, this cutoff can introduce false positives, and TM-score > 0.5 does not imply structural homology or biological similarity. In this context, the authors' interpretation may underestimate the novelty of the D protein fold. Also, please be careful not to bring "homology" based on this comparison.

The discussion of conserved cores and variable loop regions appears to rationalize this weak similarity but is generic and not supported by data in the present system. Moreover, functional inference based on structural similarity alone is insufficient, as key determinants such as surface biochemical properties are not examined.

More fundamentally, this line of reasoning is unnecessary. The structural data already support the conclusion that the D protein adopts a previously uncharacterized fungal effector structure, and overinterpretation of borderline similarity detracts from this clearer point.

A more direct framing may be appropriate. For example, the authors could state that "a detailed comparison of DFov … yields a TM-score of 0.50, which lies at the borderline for fold-level similarity." In addition, a comprehensive Foldseek search against the AlphaFold structural database did not identify matches above this threshold, supporting the structural novelty of DFov relative to currently predictable fungal protein structures.
Comparison with ~5,000 AlphaFold2-predicted structures identified several putative structural matches, but these remained within borderline similarity (TM-score ~ X). The structural similarity network places DFov in a cluster with these predicted effectors, suggesting that it adopts a fold with weak structural similarities across some fungal effectors, while remaining distinct from previously characterized structures.

Please note that this example is provided solely to illustrate my perspective and is not intended to prescribe specific wording. The authors are welcome to adapt, modify, or rewrite it as they see fit.

Line 440: "Whereas a Foldseek search with DFov did not identify any significant hits, .."
-> A comprehensive structural similarity search with Foldseek against the AlphaFold database did not…

Line 442: "several high-confidence structural homologs"
-> The term "high-confidence structural homologs" is not appropriate here, as homology implies evolutionary relatedness, which is not demonstrated. "Putative structural matches" or "structural similarities" would be more accurate.

Line 446: "These results provide the first experimental evidence for a novel structural class of fungal effectors"
-> Please be careful with wording here. I don't think the sentence reads like what authors intended to convey. The authors may consider rephrasing to indicate that DFov represents a experimentally determined, previously uncharacterized fold

Line 509: "that these variants have mutated to avoid recognition by host immune systems"

-> The interpretation that pseudogenized variants have evolved to evade host immune recognition is speculative. Although this is possible, this section currently only implies adaptive mechanisms while psuedoginization may reflect neutral processes or genome instability. I recommend authors to tone down or revise this sentence.
-> This interpretation is also somewhat inconsistent with the subsequent sentence that states "It presently remains unclear what selective pressures allowed related …", as this claim in L509 implies a specific adaptive mechanism. I recommend going over this section carefully in the final piece.

Line 512-515: "It presently remains unclear what selective pressures .."
-> This section is somewhat verbose. The authors could more directly state that the selective forces shaping D effector evolution are currently unknown, and then proceed to their speculative hypotheses.
Line 544-554:
-> I appreciate authors introduce interesting hypothesis. I would recommend final streaming lining to make this section concise.
-> Also, it is unclear what this sentence means: "Ultimately, these scenarios can be resolved once the .." Does it mean in silico screening?

Line 557: "structural homology" -> fold-level similarity

Stylistic changes
Line 109: "CQ2, ST100" -> CQ2, and ST100
Line 110: "JR2, VdLs17" -> JR2 and VdLs17
Line 130 & 132: used -> is/was used
Line 132: "V700, VdLS17" -> V700, and VdLS17
Line 227: population -> strains
Line 240: DPI -> dpi
Line 241: Arabidopsis thaliana -> A. thaliana
Line 291: D-like15, D-like27 -> D-like15, and D-like27
Line 414: plDDT -> mean/average pLDDT (or indicate with pTM-score)
Line 507: population -> strains
Line 545: infection stages, by 6 dpi -> stages by 6 dpi

Open Access This Peer Review File is licensed under a Creative Commons Attribution 4.0 International License, which permits use, sharing, adaptation, distribution and reproduction in any medium or format, as long as you give appropriate credit to the original author(s) and the source, provide a link to the Creative Commons license, and indicate if changes were made.
In cases where reviewers are anonymous, credit should be given to 'Anonymous Referee' and the source.
The images or other third party material in this Peer Review File are included in the article's Creative Commons license, unless indicated otherwise in a credit line to the material. If material is not included in the article's Creative Commons license and your intended use is not permitted by statutory regulation or exceeds the permitted use, you will need to obtain permission directly from the copyright holder.
To view a copy of this license, visit https://creativecommons.org/licenses/by/4.0/

Reviewer 1: The manuscript dissects the genetic basis of defoliating strains of Verticillium dahliae using a multidisciplinary approach. The authors identified a D pathotype specific region containing two duplicated effector genes (D effector). Knockout, double knockout and complementation experiments highlighted the importance of the D effector for defoliation in cotton and olive, and pathogenicity on Nicotiana benthamiana and Arabidopsis. Furthermore, they utilised recombinant protein to empirically demonstrate that the D effector was responsible for the defoliating phenotype. The distribution of the D effector was assessed in Verticillium dahliae where they identified D-like effectors. D-like1 was unable to trigger cotton defoliation but was able to inhibit growth of Arabidopsis. D-effector homologues were found in Fusarium species possibly transferred through a starship-mediated horizontal gene transfer mechanism. The D effector homologues from fusarium was also able to cause defoliation on cotton, watermelon and melon while cucumber remained unaffected. The authors also solved the crystal structure of the D effector homologue from Fov, which has a unique fold (loose structural homology to Tox3) that has previously not been characterised.

Overall, the study provides a complete assessment of the D effector with different assays highlighting the importance of it for defoliation with sound methodology. The linkage with Starship-mediated horizontal gene transfer demonstrates the importance of the effector not only to Verticillium but other fungal pathogens. The manuscript is well written and I enjoyed reading it. Although outside the scope of this study, the molecular mechanism of this effector and how it causes defoliation would be of interest which may be assessed in a future study.
AUTHORS: We thank the reviewer for their appreciation of our work and their supportive comments.

Reviewer 1: Minor comments:
1. The proteins that were used for in planta assays were only purified by IMAC. Depending on the protein, it can be quite "dirty" without a size exclusion step. Presenting SDS-PAGE gels of the final purified proteins of all the D effector/homologues used in these assays would provide confidence about the purity of these proteins.
AUTHORS: We have now included SDS-PAGE gels of the final purified proteins of all the D effector/homologues used in these assays, showing that all protein preparations are of high purity. These data are now included as Figure S18.

Reviewer 1: 2. For figure 5C/D, the recombinant proteins used are purified from E. coli (D effector and D-like1 from E. coli while BSA from commercial?). I think while BSA is an ok control, a better control would have been an unrelated protein purified form E. coli in the same way to control for any contaminants (similar to using Vd2LysM as a negative control for Figure S9).
AUTHORS: Indeed, BSA was bought commercially. Although we understand the point of the reviewer, we are of the opinion we have included sufficient controls to conclude that the responses that are observed upon D protein treatment are specific. As the reviewer rightfully indicated, we started off with Vd2LysM as a negative control, which confirmed that the defoliation response is D-protein specific (Figure S9). This is further confirmed in figures 5 and 7, where BSA was used as a control. We were not in the ability to use Vd2LysM as control throughout all our assays as

the amount of protein to our disposal was limited. However, we think it should be considered that, besides Vd2LysM and BSA, also the D-like protein acts as a control. This protein has been included in the assays in Figures 5 and 7, was purified in the same fashion as the D protein, yet does not show the typical D protein-specific responses. This excludes that any potential contaminants from the heterologous expression system that are co-purified are responsible for the observed phenotypes. Thus, we can firmly conclude that the responses observed upon D-protein administration are D-protein specific.

===

Reviewer 2: Doddi, Fiorin, Li, Zannini and Sato et al. present an interesting functional and evolutionary story surrounding a newly identified D protein from Verticillium. I found the manuscript well written, well organized, and technically sound. The current narrative focuses on the identification of the D protein, its functional validation through deletion and complementation analyses, the evolutionary origin of the effector, and the determination of its structure.

While the manuscript appears aimed at a broad audience and the discussion covers the major findings of the study (which I enjoyed), it would be interesting to see a more discussion of the potential biological mechanism of the D protein and its role in association of the biology of the pathogen. Based on the data presented, the protein appears capable of inducing symptoms across multiple plant species, suggesting a relatively non-specific phytotoxin activity rather than interaction with a specific host target. The authors suggest that "[the D protein] targets a conserved plant component involved in core immune or developmental processes" (Discussion) and also refer to "host-specific activity" (Abstract and line 391). While this is certainly possible, an alternative explanation is that the protein induces symptoms through more general physiological effects, for example via vascular disruption such as xylem blockage or collapse. In this context, it would also be interesting to know whether the D protein can induce defoliation in additional plant species that are not natural hosts of Verticillium, although identifying appropriate non-host species may be challenging given the broad host range of the pathogen.
AUTHORS: The referee raises some interesting thoughts. Indeed, the protein is able to induce responses in a wide range of plant species; cotton, olive, Arabidopsis, and Nicotiana for Verticillium D, and cotton, watermelon and melon for Fusarium D. Intriguingly, however, cucumber seems insensitive. The host-specificity simply comes from the fact that literature clearly describes that defoliation is a phenomenon that is restricted to particular host species; in our case cotton and olive. Although the reviewer suggests that "an alternative explanation is that the protein induces symptoms through more general physiological effects, for example via vascular disruption such as xylem blockage or collapse", we think that is perfectly compatible with our proposal that "[the D protein] targets a conserved plant component involved in core immune or developmental processes". After all, even for these "more general physiological effects", the protein will have to target (a) host component(s). Given that we can induce the symptomatology with protein administration only, we are able to exclude that this is induced through accumulation of fungal biomass. To clarify this, we now specifically state: "Its activity across diverse hosts—cotton, olive, *Nicotiana benthamiana*, and *Arabidopsis thaliana*—suggests it

targets a conserved <u>molecular</u> target involved in core immune or developmental processes, <u>and thus induces symptoms through general physiological effects</u>.". Finally, testing non-hosts is challenging these are limited (indeed, given the broad host range), and their status as non-hosts is controversial as they could be non-symptomatic hosts.

Reviewer 2: It would therefore be valuable to more explicitly contextualize the activity of the D protein within the infection biology of the pathogen. For instance, transcriptomic analysis indicates that the D gene is already highly expressed by 6 dpi (Fig. S4), whereas visible defoliation develops later. This temporal pattern may suggest that the protein primes host tissues during early stages of infection before severe symptoms appear. One possibility is that the D protein contributes to preparing host tissues for later stages of disease development, potentially facilitating the transition to more destructive phases of infection. In addition, the effect of the protein appears copy number-dependent (Fig. 3), which could be highlighted more explicitly in the discussion (Line 488-489)
AUTHORS: We thank the reviewer for the suggestion. We have now added a paragraph to the discussion staing: "Interestingly, transcriptomic analysis indicates that the *D* gene is highly expressed at early infection stages, by 6 dpi (Fig. S4), although visible defoliation symptoms in infected plants develop considerably later. One possibility is that the D protein contributes to preparing host tissues for later stages of disease development, potentially facilitating the transition to more destructive phases of infection. In this scenario, the protein primes host tissues during early stages of infection before severe symptoms appear. Alternatively, or additionally, the protein may need to accumulate to sufficient amounts or concentrations before symptomatology is induced. Finally, it may be that the physiological responses that are induced by the D protein take time to develop to ultimately culminate into defoliation symptoms. Ultimately, these scenarios can be resolved once the molecular target(s) of the D protein is identified, which could be facilitated by a three-dimensional protein structure.". The dose-dependency we have now addressed more specifically in lines 499-489 as suggested: "Quantitative phenotyping demonstrated that each copy contributes to fungal aggressiveness, indicating that duplication of the D effector is important for maintaining high virulence in D pathotype strains *and pointing towards a dosage effect to establish the defoliation phenotype*."

Reviewer 2: Finally, the diversity of D homologs raises an interesting evolutionary question. If D has such a strong role in defoliation, what selective pressures allowed related homologs to diverge into variants that no longer induce this phenotype (Fig. 5A)? While this may be beyond the scope of definitive analysis here, the authors could discuss whether such divergence might relate to differences in host range, lifestyle, or other ecological constraints among soil-borne Verticillium species or leave these to the readers as an open question. It may also be informative to map sequence variation in ND proteins onto the predicted structures and consider/speculate whether particular substitutions correlate with the observed loss of defoliation activity.
AUTHORS: The reviewer raises an interesting point that we indeed did not address, and which is linked to the open question what the benefit of the defoliation is, if any, in the first place. We have now added the following section to the discussion: "Our study revealed diverged D effector homologs throughout the *V. dahliae* population, corresponding to seven D-like alleles (Figure 5A).

Interestingly, several of the variants appear pseudogenized due to premature stop codons or a retrotransposon insertion, suggesting that these variants have mutated to avoid recognition by host immune systems in particular plant hosts. Intriguingly, the D-like1 allele that encodes a full-length D effector variant does not cause symptom on cotton and cannot induce cotton defoliation, although growth inhibition on Arabidopsis could be observed. These results indicate that functional diversification occurs among D effector homologs. It presently remains unclear what selective pressures allowed related homologs to diverge into variants that differentially induce defoliation, as it also remains unclear what the benefit of inducing defoliation is for *V. dahliae* on particular host species. One could speculate that defoliation would allow more efficient spread of the fungus over larger distances, or that the induction of the associated necrosis symptoms allows for a more extensive proliferation in host tissues. Future research will have to address whether such divergence of D effector homologs might relate to differences in host range, lifestyle, or other ecological constraints among the strains that carry the different variants". Finally, when compared to the D protein, the D-like1 protein has about 30 amino acid differences that occur rather equally distributed throughout the protein sequence. Unfortunately, at present that does not allow us to speculate on the impact of particular substitutions.

Reviewer 2: The authors have synthesized a thoughtful discussion, and there may be word count limits imposed by the journal; therefore, I abstain from suggesting extensive restructuring. However, integrating some of these points into the existing discussion could further enrich the biological narrative of the study.
AUTHORS: We think the reviewer has raised some interesting and thoughtful points that we have tried to incorporate as indicated above.

Reviewer 2: Line 51:
Moreover, or exogenous application of the purified protein, induced wilting and defoliation as well. -> Moreover, exogenous application of the purified protein induced wilting and defoliation.
AUTHORS: Modified as suggested.

Reviewer 2: Line 53:
Authors may wish to use a different word to replace "conserved". Please see my comments on the structure section.
AUTHORS: The sentence has been rephrased to omit reference to conservation, and now reads: "While structural analyses revealed that D homologs share a previously uncharacterized protein fold, they are distributed across *Verticillium* and *Fusarium* species, exhibiting functional diversification and host-specific activity".

Reviewer 2: Line 113:
The genome accession (GCA_004798895.1) appears to correspond to the assembly reported by Depotter et al. (2019; PMC6771948). However, the text states that CQ2 was sequenced and assembled in this study (Methods; Table S1). Please clarify whether this refers to a previously published assembly or a newly generated one.

AUTHORS: This work has been long in the making, and this section was written as part of a PhD thesis, prior to the Depotter et al. 2019 publication. This mistake has now been corrected and we now refer to the Depotter et al. 2019 publication, in the text as well as in Table S1.

Reviewer 2: Figure 1C:
The grayscale colors used to represent the different defoliation categories are somewhat difficult to distinguish (especially 75% vs 100%). Consider using more distinct colors or patterns to improve readability. This comment applies to the categorical bar plots in other figures as well.
Please include in the legend how statistical significance was calculated. This comment applies to the categorical bar plots in other figures as well.
AUTHORS: The modifications have been made to all categorical bar plots as requested (Figure 1C and 3B; Figure S3). Significance levels were calculated based on 5,000 bootstrap replicates of the median difference between random scores of two treatments and different letter labels indicate statistically significant differences ($P < 0.05$).

Reviewer 2: Figure 2:
Though the key message the authors aim to convey is clear, the figure is quite dense in its current form, and the font sizes are relatively small, which makes it somewhat difficult to read. The information presented in panels A and B might be more suitable for a supplemental figure, while a more concise version could be retained in the main text. I do not insist on these changes, but I believe the same information could be presented in a clearer and more visually appealing format.
AUTHORS: We have simplified the A and B panels such that these are less condense, as suggested by the reviewer. The original A and B panels are now presented in Figure S4.

Reviewer 2: Lines 139-140:
Gene models "are" shown in blue, and the candidate effector genes "are" displayed in red, while repetitive elements "are" displayed in green.
AUTHORS: Modified as suggested.

Reviewer 2: Line 143:
'showed' -> shown
AUTHORS: Modified as suggested.

Reviewer 2: Line 163:
The D effector protein was applied at a concentration of 9 µM. Given that the protein is likely diluted during vascular uptake and transport, the chosen concentration may be reasonable. However, it would be helpful if the authors could briefly indicate whether lower concentrations were tested or how this concentration was determined.
AUTHORS: We have initially reasoned that we should work with low-micromolar concentrations to be able to expect physiological relevance. These concentrations are based on typical effects that we see for other types of fungal effectors, such as those that display antimicrobial activity at low micromolar concentrations (E.g. Snelders et al. Nature Plants 2020; Snelders et al., PNAS 2021).

After the initial assays that we performed at 9 µM, we tested the activity of the D effector protein from the cotton-pathogenic *F. oxysporum* f. sp. *vasinfectum* (D*Fov*), for which we also tested a concentration range. For D*Fov* we determined that even with 2,5 µM we observed similar physiological responses. We therefore reduced the amounts in our assays to 5 µM for further assays that are described in the manuscript so that we could use our proteins more sparsely. However, arguably, all concentrations used in the manuscript, between 5 and 9 µM, fall within the same (low micromolar) range. The assay where we tested physiological effects of lower concentrations of D*Fov* protein is now shown as Figure S15.

Reviewer 2: Line 198:
This is very subtle, but "required" for olive defoliation may be more appropriate, as the complementation assays were not shown.
AUTHORS: Modified as suggested.

Reviewer 2: Line 212:
Please briefly mention how the statistical test was conducted.
AUTHORS: All statistics in this figure has been performed with a Student's t-test; $P < 0.05$.

Reviewer 2: Line 217:
"… strains in panel A at 21 (dpi)." -> 21 dpi
AUTHORS: Modified as suggested.

Reviewer 2: Line 225:
In this section, isolates/strains may be a more appropriate term than population.
AUTHORS: We modified the section header to: "D effector homologs occur across *V. dahliae* strains".

Reviewer 2: Line 225:
Please refer to Table S1, if appropriate.
AUTHORS: Modified as suggested.

Reviewer 2: Line 229:
similarity -> identity
AUTHORS: Modified as suggested.

Reviewer 2: Line 239:
Is there any reason why the concentration was chosen as 5 µM not 9 µM as previously?
AUTHORS: As described above, after testing a small concentration range for the D*Fov* homolog, we reduced the amounts in our assays to 5 µM so that we used our protein more sparsely. The assay where we tested physiological effects of the concentration range of D*Fov* protein is now shown as Figure S15.

Reviewer 2: Line 241:
Please specify BSA as a control
AUTHORS: defined as bovine serum albumin.

Reviewer 2: Figure 5C:
The D-like1 treatment appears to show a transient reduction in canopy area at 2 DPI followed by partial recovery at later time points. It may be helpful to comment briefly on this pattern and whether it reflects a biologically meaningful transient growth effect or experimental variation.
AUTHORS: We believe this reduction in canopy area is caused by a transient epinastic movement of the cotyledons after exposure to the D-like protein (also observed in Figure 7A). Thuss, we believe it does not reflect a genuine reduction in canopy area, but rather a different orientation of the leaves. We now state in the manuscript: "While D protein induced wilting, chlorosis, and >50% leaf detachment by 16 days (Figure 5C), D-like1 caused no symptoms *other than a slight and transient epinastic movement of the cotyledons at 2 DPI*, indicating it cannot trigger cotton defoliation".

Reviewer 2: Line 257:
population -> strains or isolates
AUTHORS: Replaces with "across *V. dahliae* strains.

Reviewer 2: Line 281:
"Within Verticillium, D homologs were found in V. alfalfae and V. longisporum, but not in other species." Here, it may help to make the distribution pattern more explicit. The authors could clarify how many Verticillium species were surveyed and note that the D gene occurs only in a small subset of them. For example, indicating that D homologs were detected in only 3 of X Verticillium species analyzed would better illustrate the sparse and patchy distribution of the gene and further support the proposed HGT events within the genus in the following section.
AUTHORS: We have now rephrased the sentence as follows: "Within *Verticillium*, *D* homologs were detected in only 3 of the 10 *Verticillium* species, as they were additionally found in *V. alfalfae* and *V. longisporum*, but not in other species".

Reviewer 2: Fig. S13B:
Please specify what each colored bars indicate
AUTHORS: The red bars indicate the D homologs and the grey bars the GAPDH homologs. This is now specified in the legend.

Reviewer 2: Line 278:
Given the analysis in the section, I wonder if the header is accurate. Perhaps, authors may choose a bit mor generic 'Starship-associated horizontal transfer of the D gene' or 'Evidence for Starship-associated transfer of the D gene between Verticillium species and Fusarium', as the starship-mediated HGT between Verticillium and Fusarium is a working model rather than explicitly supported.

AUTHORS: We changed the header to: "*Starship* giant transposon-mediated horizontal transfer of the *D* gene".

Reviewer 2: Line 281:
V. alfalfae, and V. longisporum -> V. alfalfae and V. longisporum
AUTHORS: Modified as suggested.

Reviewer 2: Line 311:
oxysporumstrains -> oxysporum strains
AUTHORS: Modified as suggested.

Reviewer 2: Lines 336-348:
This paragraph, together with Fig. 6A, plays a central role in supporting the authors' claims, but it is somewhat dense to parse quickly. Part of the difficulty arises because strains from different Verticillium species (e.g., V. alfalfae PD683 and V. longisporum VLB2) are included in the comparison with V. dahliae. Although these strains are introduced earlier in the section, readers may not immediately recognize them when interpreting Fig. 6A. Because this information is not explicitly indicated within the image, it may cause some confusion when reading the results alongside the figure.
The authors may consider adding more explicit guiding information in the main text to help readers follow the comparison more easily without going back and forth to the figure legend. For example, the sentence describing the genomic context of the D gene could briefly reference representative strains shown in Fig. 6A (e.g., "In Verticillium, the D gene is located on Ar1h2 haplotype Starships in V. dahliae strains such as VD991…"), which would help readers more directly connect the text with the figure.
AUTHORS: We appreciate that the information provided in this section is dense and the Figure contains a lot of information too. We have tried to balance which info to provide in the Figure itself, without making it too crowded, and what info to provide in the legend, so indeed species references are not explicitly detailed in the Figure to make sure these remain legible and digestible. As suggested, we have included somewhat more context in the main text now to accommodate the request. We now state: "In the *Verticillium* genus, the *D* gene is located on *Ar1h2* haplotype *Starships*, such as in *V. dahliae* strains CQ2, VD991, and JR2, but also *V. longisporum* strain VLB2, and *V. alfalfae* strain PD683 (Figure 6A), whereas *D-like* orthologs are not."

Reviewer 2: Line 349-364:
My previous comment extends to this section. Indicating specific strains directly in the text would help readers follow the comparisons more easily without having to repeatedly refer back to the figure legend.
AUTHORS: We now refer to the specific strains in the main text as suggested by the referee.

Reviewer 2: Figure 7D.
Although I understand that space may be limited for this panel, the labels appear quite small and are

difficult to read. Increasing the font size slightly would improve readability.
AUTHORS: The Figure panel has been modified to improve legibility.

Reviewer 2: Line 408:
The section header states that the D effector displays a unique fold, yet the text later indicates that it shows a "distant similarity" to the effector Tox3. If "unique" here is intended to mean that the fold is not found in other experimentally determined structures, it may be helpful to clarify this point within the main text or in the header.
AUTHORS: We rephrased the section header to "The D effector defines a novel fungal effector family".

Reviewer 2: Line 428:
The phrase "a distant similarity" is somewhat ambiguous. Please clarify this more explicitly. Comparison of D and Tox3 using TM-align yields a TM-score of ~0.56, which generally indicates fold-level similarity. However, the structures certainly appear visually distinct (Figure S17), and the authors could briefly describe this difference.
AUTHORS: Based on the suggestions of the reviewer we now say: "A detailed comparison of D*Fov* and Tox3 using TM-align yielded a TM-score of ~0.50 and an RMSD of 3.66, which generally indicates fold-level similarity (Figure S17). While the core β-sandwich is conserved between the two proteins, significant structural differences occur in the loop regions. The conserved structural core generally contributes to protein stability and proper folding, while the more plastic and variable loop regions likely allow for functional specialisation and host-target recognition[52,53], likely enabling D*Fov* and Tox3 to maintain a similar fold while fulfilling distinct biological functions."

Reviewer 2: Line 429:
Please also report the TM-score for the D–Tox3 comparison, given that TM-score is already used in this section for evaluating the AlphaFold model comparison.
AUTHORS: The TM-score for D-Tox3 comparison has now been included in the text ("A detailed comparison of D*Fov* and Tox3 using TM-align yielded a TM-score of 0.50 and an RMSD of 3.66….") as well as in Figure S17, together with the those for the D-AltA1 comparison (TM-score 0.39 and RMSD 4.22).

Reviewer 2: Line 429-431:
It is unclear why the structural search was limited to ~5,000 AlphaFold2-predicted fungal effector structures. Structural search tools such as Foldseek already allow comparison against much larger structure databases. I recommend submitting the D protein structure to Foldseek to expand the search space and refining the text, although the results may ultimately be consistent with the current findings.
AUTHORS: Foldseek searches did not identify any significant hits. Therefore, we expanded our search to include a curated dataset of predicted fungal effector structures, building on the notion that effectors that lack sequence homology may still display structural similarity, as demonstrated by several studies (de Guillen PLoS Pathogens doi: 10.1371/journal.ppat.1005228;

Seong et al., Nature Microbiology 2023 doi: 10.1038/s41564-022-01287-6; Derbyshire and Raffaele, Nature Communications doi: 10.1038/s41467-023-40949-9). We now changed to the text such that it reads: "Whereas a Foldseek search with D*Fov* did not identify any significant hits, we compared D*Fov* against a database of ~5,000 AlphaFold2-predicted fungal effector structures[52], revealing several high-confidence structural homologs (Z-scores up to 12.4) in *Claviceps purpurea*, *Epichloe festucae*, and *F. oxysporum* f. sp. *conglutinans* (Table S11). The structural similarity network placed D*Fov* in a cluster with these predicted effectors, indicating that D*Fov* adopts a fold that is observed across diverse fungal lineages but dissimilar to previously determined effector protein structures (Figure S17). These results provide the first experimental evidence for a novel structural class of fungal effectors, validating its existence beyond *in silico* predictions and establishing a framework for functional characterization of D effector proteins."

Reviewer 2: Line 433:
The phrase "a fold conserved across diverse fungal lineages" may be somewhat inaccurate. It is not yet clear whether this fold represents a conserved evolutionary family (which I think is unlikely), or whether similar folds arose independently due to functional convergence.
To me, the M protein simply shows fold-level similarities to other unrelated proteins that have happen to adopt similar folds due to limited structural space. In that sense, I agree with authors that the M protein fold is unique. In such cases, a more cautious phrasing such as "a fold observed across diverse fungal lineages" may be more appropriate. It may also be helpful to report the closest structural similarity (e.g., TM-score) to provide quantitative context. Although the D protein appears connected within the structural similarity network (Figure S17), it may emerge as a more distinct outlier under more stringent clustering thresholds. I am unable to offer suggestions, as the methods are not detailed in this section. However, discussing this possibility could help emphasize the novelty of the D protein fold.
AUTHORS: We modified the phrasing as suggested such that it now reads: "The structural similarity network placed D*Fov* in a cluster with these predicted effectors, indicating that D*Fov* adopts a fold that is observed across diverse fungal lineages but dissimilar to previously determined effector protein structures (Figure S17)." As discussed above, we now also provide TM-scores for the closest homologs.

Reviewer 2: Figure S16C:
The figure legend explains TM-score and RMSD, but the actual scores are not provided for the structural alignment.
AUTHORS: The figure has been revised and now contains TM (0.42) and RMSD (4.07) scores.

Reviewer 2: Lines 487-489:
Authors could be more explicit here that the phenotype depends on the dosage of the gene. Please refer to my earlier discussion comment for additional context.
AUTHORS: We have now modified the text such that it reads: "Quantitative phenotyping demonstrated that each copy contributes to fungal aggressiveness, indicating that duplication of

the D effector is important for maintaining high virulence in D pathotype strains *and pointing towards a dosage effect to establish the defoliation phenotype*."

Reviewer 2: Lines 513 & 516:
transposable elements -> TEs
AUTHORS: Modified as suggested.

Reviewer 2: Lines 522-524:
It may also be possible that the effector does not target a specific component but cause vascular dysfunction. Please refer to my earlier discussion comment for additional context.
AUTHORS: We agree, and perhaps the reviewer has mis-read. We are not referring to a compartment, but to a "component". To prevent confusion, we now instead refer to a "molecular target".

Reviewer 2: Line 539:
Please specify which PacBio platform/technology was used.
AUTHORS: *Verticillium dahliae* strain CQ2 was sequenced on the PacBio RSII platform. This info is now added to the respective line.

Reviewer 2: Line 588:
a space is needed after 'described'
AUTHORS: Modified as suggested.

Reviewer 2: Line 669:
(67–100%), "and" 4 (dead plant)
AUTHORS: Modified as suggested.

Reviewer 2: Line 874 Data Availability:
I really appreciate that the authors transparently share the datasets prior to publication. I recommend explicitly restating the key datasets and their accession numbers to facilitate access for readers, in particular, PDB: 9FEI and GCA_004798895.1, if appropriate.
AUTHORS: We now added the statement: "The D*Fov* structure is available in the Protein Data Bank (PDB): 9FEI and the CQ2 genome assembly is available on GenBank under accession number GCA_004798895.1".

Reviewer 1: The manuscript dissects the genetic basis of defoliating strains of Verticillium dahliae. Using a multidisciplinary approach, the authors identified the D effector which is important for defoliation in cotton and olive and pathogenicity on Nicotiana benthamiana and Arabidopsis while D-like effectors were unable to trigger cotton defoliation. The authors build a convincing case of the D-effector being horizontally transferred via starships to other pathogenic fungi like Fusarium, which also exhibits defoliation phenotypes in cotton, watermelon and melon while cucumber remained unaffected.

After the first review, the authors have addressed all my comments and have included interesting discussions in the benefit of defoliation for Verticillium and other fungal pathogens in response to reviewer 2. Additions within the resubmitted manuscript has increased the clarity of the paper.
AUTHORS: We thank the reviewer for their appreciation of our work and their supportive comments.
==

Reviewer 2: I appreciate the authors' efforts in revising the manuscript and updating the text and figures accordingly. I found the responses to the reviewers to be thoughtful and constructive, and I do not have any further major concerns.
The remaining comments below are intended to assist with final polishing, particularly in the newly added sections, where some parts feel less refined than the original text. A careful final pass to improve clarity and consistency would further strengthen the manuscript.

I also congratulate the authors on completing this work, which clearly represents a substantial and multi-year effort from numerous authors.
AUTHORS: We thank the reviewer for their appreciation of our work and their supportive comments.

Reviewer 2: Minor comments
Line 434-438: "which generally indicates fold-level similarity"
-> Given a TM-score of 0.50, I believe authors could use a more confident tone to claim the novelty of the D protein. The score is right at the broader line of typical similarity detection.
AUTHORS: We thank the reviewer for their encouragement and have rephrased the statement accordingly.

Reviewer 2: "The conserved structural core generally contributes to protein stability and proper folding… " and onward
-> A TM-score of 0.50 represents a lenient, borderline threshold for fold-level similarity and should not be interpreted as definitive. While commonly used in structural genomics, this cutoff can introduce false positives, and TM-score > 0.5 does not imply structural homology or biological similarity. In this context, the authors' interpretation may underestimate the novelty of the D protein fold. Also, please be careful not to bring "homology" based on this comparison.
The discussion of conserved cores and variable loop regions appears to rationalize this weak similarity but is generic and not supported by data in the present system. Moreover, functional inference based on

structural similarity alone is insufficient, as key determinants such as surface biochemical properties are not examined.
More fundamentally, this line of reasoning is unnecessary. The structural data already support the conclusion that the D protein adopts a previously uncharacterized fungal effector structure, and overinterpretation of borderline similarity detracts from this clearer point.
A more direct framing may be appropriate. For example, the authors could state that "a detailed comparison of DFov … yields a TM-score of 0.50, which lies at the borderline for fold-level similarity." In addition, a comprehensive Foldseek search against the AlphaFold structural database did not identify matches above this threshold, supporting the structural novelty of DFov relative to currently predictable fungal protein structures.
Comparison with ~5,000 AlphaFold2-predicted structures identified several putative structural matches, but these remained within borderline similarity (TM-score ~ X). The structural similarity network places DFov in a cluster with these predicted effectors, suggesting that it adopts a fold with weak structural similarities across some fungal effectors, while remaining distinct from previously characterized structures.
Please note that this example is provided solely to illustrate my perspective and is not intended to prescribe specific wording. The authors are welcome to adapt, modify, or rewrite it as they see fit.
AUTHORS: We have rephrased the section according to the suggestions of the reviewer.

Reviewer 2: Line 440: "Whereas a Foldseek search with DFov did not identify any significant hits, .."
-> A comprehensive structural similarity search with Foldseek against the AlphaFold database did not…
AUTHORS: The statement has been rephrased as suggested.

Reviewer 2: Line 442: "several high-confidence structural homologs"
-> The term "high-confidence structural homologs" is not appropriate here, as homology implies evolutionary relatedness, which is not demonstrated. "Putative structural matches" or "structural similarities" would be more accurate.
AUTHORS: The statement has been rephrased accordingly.

Reviewer 2: Line 446: "These results provide the first experimental evidence for a novel structural class of fungal effectors"
-> Please be careful with wording here. I don't think the sentence reads like what authors intended to convey. The authors may consider rephrasing to indicate that DFov represents a experimentally determined, previously uncharacterized fold
AUTHORS: The statement has been rephrased as suggested.

Reviewer 2: Line 509: "that these variants have mutated to avoid recognition by host immune systems"
-> The interpretation that pseudogenized variants have evolved to evade host immune recognition is speculative. Although this is possible, this section currently only implies adaptive mechanisms while

psuedoginization may reflect neutral processes or genome instability. I recommend authors to tone down or revise this sentence.
-> This interpretation is also somewhat inconsistent with the subsequent sentence that states “It presently remains unclear what selective pressures allowed related …”, as this claim in L509 implies a specific adaptive mechanism. I recommend going over this section carefully in the final piece.
AUTHORS: The section has been carefully revised and rephrased as suggested.

Reviewer 2: Line 512-515: “It presently remains unclear what selective pressures ..”
-> This section is somewhat verbose. The authors could more directly state that the selective forces shaping D effector evolution are currently unknown, and then proceed to their speculative hypotheses.
Line 544-554:
-> I appreciate authors introduce interesting hypothesis. I would recommend final streaming lining to make this section concise.
-> Also, it is unclear what this sentence means: “Ultimately, these scenarios can be resolved once the ..” Does it mean in silico screening?
AUTHORS: Both sections have been streamlined and condensed, leading to sections that are more explicit and to the point.

Reviewer 2: Line 557: “structural homology” -> fold-level similarity
AUTHORS: Modified as suggested.

Reviewer 2: Stylistic changes
Line 109: “CQ2, ST100” -> CQ2, and ST100
Line 110: “JR2, VdLs17” -> JR2 and VdLs17
Line 130 & 132: used -> is/was used
Line 132: “V700, VdLS17” -> V700, and VdLS17
Line 227: population -> strains
Line 240: DPI -> dpi
Line 241: Arabidopsis thaliana -> A. thaliana
Line 291: D-like15, D-like27 -> D-like15, and D-like27
Line 414: plDDT -> mean/average pLDDT (or indicate with pTM-score)
Line 507: population -> strains
Line 545: infection stages, by 6 dpi -> stages by 6 dpi
AUTHORS: These issues have been checked.
