# Supplementary material for: A structurally unique effector shared between vascular wilt fungi drives cotton and olive defoliation
Source: Nat Commun. 2026 Jun 21;17:7818. doi: 10.1038/s41467-026-74504-z (PMC13439237; doi:10.1038/s41467-026-74504-z)
Supplement: Supplementary file 3 [file 41467_2026_74504_MOESM1_ESM.pdf]

SUPPORTING INFORMATION

A structurally unique effector shared between *Verticillium dahliae* and *Fusarium oxysporum* is involved in cotton defoliation and virulence on other hosts

Andrea Doddi[1,2,#], Gabriel Lorencini Fiorin[3,#], Jinling Li[3,#], Ilaria Zannini[1,#], Yukiyo Sato[2,#], Giulia Lancia[1], Giacomo Giuliari[1], Carmen Gómez-Lama Cabanás[4], Antonio Valverde-Corredor[4], Tingli Liu[5,ψ], Hui Tian[2,3], Grardy C.M. van den Berg[3], Jesús Mercado-Blanco[4,†], Baolong Zhang[5], Michael F. Seidl[6], Martijn Rep[7], Wessel Groot[7], Maria Carmela Bonaccorsi Di Patti[8], Francesca Troilo[9], Adele Di Matteo[9], Giorgio Giardina[8], Massimo Reverberi[1], Longfu Zhu[10,11, *], Luigi Faino[1,$,*] and Bart P.H.J. Thomma[2,$,*]

<u>CONTENT:</u>

Supplemental Figures 1 – 19

Supplemental Tables 1 – 4

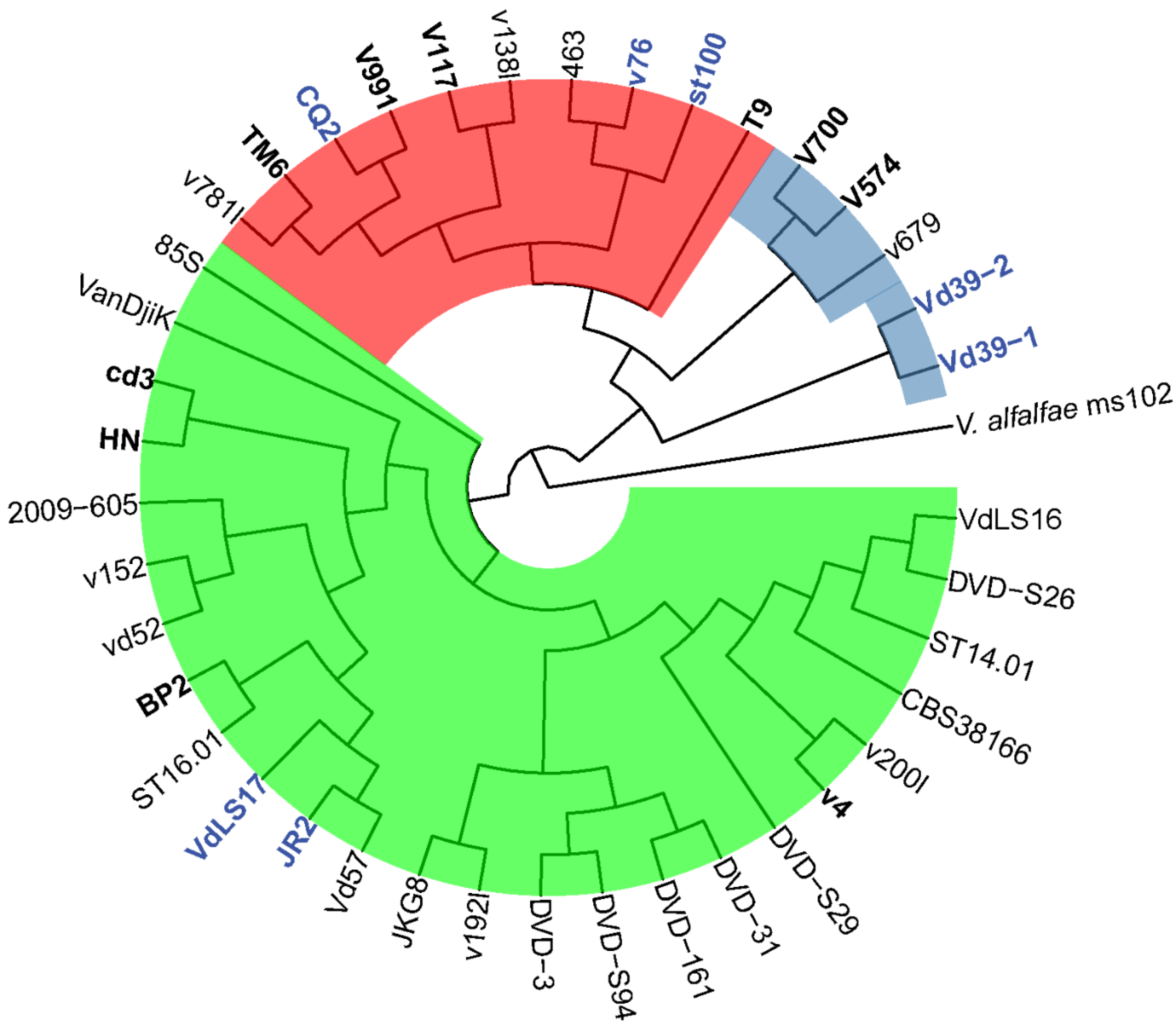

Figure S1. Phylogenetic tree of in house-sequenced *V. dahliae* strains. *V. dahliae* strains clustering with known D pathotype strains (TM6, V991, V117 and T9) are showed in lightred, while strains clustering with known ND pathotype strains (V4, BP2, cd3 and HN) are showed in light-green. Strains that distinct from the D and ND pathotype groups are showed in light-blue. Strains that used for phenotypic characterization in this work are display in bold with blue color. Phylogenetic relationship between sequenced *V. dahliae* strains was inferred using RealPhy (Bertels et al., 2014) and *V. dahliae* strain JR2 used as reference. *V. alfalfae* ms102 was used as root of the tree.

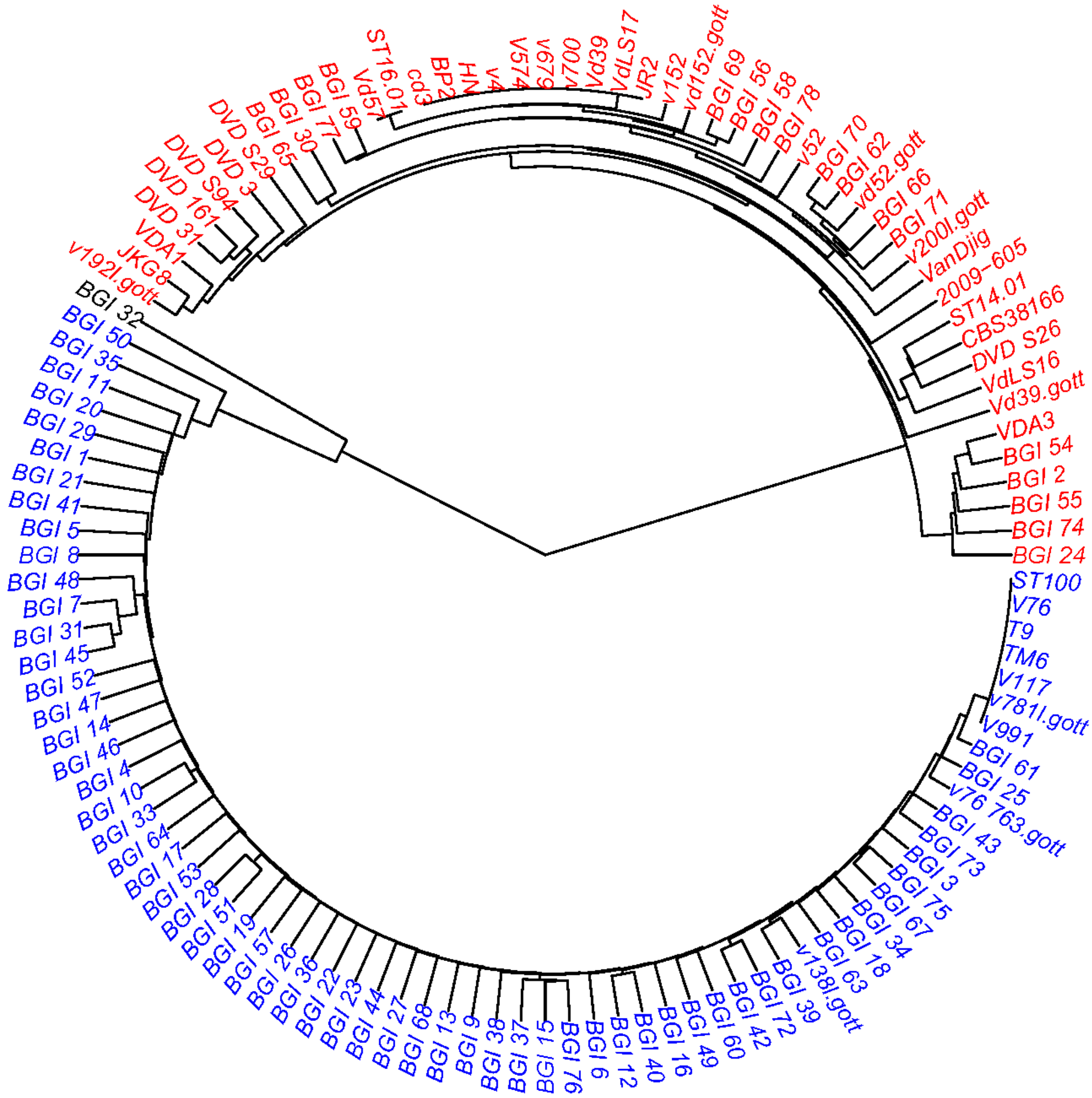

Figure S2. Clustering analysis of seventy-two sequenced *V. dahliae* strains. Sequences of seventy-two *V. dahliae* strains were aligned on the assembled genome of CQ2 and clustered in three groups based on presence/absence polymorphism. Strains clustering with known D pathotype strains are displayed in blue, while strains clustering with ND pathotype strains are showed in red. Strain BGI_32 that is the most divergent from the two groups showed in black.

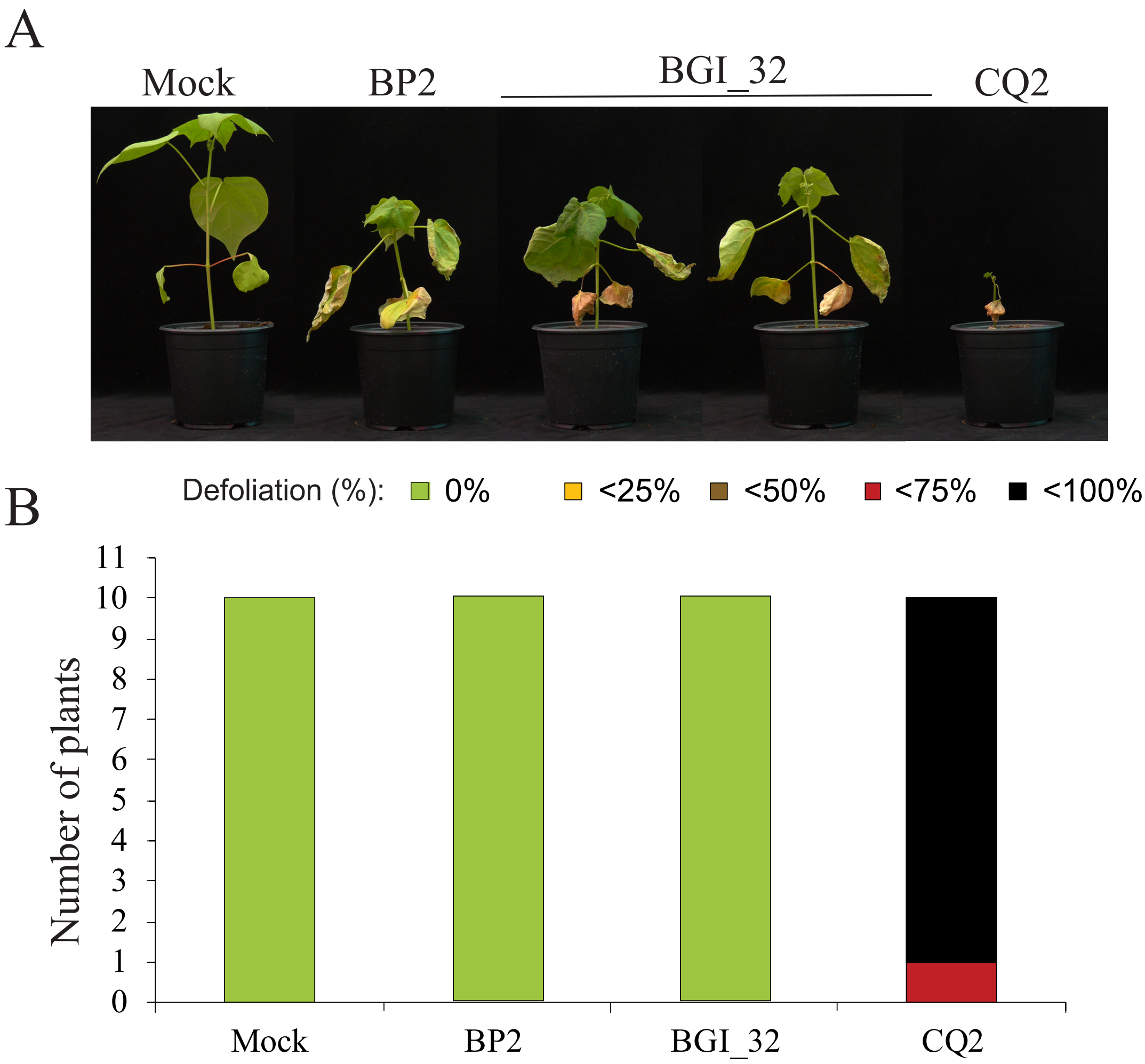

Figure S3. Phenotype of cotton plants inoculated with *V. dahliae* strain BGI32. (A) Typical phenotype of cotton plants (cv. Xinluzao63) upon mock-inoculation or inoculation with BP2, BGI32 and CQ2 at 28 days post inoculation (dpi). ND pathotype strain BP2 and D pathotype strain CQ2 were used as inoculation controls. (B) Defoliation was classified as 0 (0% leaf drop off), 1 (<25% leaf drop off), 2 (<50% leaf drop off), 3 (<75% leaf drop off) and 4 (<100% leaf drop off). Lower case letters denote significant grouping evaluated by one-way ANOVA followed by Tukey's HSD post-hoc test (two-sided, adjusted for multiple comparisons within the family of pairwise contrasts; $\alpha = 0.05$; p-Value=<2e-16). Inoculation experiments were performed with ten plants for each fungal strain and repeated twice independently with similar results.

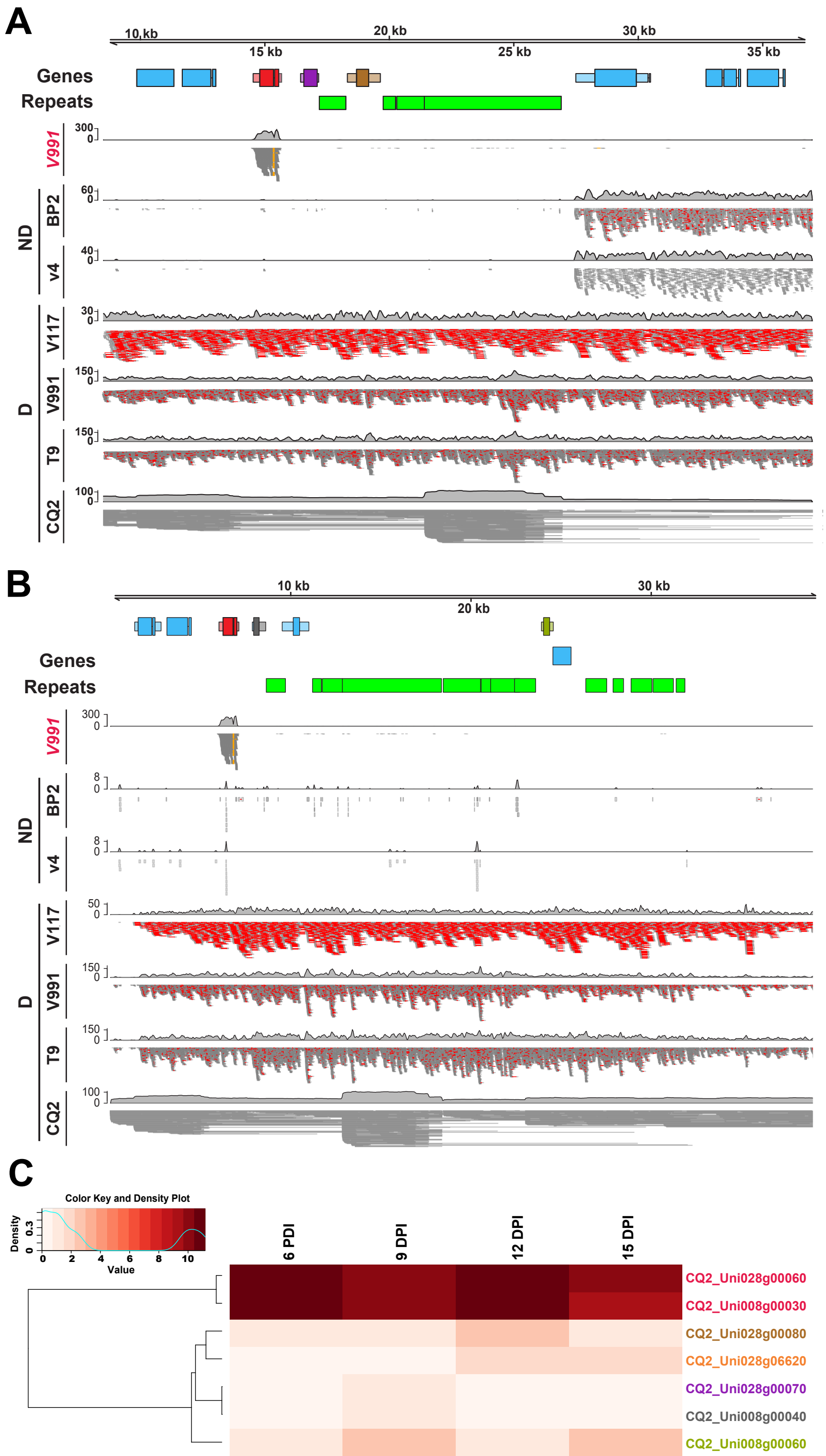

Figure S4. Schematic representation of the genomic region with two candidate effector genes and expression analysis of seven candidate genes. (A-B) Schematic representation of genomic region (Unitig_28 and Unitig_8) with two candidate effector genes displayed in red. RNAseq reads from cotton plants infected by a D pathotype strain (V991 in red) that only mapped to candidate gene are showed in dark grey. Reads from DNAseq of ND pathotype strains (V4 and BP2) and D pathotype strains (V117, V991 and T9) are mapped onto the CQ2 assembly. (C) Heatmap showing the expression level of each gene during a time course of cotton infected by D pathotype strain V991 at 6, 9, 12 and 15 days post inoculation (DPI). The scaled expression values are color-coded according to the scale bar in the left top corner. The colours of the gene names refer to the coloured gene models in panels A and B.

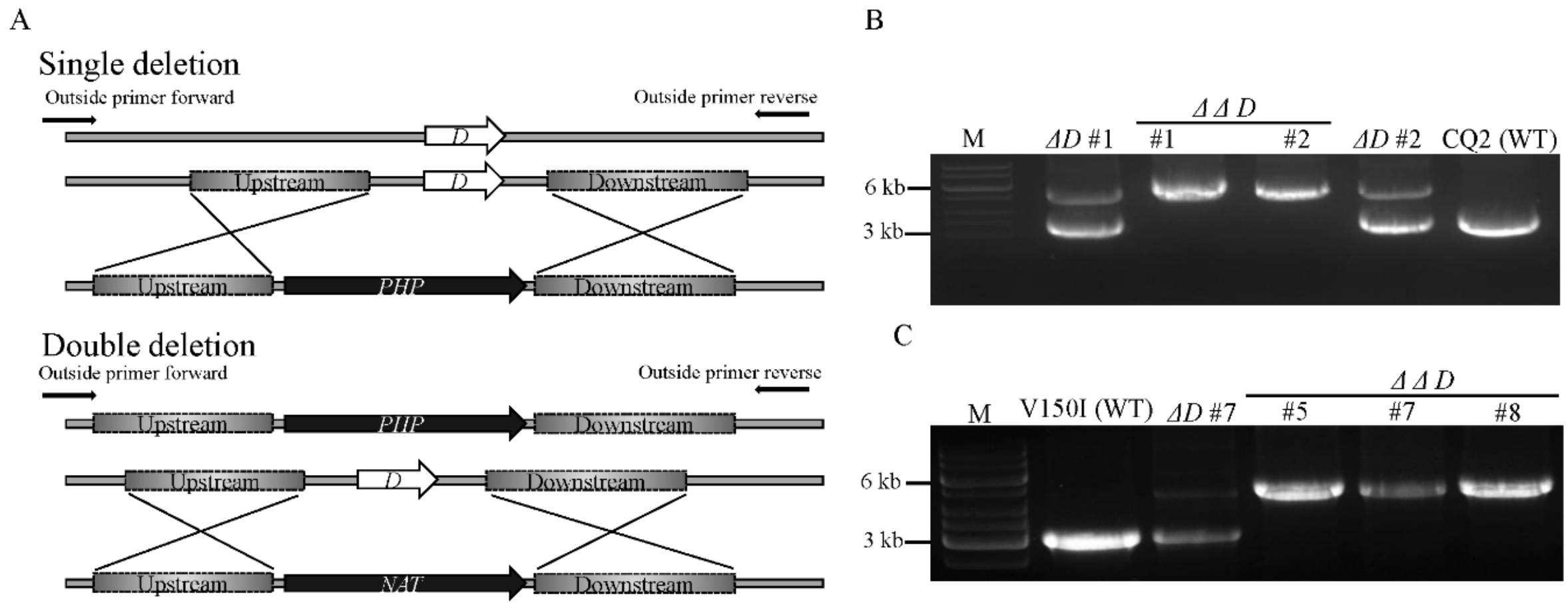

Figure S5. Construction and verification of *D* single and double deletion mutants. (A) Schematic representation of the homologous recombination events to establish targeted replacement of *D* gene with phosphotransferase (HPH) and the nourseothricin resistance gene cassette (NAT). (B) Verification of D single deletion (Δ*D*) and double deletion mutants (ΔΔ*D*) in *V. dahliae* cotton defoliating strain CQ2 by PCR. Amplicons generated with outside primers indicated in panel A are shown for wild type strain CQ2 (WT), two Δ*D* mutants (#1 and #2) and two ΔΔ*D* mutants (#1 and #2). (C) Verification of *D* single deletion (Δ*D*) and double deletion mutants (ΔΔ*D*) in *V. dahliae* olive defoliating strain V150I by PCR. Amplicons generated with outside primers indicated in panel A are shown for wild-type strain V150I (WT), one Δ*D* mutant (#7) and three ΔΔ*D* mutants (#5, #7 and #8).

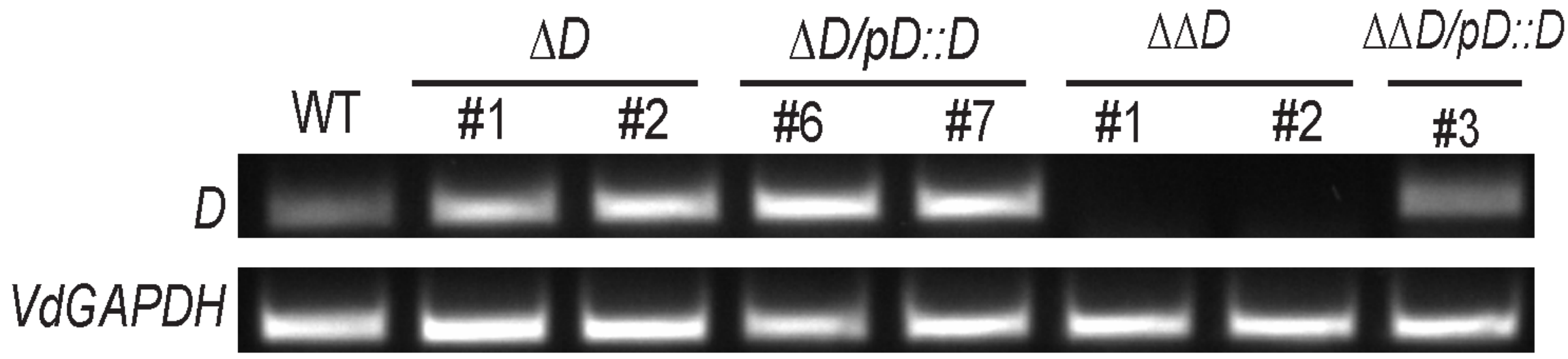

Figure S6. Detection of *D* gene transcripts in various *D* deletion and complementation strains. Amplification of *D* gene fragment (from left to right) from cDNA in wild type strain CQ2 (WT), two Δ*D* strains (#1 and #2), two Δ*D* complementation strains (Δ*D*/p*D::D* #1 and #2), two ΔΔ*D* strains (#1 and #2) and one ΔΔ*D* complementation strain (ΔΔ*D*/p*D*::*D* #3). *V. dahliae GAPDH* gene was used as endogenous control.

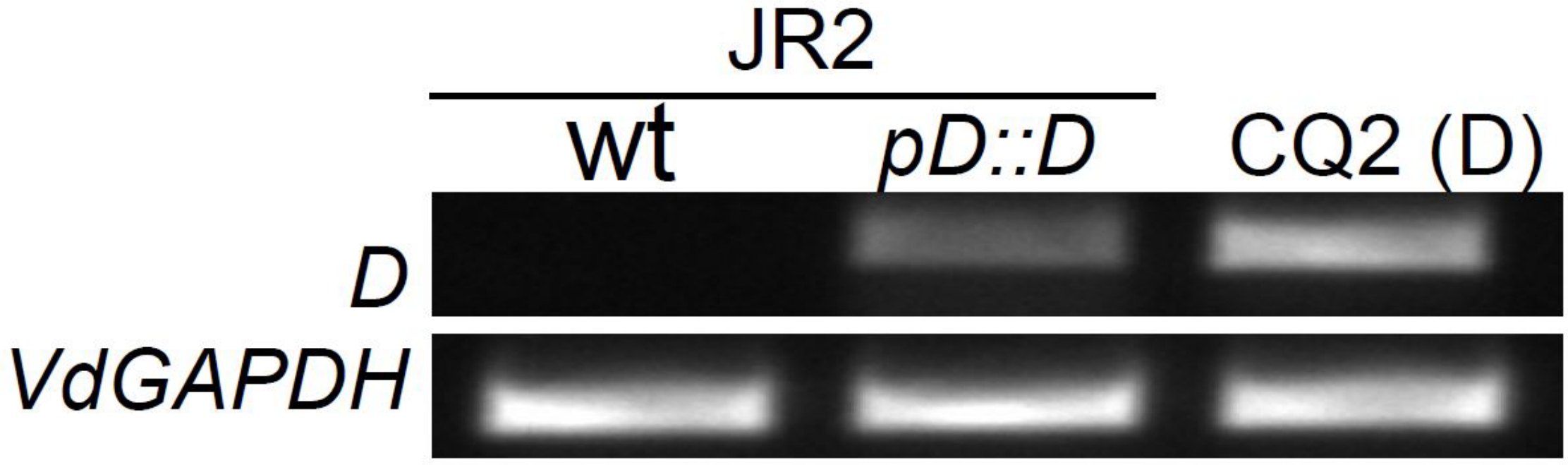

Figure S7. Expression of *D* gene (p*D*::*D*) in ND pathotype strain JR2. Amplification of *D* gene fragment (from left to right) from cDNA in wild type strain JR2, one *D* expression (p*D::D*) transformant of JR2 and CQ2 (used as positive control). *V. dahliae GAPDH* gene was used as endogenous control.

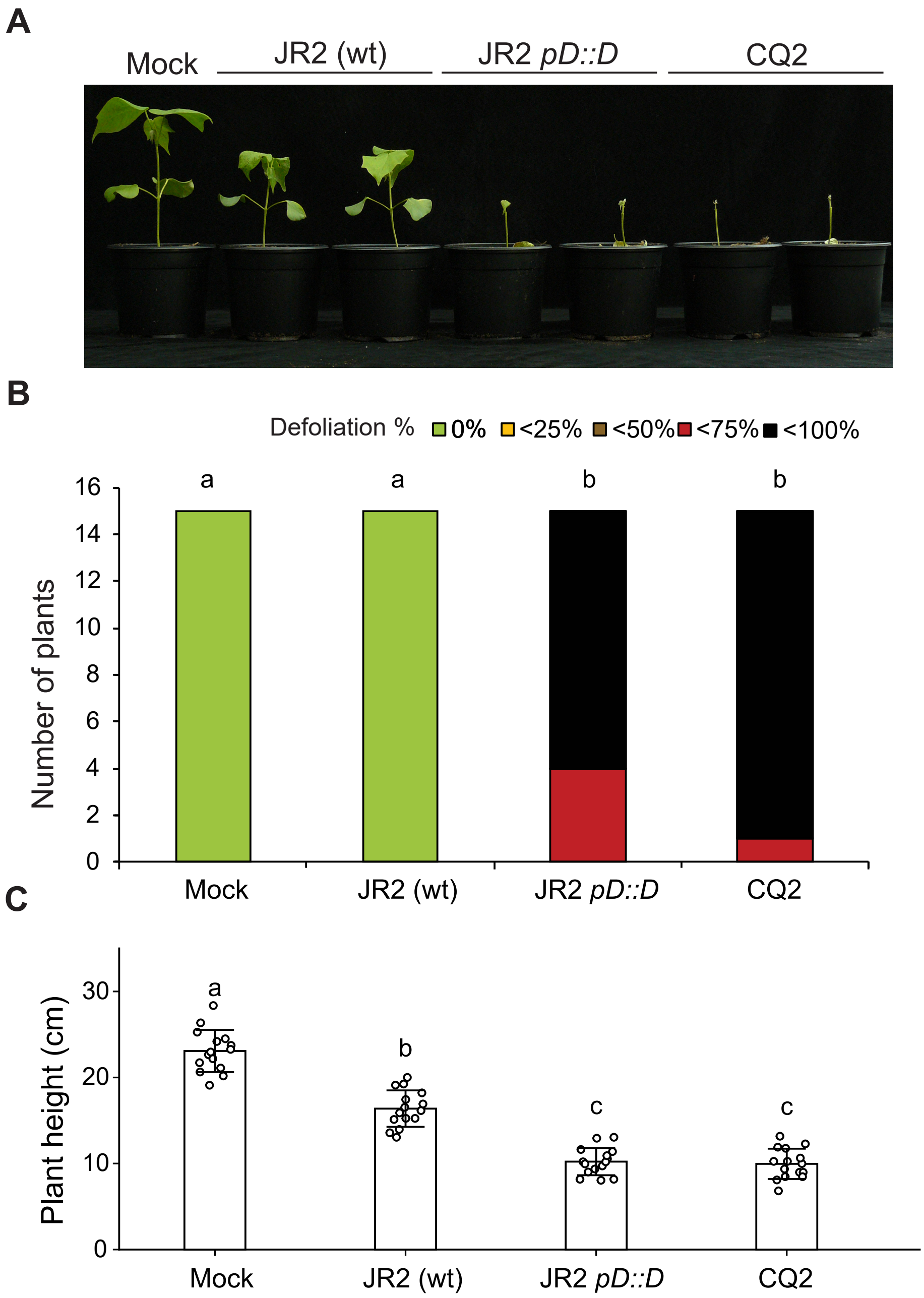

Figure S8. Introduction of *D* gene in ND pathotype strain results in defoliation symptoms. (A) Typical phenotype of cotton (cv. Xinluzao63) plants that were mock-inoculated or inoculated with ND pathotype strain JR2, one *D* expression transformant of JR2 (JR2 p*D*::*D*) and D pathotype strain CQ2 at 28 days post inoculation (dpi). (B) Defoliation was classified as 0 (0% leaves drop off), 1 (<25% leaves drop off), 2 (<50% leaves drop off), 3 (<75% leaves drop off) and 4 (<100% leaves drop off) at 28 dpi. Lower case letters denotate significant grouping evaluated by one-way ANOVA followed by Tukey's HSD post-hoc test (two-sided, adjusted for multiple comparisons within the family of pairwise contrasts; $\alpha = 0.05$; p-Value=<2.58e-51). (C) Plant height as a measure of stunting due to fungal infection. Lower case letters denotate significant grouping evaluated by one-way ANOVA followed by Tukey's HSD post-hoc test (two-sided, adjusted for multiple comparisons within the family of pairwise contrasts; $\alpha = 0.05$; p-Value=<2e-16). Error bars represent the standard error.

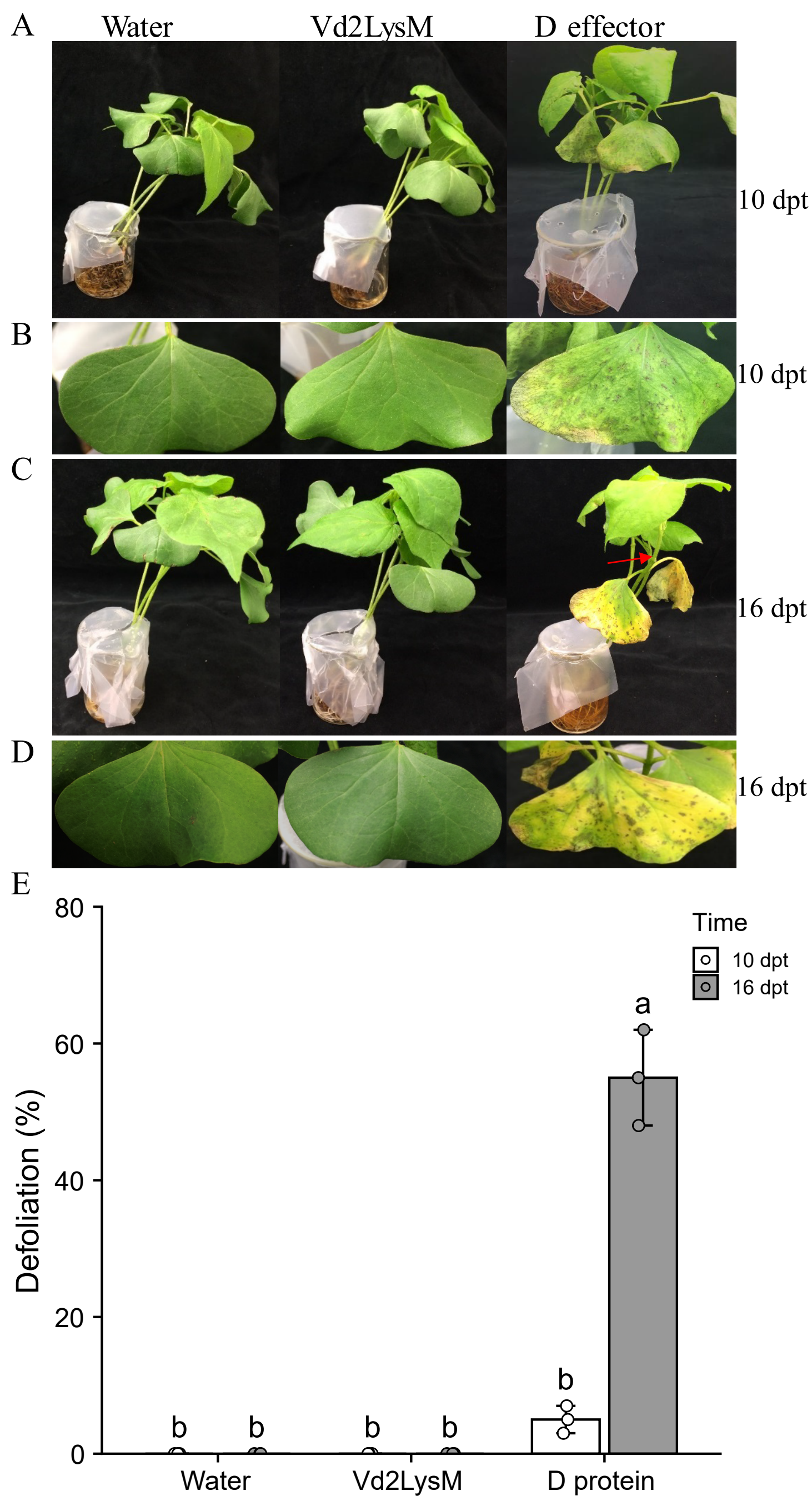

Figure S9. D effector protein induces cotton defoliation *in vitro*. (A-D) Typical appearance of cotton seedlings (cv. Xinluzao63) treated with D effector protein at 10 days (A-B) and 16 days (C-D). Note, marginal chlorosis and wilting symptoms on cotyledons at 10 days (B) and severe chlorosis, and leaf drop off (red arrow; D). (E) Bars represent the average defoliation of two biological replicates with standard deviation. Lower case letters denotate significant grouping evaluated by one-way ANOVA followed by Tukey's HSD post-hoc test (two-sided, adjusted for multiple comparisons within the family of pairwise contrasts; α = 0.05; p-Value=<2e-16). Experiments were repeated twice independently with similar results.

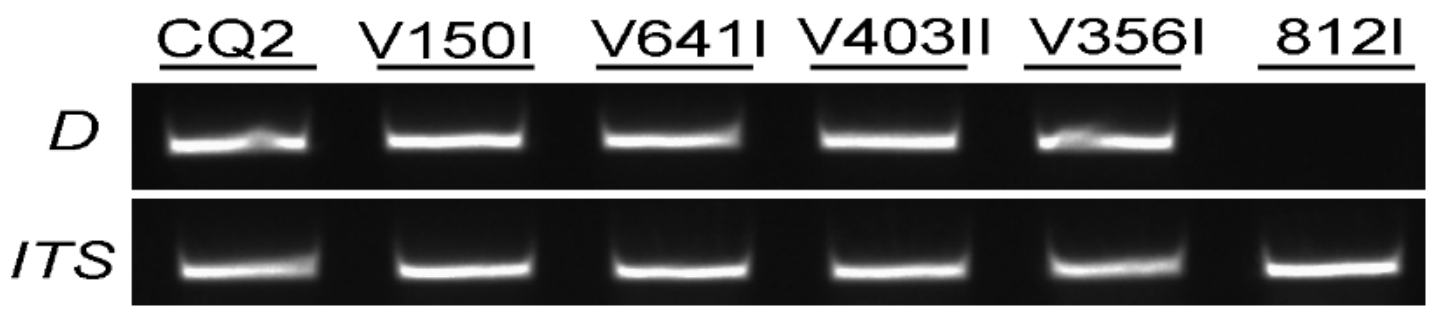

Figure S10. PCR detection of *D* gene in *V. dahliae* strains. Amplification of *D* gene fragment (from left to right) from genomic DNA in CQ2, V150I, V641I, V403I, V356I, 812I. As an endogenous control, a fragment of the *Verticillium* ITS region was amplified.

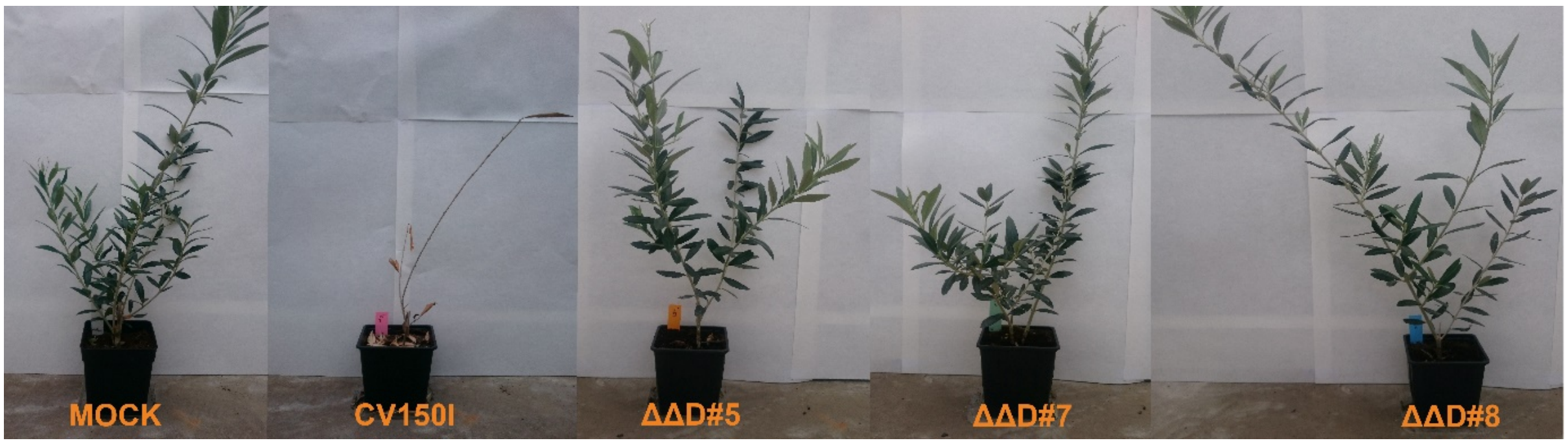

Figure S11. The D effector is responsible for olive defoliation. Typical phenotype of olive (cv. Picual) upon mock-inoculation or inoculation with wild type strain CV150I (WT), and three ΔΔ*D* mutants (ΔΔ*D* #5, ΔΔ*D* #7 and ΔΔ*D* #8) at 132 days post inoculation (dpi).

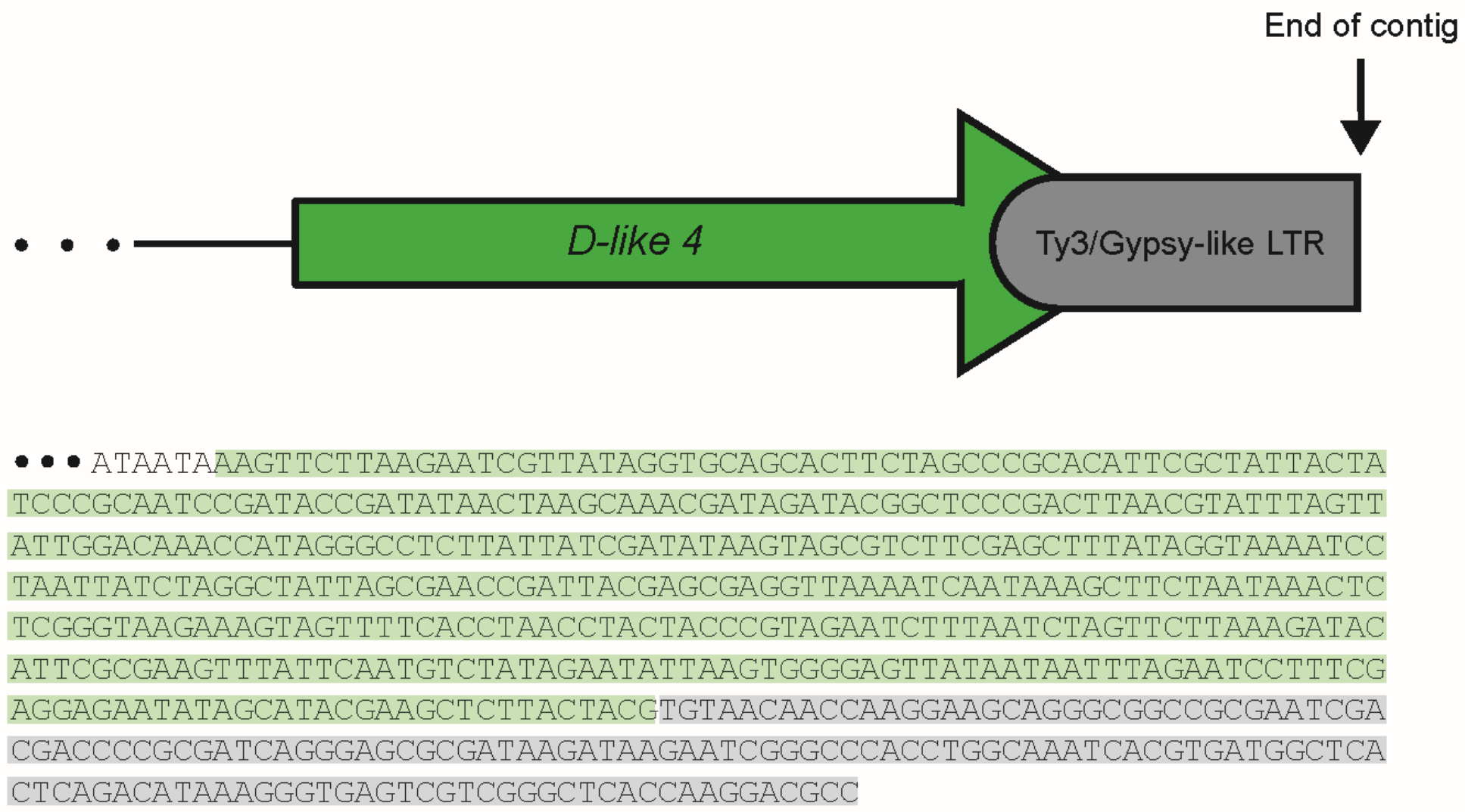

Figure S12. The *D-like 4* allele is disrupted by a retrotransposon of the family Ty3/Gypsy-like. Schematic representation of the *D-like4* allele, showing the interruption of its sequence (represented in green) by a retrotransposon of the family Ty3/Gypsy-like (represented in grey). Only a short portion (174 bp) of the retrotransposon is present before the end of the contig in which the D-like 4 allele is present in both *V. dahliae* strains that carry this allele (V574 and V700).

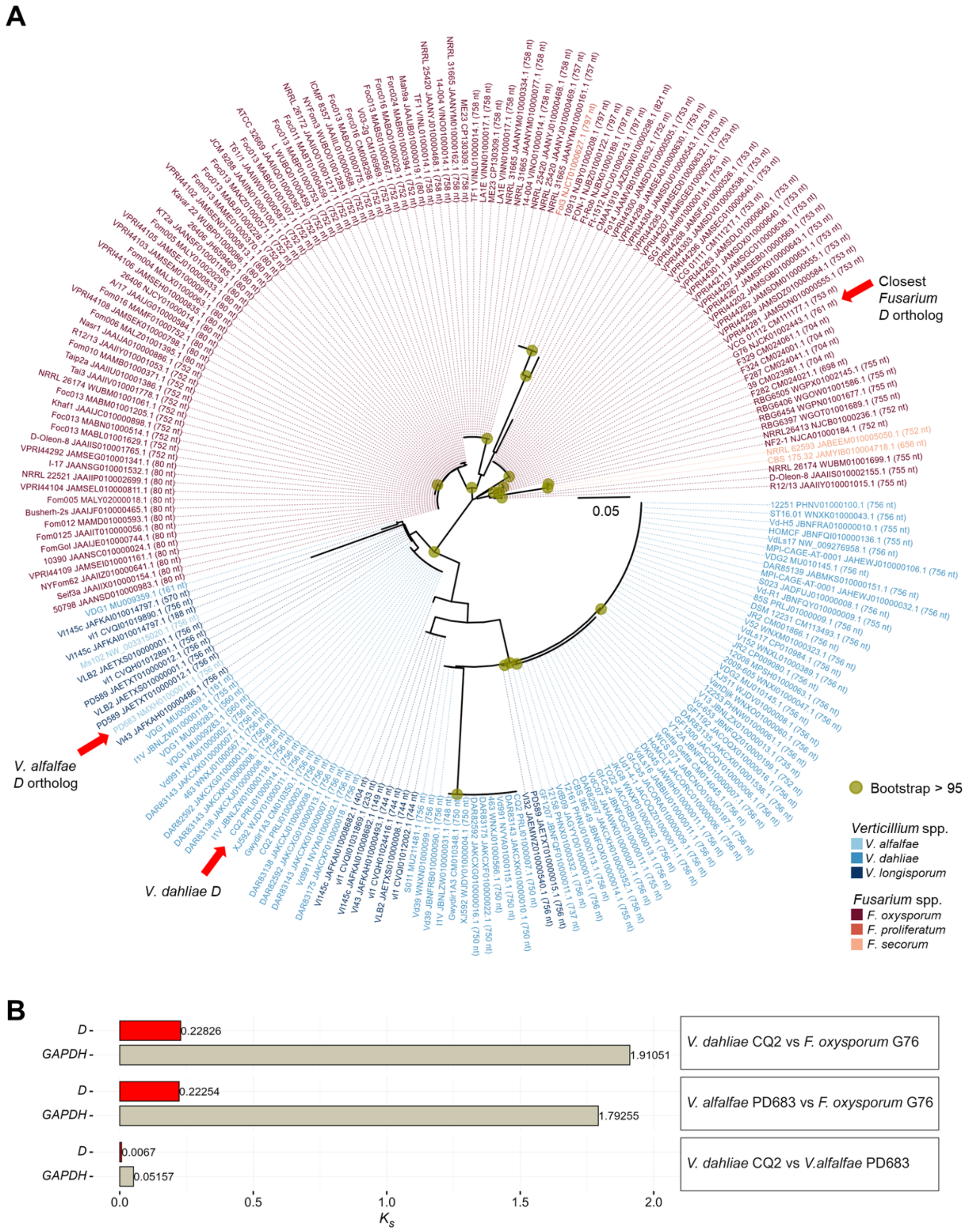

Fig. S13. *D* gene horizontal transfer. (A) Maximum-likelihood phylogenetic tree of *D* homologs inferred using a nucleotide substitution model. Scale bars indicate nucleotide substitutions per site. Circles at nodes denote bootstrap support values >95% based on 1,000 replicates. (B) Rates of synonymous substitutions per synonymous site (K_s) between species. The *D* homologs (red bars) selected for testing are indicated by red arrows in (A). Glyceraldehyde 3-phosphate dehydrogenase (*GAPDH*) genes (grey bars) from the same strains were used as vertically inherited core housekeeping gene references.

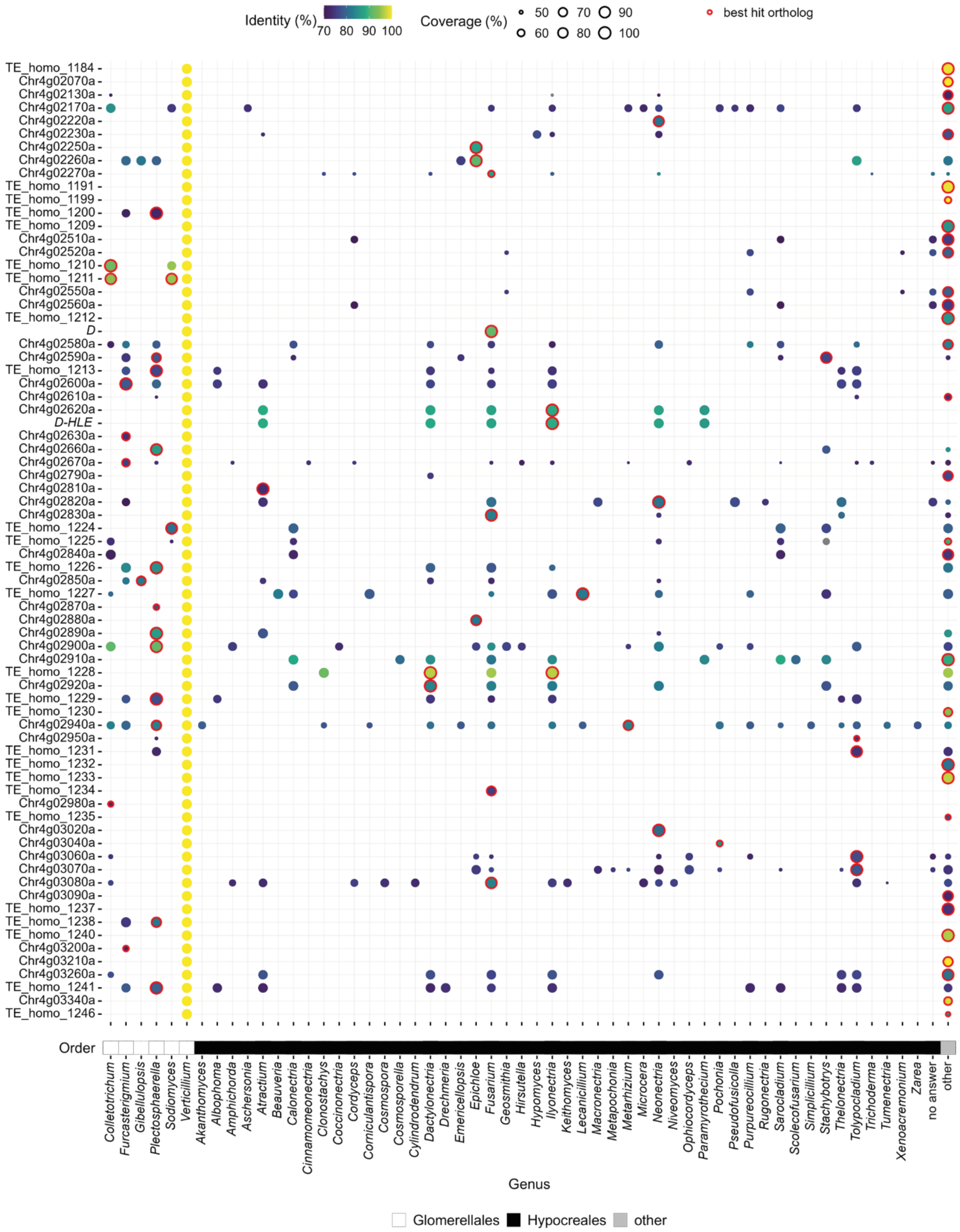

Fig. S14. Distribution of homologs of *Ar1h2 Starship* cargo genes and transposable elements (TEs) across Pezizomycotina. Cargo elements from the *Ar1h2 Starship* in *V. dahliae* strain CQ2 were used as queries. Circles indicate the coverage and sequence identity of the best hits in each genus within the orders Glomerellales and Hypocreales, as well as in all other genera. Best hits across all genera excluding *Verticillium* are outlined in red.

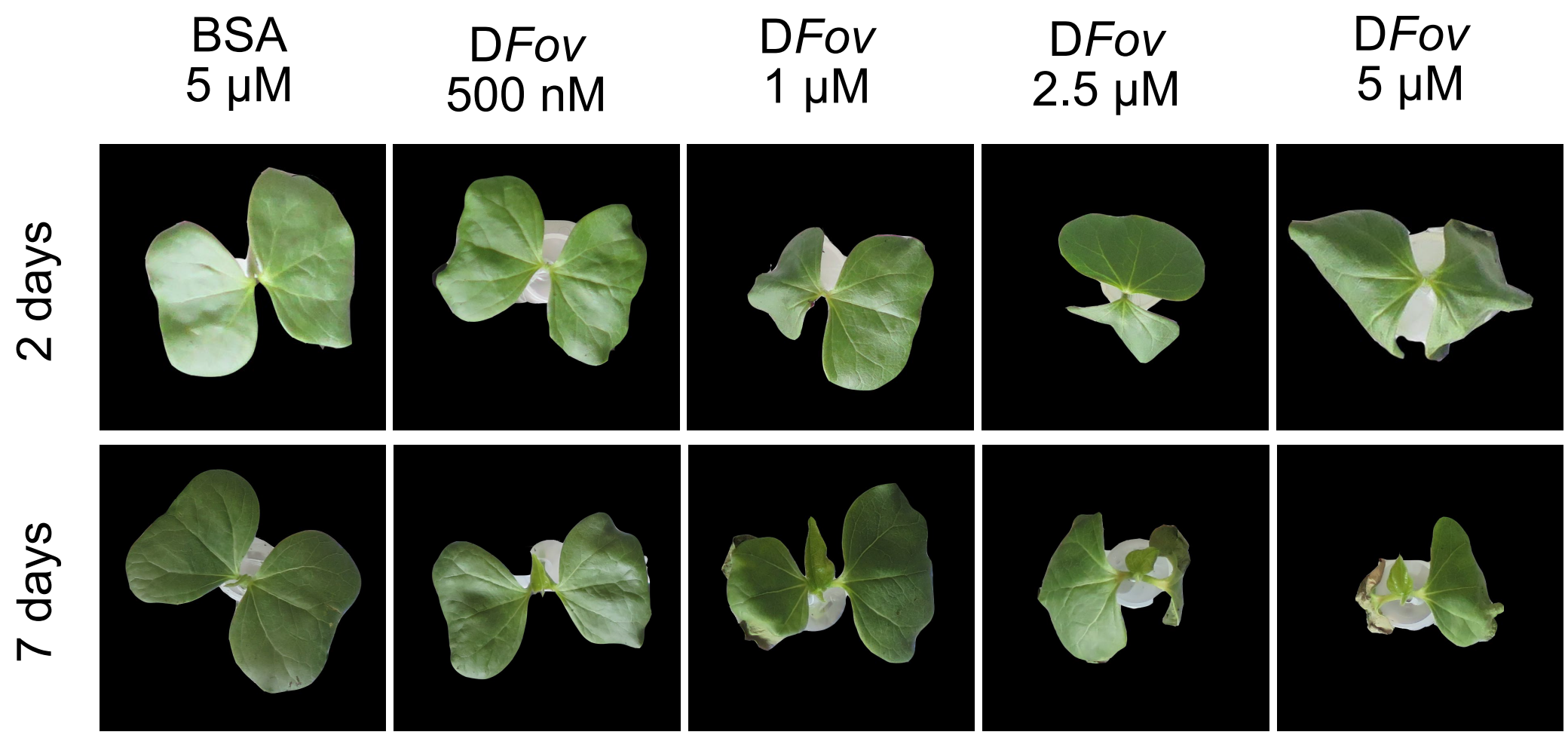

Fig. S15. Typical appearance of cotton seedlings (cv. Xinluzao63) upon application of a concentration range of heterologously produced D*Fov* effector from *Fusarium oxysporum* f. sp. *vasinfectum* (5 μM) at 2 and 7 days post treatment. Visible phenotypic effects are observed at concentrations >1 μM. Bovine serum albumin (BSA; 5 μM) was used as control.

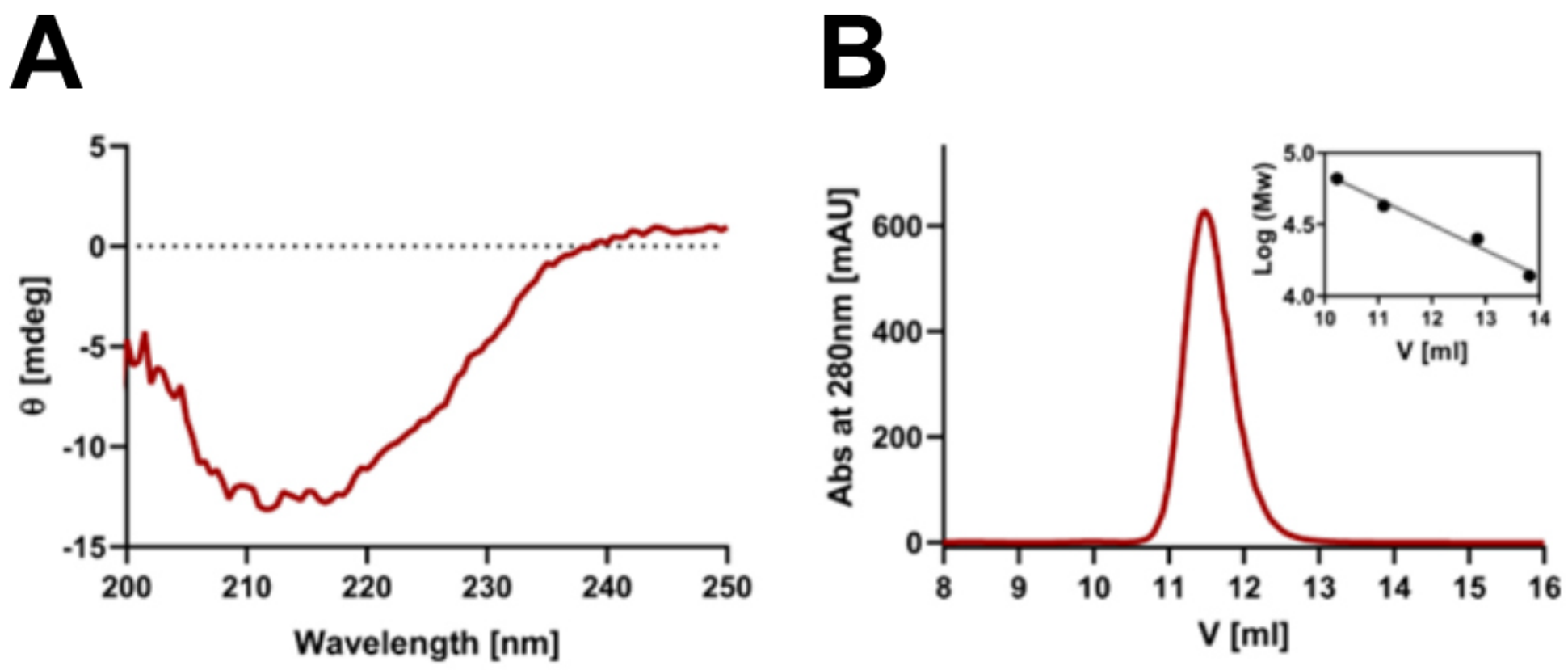

Figure S16. Circular dichroism spectroscopy and size exclusion chromatography analysis of the D*Fov* protein. (A) Far-UV CD spectra of *E. coli*-produced D*Fov* protein (0.2 mg/ml) in 20 mM Tris-HCl pH 7.5, 150 mM NaCl buffer. The spectrum reveals that the recombinant protein is well folded with a prevalently beta-sheet secondary structure. (B) Size exclusion chromatographic elution profile of purified D*Fov*. The protein, loaded on a Superdex 75 10/300 column, eluted as a single sharp peak at 11.7 ml, corresponding to an apparent molecular weight of 35 kDa. Considering that the purified protein, including the affinity tag, has a predicted Mw of 26.6 kDa, this value indicates that D*Fov* is monomeric and displays an elongated shape. *Inset*: the column was calibrated by running the following protein standards: albumin (66.5 kDa), ovalbumin (43 kDa), chymotrypsinogen (25 kDa) and ribonuclease A (13.7 kDa).

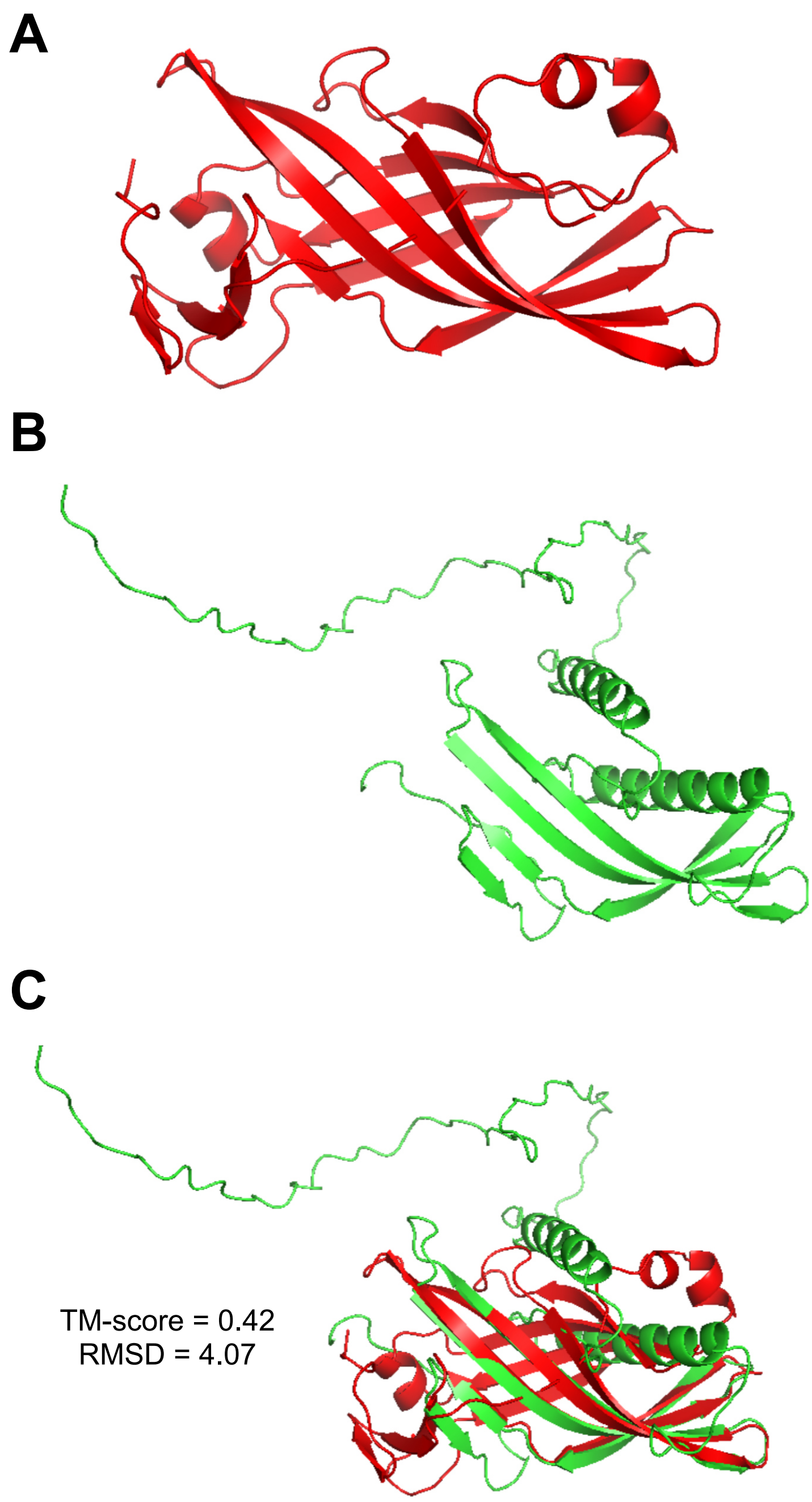

Figure S17. The AlphaFold3-predicted D*Fov* model diverges from the D*Fov* protein structure determined with X-ray crystallography. (A) D*Fov* crystal structure, determined by X-ray crystallography. (B) D*Fov* model predicted with AlphaFold3. (C) Structural alignment of the D*Fov* crystal structure and the AlphaFold3 model. TM-score indicates topological similarity between two protein structures reported on a scale from 0 to 1, where 1 indicates a perfect match, while the RMSD score indicates the average deviation between the corresponding atoms of two proteins with values lower than 2 indicating significant similarity between structures.

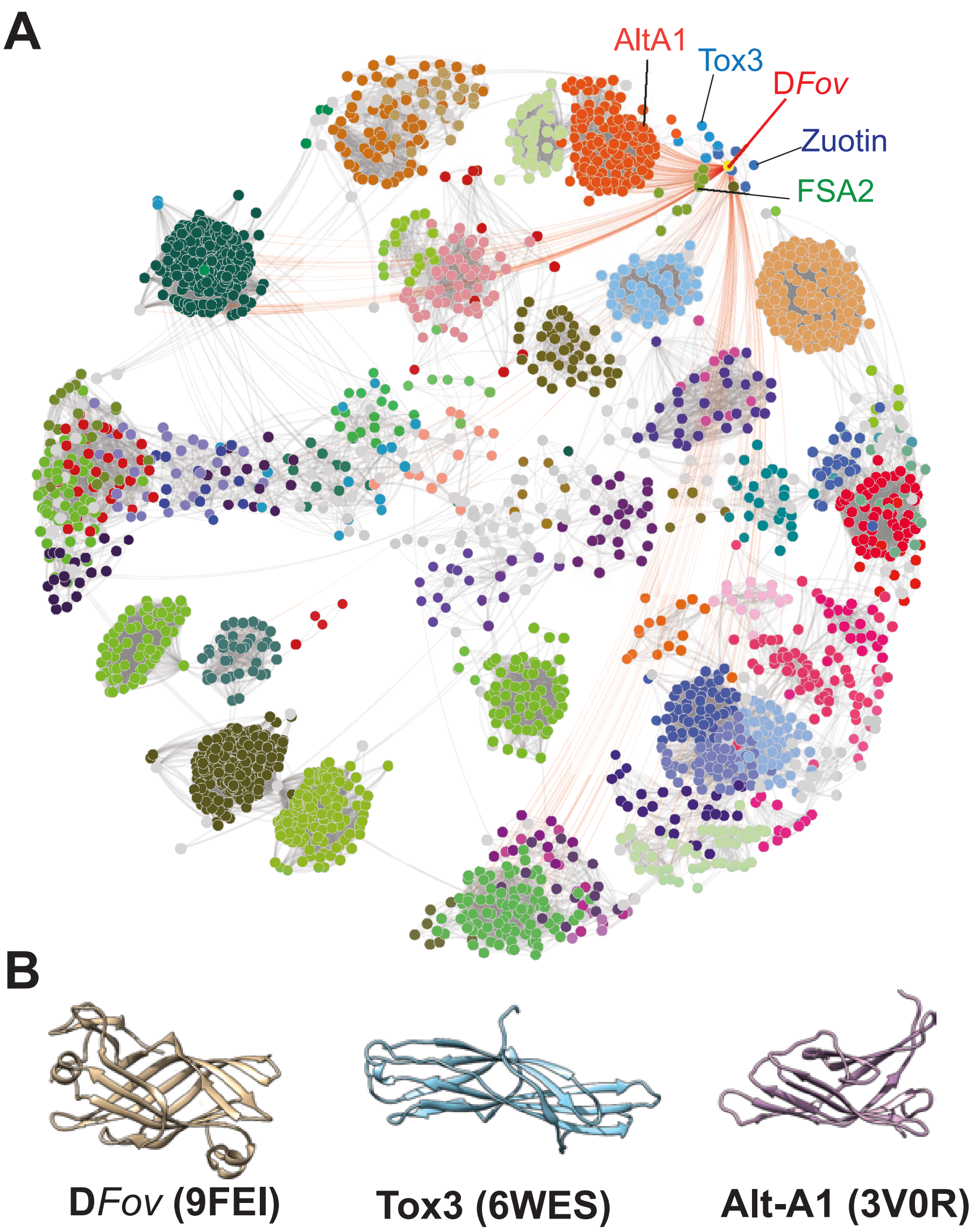

Figure S18. D*Fov* is a structurally unique effector. (A) Structural similarity network based on pairwise DALI Z-scores. Each dot represents a protein structure while different colors represent different structural families. The D*Fov* protein structure is indicated with as well as effector proteins that are somewhat similar, including Alt-A1, Tox3, Zuotin and FSA2. (B) D*Fov*, Tox3 and Alt-A1 protein structures. The template modelling (TM) score and the root-mean-square deviation (RMSD) in comparison to the D*Fov* structure are provided.

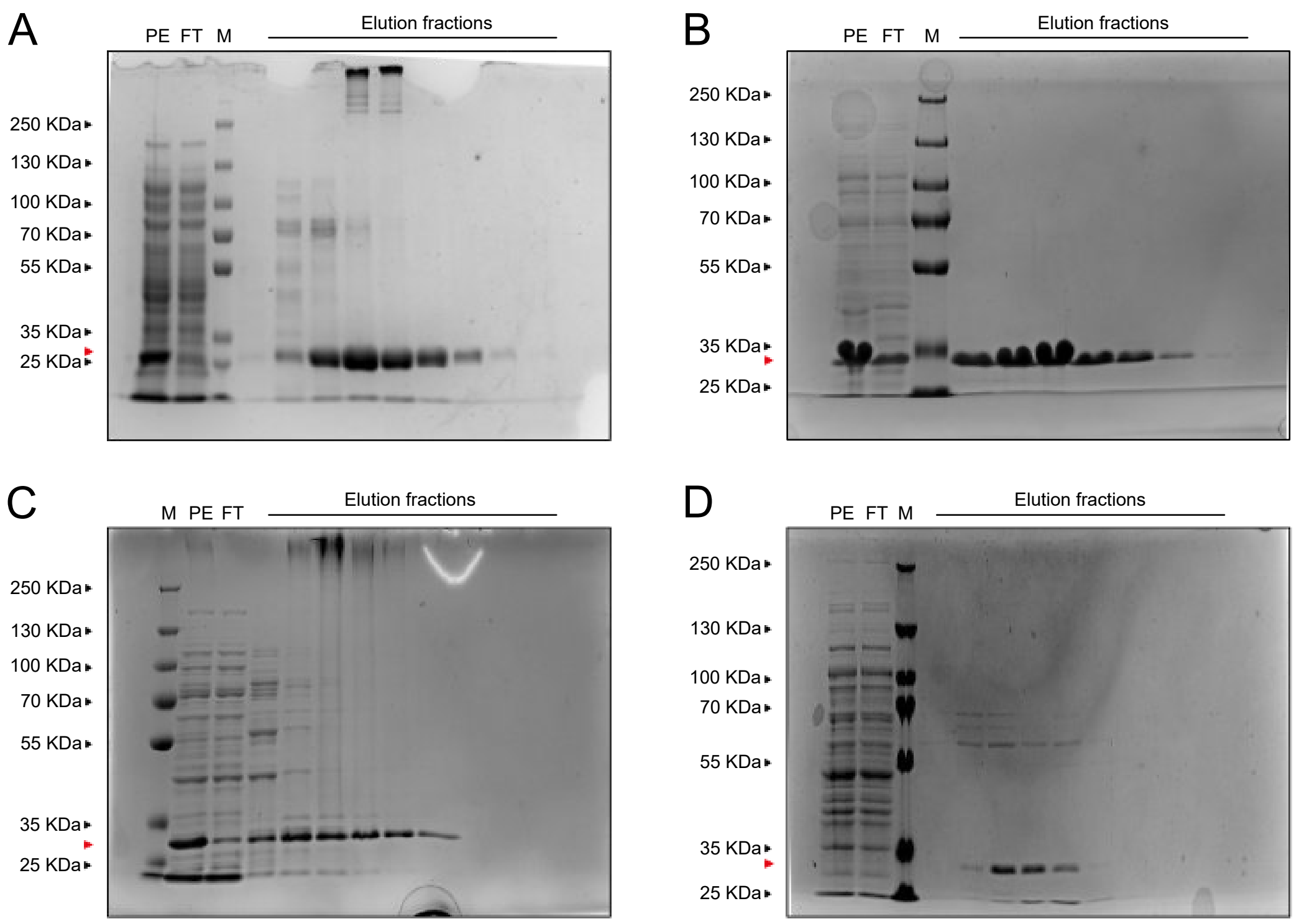

Figure S19. Heterologously produced D protein homologs. SDS-PAGE of affinity-purified D protein homologs: (A) DForc, (B) DFov, (C) VdCQ2 D effector, and (D) VdJR2 D-like 1. The gels show protein fractions at different purification steps: total protein extract (PE) after bacterial lysis, the flow-through (FT) fraction containing unbound proteins after passing the extract through the column, and purified elution fractions. PageRuler Plus Prestained protein ladder was used as a molecular weight marker (M). The red arrow indicates protein bands corresponding to the D effector and its homologs (27 kDa).

Table S1. ***V. dahliae*** **strains used in this study.**

Strain	Sequencing platform	Reference	Origin	Geographical location
HN	Illumina	Xu et al., 2012	Cotton	China
cd3	Illumina	Xu et al., 2012	Cotton	China
VdLs17	PacBio	Faino et al., 2015	Lettuce	USA
JR2	PacBio	Faino et al., 2015	Tomato	Canada
Vd57	Illumina	This study	Strawberry	Germany
V152	Illumina	Kombrink et al., 2017	Oak	Hungary
Vd52	Illumina	Kombrink et al., 2017	Pepper	Austria
van Dijk	Illumina	Kombrink et al., 2017	Chrysanthemum	The Netherlands
BP2	Illumina	Zhang et al., 2012	Cotton	China
ST16.01	Illumina	This study	Cotton	Syria
2009-605	Illumina	This study	Bell pepper	Ukraine
V4	Illumina	Keykhasaber, 2017	Olive	Spain
V200I	Illumina	This study	Strawberry	Germany
CBS38166	Illumina	de Jonge et al., 2012	Tomato	Canada
DVD-S26	Illumina	de Jonge et al., 2012	Soil	Canada
VdLS16	Illumina	de Jonge et al., 2012	Lettuce	USA
ST14.01	Illumina	de Jonge et al., 2012	Pistachio	USA
DVD-S29	Illumina	de Jonge et al., 2012	Soil	Canada
DVD-31	Illumina	de Jonge et al., 2012	Tomato	Canada
DVD-161	Illumina	de Jonge et al., 2012	Tomato	Canada
DVD-S94	Illumina	de Jonge et al., 2012	Soil	Canada
DVD-3	Illumina	de Jonge et al., 2012	Potato	Canada
V192I	Illumina	This study	Cotton	Spain
JKG8	Illumina	Kombrink et al., 2017	Potato	The Netherlands
85S	PacBio	This study	Sunflower	France
Vd39	Illumina	This study	Sunflower	Germany
V574	Illumina	Milgroom et al., 2014	Artichoke	Spain
v700	Illumina	Milgroom et al., 2014	Artichoke	Spain
v679	Illumina	Milgroom et al., 2014	Artichoke	Spain
T9	Illumina	Keykhasaber, 2017	Cotton	USA
V781I	Illumina	This study	Olive	Spain
V138I	Illumina	This study	Cotton	Spain
TM6	Illumina	Keykhasaber, 2017	Cotton	China
V117	Illumina	Keykhasaber, 2017	Olive	Spain
V991	Illumina	Zhang et al., 2012	Cotton	China
CQ2	PacBio	Depotter et al., 2019	Cotton	China
ST100	Illumina	de Jonge et al., 2012	Soil	Belgium
V76	Illumina	This study	Cotton	Mexico
463	Illumina	This study	Cotton	Mexico
XJ-14	Illumina	Chavarro-Carrero et al., 2021	Not applicable	China
XJ-12	Illumina	Chavarro-Carrero et al., 2021	Not applicable	China
WFA3	Illumina	Chavarro-Carrero et al., 2021	Not applicable	China
V08SY-2	Illumina	Chavarro-Carrero et al., 2021	Not applicable	China
HNA4	Illumina	Chavarro-Carrero et al., 2021	Not applicable	China
CFA3	Illumina	Chavarro-Carrero et al., 2021	Not applicable	China
89	Illumina	Chavarro-Carrero et al., 2021	Not applicable	China

GF1192	Nanopore	Chavarro-Carrero et al., 2021	Not applicable	Japan
HOMCF	Nanopore	Chavarro-Carrero et al., 2021	Not applicable	Japan
VT-2A	Nanopore	Chavarro-Carrero et al., 2021	Not applicable	Japan
GF-Cb5	Nanopore	Chavarro-Carrero et al., 2021	Not applicable	Japan
GF-Ca2	Nanopore	Chavarro-Carrero et al., 2021	Not applicable	Japan
GF1207	Nanopore	Chavarro-Carrero et al., 2021	Not applicable	Japan
TO22	Nanopore	Chavarro-Carrero et al., 2021	Not applicable	Japan
UD1-4-1	Nanopore	Chavarro-Carrero et al., 2021	Aralia cordata	Japan
12008	Illumina	Harrison-Lab NIAB-EMR	Strawberry	United Kingdom
12253	Illumina	Harrison-Lab NIAB-EMR	Fragaria vesca	United Kingdom
12251	Illumina	Harrison-Lab NIAB-EMR	Fragaria vesca	United Kingdom
12161	Illumina	Harrison-Lab NIAB-EMR	Fragaria vesca	United Kingdom
12158	Illumina	Harrison-Lab NIAB-EMR	Fragaria vesca	United Kingdom
V1	Nanopore	Chavarro Carrero, E. A. (2024)	Rose	Mexico
V13	Nanopore	Chavarro Carrero, E. A. (2024)	Rose	Mexico
Vmx	Nanopore	Chavarro Carrero, E. A. (2024)	Rose	Mexico
I1V	Nanopore	Chavarro Carrero, E. A. (2024)	Rose	Mexico
EU7	Nanopore	Chavarro Carrero, E. A. (2024)	Rose	The Netherlands
EU35	Nanopore	Chavarro Carrero, E. A. (2024)	Rose	The Netherlands
CBS385.49	Nanopore	Chavarro Carrero, E. A. (2024)	Rose	The Netherlands
CBS809.97	Nanopore	Chavarro Carrero, E. A. (2024)	Rose	The Netherlands

Supplementary Table 2. X-ray diffraction data collection and refinement statistics for the *F. oxysporum* f. sp. *vasinfectum* DFov protein homolog.

**Data Collection**	
PDB id	9FEI
Synchrotron, beamline	ESRF – ID23-1
Space group	P1 2_1 1
Cell Dimensions; a-b-c (Å), α–β–γ (°)	27.8-50.0-72.3, 90.00-98.91-90.00
Resolution Range (Å)	71.40-1.47 (1.50-1.47) [a]
R_{merge}	0.083 (0.73)
Half-set correlation CC(1⁄2) (%)	0.993 (0.775)
<I/σI>	12.5 (2.3)
Completeness (%)	99.9 (100.0)
N. reflection (total)	219281 (10336)
Multiplicity	6.6 (6.4)
Wilson B-factor ($Å^2$)	20,28
Anomalous completeness (%)	98.6 (99.1)
Anomalous multiplicity	3.4 (3.3)
|DANO|/sd(DANO)	1.065 (0.657)
**Refinement**	
N. of unique reflections used in refinement	33139 (3273)
N. of reflections used for R-free	1648 (153)
R-work / R-free (%)	0.162 (0.259) / 0.188 (0.275)
F_o,F_c correlation	0,97
Average B-factor all atoms ($Å^2$)	27,94
Protein	26,6
Ligands	35,72
Water	37,91
Bonds RMSD	
Length (Å)	0,013
Angle (°)	1,83
Ramachandran plot (% / n. residues)	
Favored	98.1 / 184
Allowed	1.1 / 4
Outliers	0.0 / 0
Clashscore	5,01

[a] Statistics for the highest-resolution shell are shown in parentheses

Supplementary Table 3. Coordinates of D and GAPDH coding sequences.

Species and strain	Gene	CDS	Contig accession	Start (1-base)	End	Strand
Verticillium dahliae CQ2	D	1	PRLI01000014.1	7006	6845	Minus
Verticillium dahliae CQ2	D	2	PRLI01000014.1	6798	6247	Minus
Verticillium dahliae CQ2	GAPDH	1	PRLI01000007.1	2006258	2006129	Minus
Verticillium dahliae CQ2	GAPDH	2	PRLI01000007.1	2005930	2005043	Minus
Verticillium alfalfae PD683	D	1	NMXH01000011.1	8311	8149	Minus
Verticillium alfalfae PD683	D	2	NMXH01000011.1	8103	7552	Minus
Verticillium alfalfae PD683	GAPDH	1	NMXH01000004.1	2036356	2036485	Plus
Verticillium alfalfae PD683	GAPDH	2	NMXH01000004.1	2036687	2037574	Plus
Fusarium oxysporum f. sp. gladioli G76	D	1	NJCK01002443.1	1941	1774	Minus
Fusarium oxysporum f. sp. gladioli G76	D	2	NJCK01002443.1	1728	1177	Minus
Fusarium oxysporum f. sp. gladioli G76	GAPDH	1	NJCK01000083.1	366986	366858	Minus
Fusarium oxysporum f. sp. gladioli G76	GAPDH	2	NJCK01000083.1	366521	365634	Minus

Supplementary Table 4. Genome assemblies used for synteny detection.

Species and strain	Database	Accession
Verticillium dahliae JR2	EnsemblFungi	VDAG_JR2v.4.0
Verticillium dahliae CQ2	Zenodo	https://doi.org/10.5281/zenodo.15450312
Verticillium dahliae VD991	NCBI GenBank	GCA_030450075.1
Verticillium longisporum VLB2	Zenodo	https://doi.org/10.5281/zenodo.15450312
Verticillium alfalfae PD683	Zenodo	https://doi.org/10.5281/zenodo.15450312
Fusarium oxysporum f. sp. *vasinfectum* ME23	NCBI GenBank	GCA_030719095.1
Fusarium oxysporum f. sp. *radicis-cucumerinum* V03-2g	NCBI GenBank	GCA_048164945.1
Fusarium oxysporum f. sp. *radicis-cucumerinum* Forc016	NCBI GenBank	GCA_001702695.2
