# Supplementary material for: A structurally unique effector shared between vascular wilt fungi drives cotton and olive defoliation
Source: Nat Commun. 2026 Jun 21;17:7818. doi: 10.1038/s41467-026-74504-z (PMC13439237; doi:10.1038/s41467-026-74504-z)
Supplement: Supplementary file 4 [file 41467_2026_74504_MOESM2_ESM.pdf]

**Description of Additional Supplementary Files**

Supplementary Data 1. D protein hits in the genomes of fungal species.

Supplementary Data 2. Summary of blastn hits in *Fusarium* genomes queried with Ar1h2 Starship cargo genes.

Supplementary Data 3. Z-score for all proteins matching Dfov protein using the DALI database.

Supplementary Data 4. Primers used in this study.

Supplementary Data 5. Coordinates of captain/captain-like genes in *Fusarium* genomes that carry D orthologs.

Supplementary Data 6. Coordinates of genes and transposable elements (TEs) in *Verticillium dahliae* strain CQ2 for the alien index (AI) caluculation.

Supplementary Data 7. Pezizomycotina genomes used for the alien index calculation.
